# Supplementary material for: aPKC Cycles between Functionally Distinct PAR Protein Assemblies to Drive Cell Polarity
Source: Dev Cell. 2017 Aug 21;42(4):400–415.e9. doi: 10.1016/j.devcel.2017.07.007 (PMC5563072; doi:10.1016/j.devcel.2017.07.007)
Supplement: Document S1. Figures S1–S7 [file mmc1.pdf]

Developmental Cell, Volume *42*

Supplemental Information

aPKC Cycles between Functionally Distinct PAR Protein Assemblies to Drive Cell Polarity

Josana Rodriguez, Florent Peglion, Jack Martin, Lars Hubatsch, Jacob Reich, Nisha Hirani, Alicia G. Gubieda, Jon Roffey, Artur Ribeiro Fernandes, Daniel St Johnston, Julie Ahringer, and Nathan W. Goehring

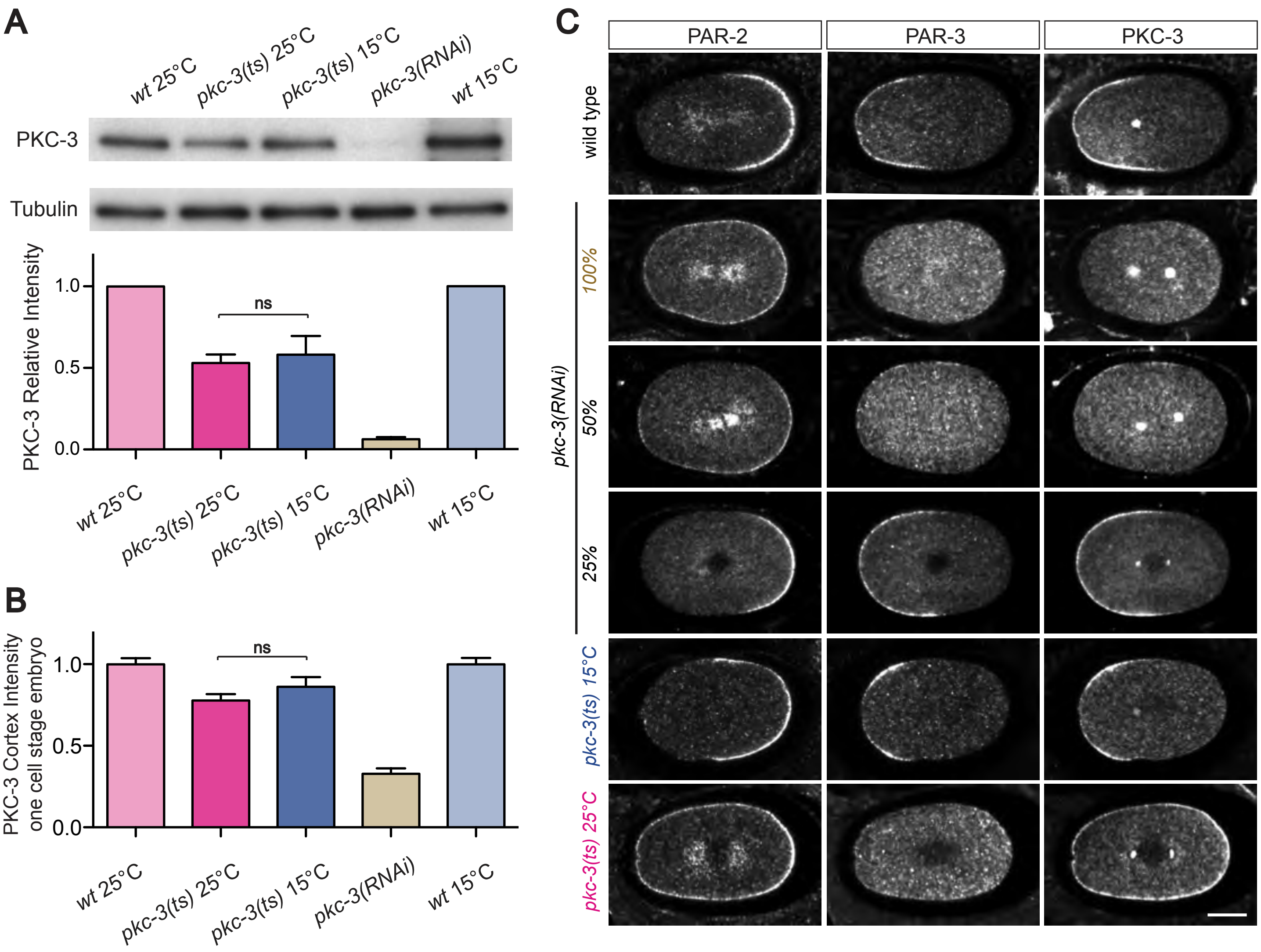

Figure S1. PKC-3 (D386V) causes a temperature sensitive polarity phenotype distinct from reduction in PKC-3 levels, Related to Figure 1

(A) Embryo extract blots at the indicated conditions showing PKC-3 and α-tubulin (loading control). Intensity of PKC-3 (loading-corrected) in *pkc-3(ts)*(D386V) and *pkc-3(RNAi)* embryos quantified relative to their corresponding wild type temperature condition (mean±SEM). *pkc-3(RNAi)* was performed at 25ºC. Note that *pkc-3(ts)* at the restrictive and permissive temperatures show similar reduction in PKC-3 protein amounts compared to controls. *pkc-3(ts)* 25ºC (n=5), *pkc-3(ts)* 15ºC (n=3), *pkc-3(RNAi)* (n= 4).

(B) Quantification of zygote PKC-3 cortical intensity determined by confocal immunofluorescence. PKC-3 cortex intensity of *pkc-3(ts)* embryos are plotted relative to PKC-3 cortical levels of control zygotes (mean±SEM). Control (TY3558) and *pkc-3(ts)* (WM150) zygotes were processed on the same slide to facilitate direct comparisons, with control zygotes identifiable by expression of GFP fusions to histone and β-tubulin. Embryos subjected to *pkc-3(RNAi)* were mounted on a separate slide but processed in parallel and imaged under identical conditions. Note that the results are broadly similar to what is seen in western blots. wt 25ºC (n=23), *pkc-3(ts)* 25ºC (n=31), *pkc-3(ts)* 15ºC (n=25), *pkc-3(RNAi)* (n= 10) and wt 15ºC (n=27).

(C) Representative midsection confocal images of wild type, *pkc-3(RNAi)* and *pkc-3(ts)* metaphase zygotes, immunostained for PAR-2, PAR-3 and PKC-3. Note that although PKC-3 levels are similar in *pkc-3(ts)* at 15ºC and 25ºC (A and B), only *pkc-3(ts)* at 25ºC shows a clear polarity defect. *pkc-3(ts)* at 15ºC appears very similar to the phenotype observed under a weak knock down of PKC-3 (*pkc-3(RNAi)* 25%), including the appearance of a small, transient anterior PAR-2 domain during the establishment phase (data not shown). Scale bar: 10μm.

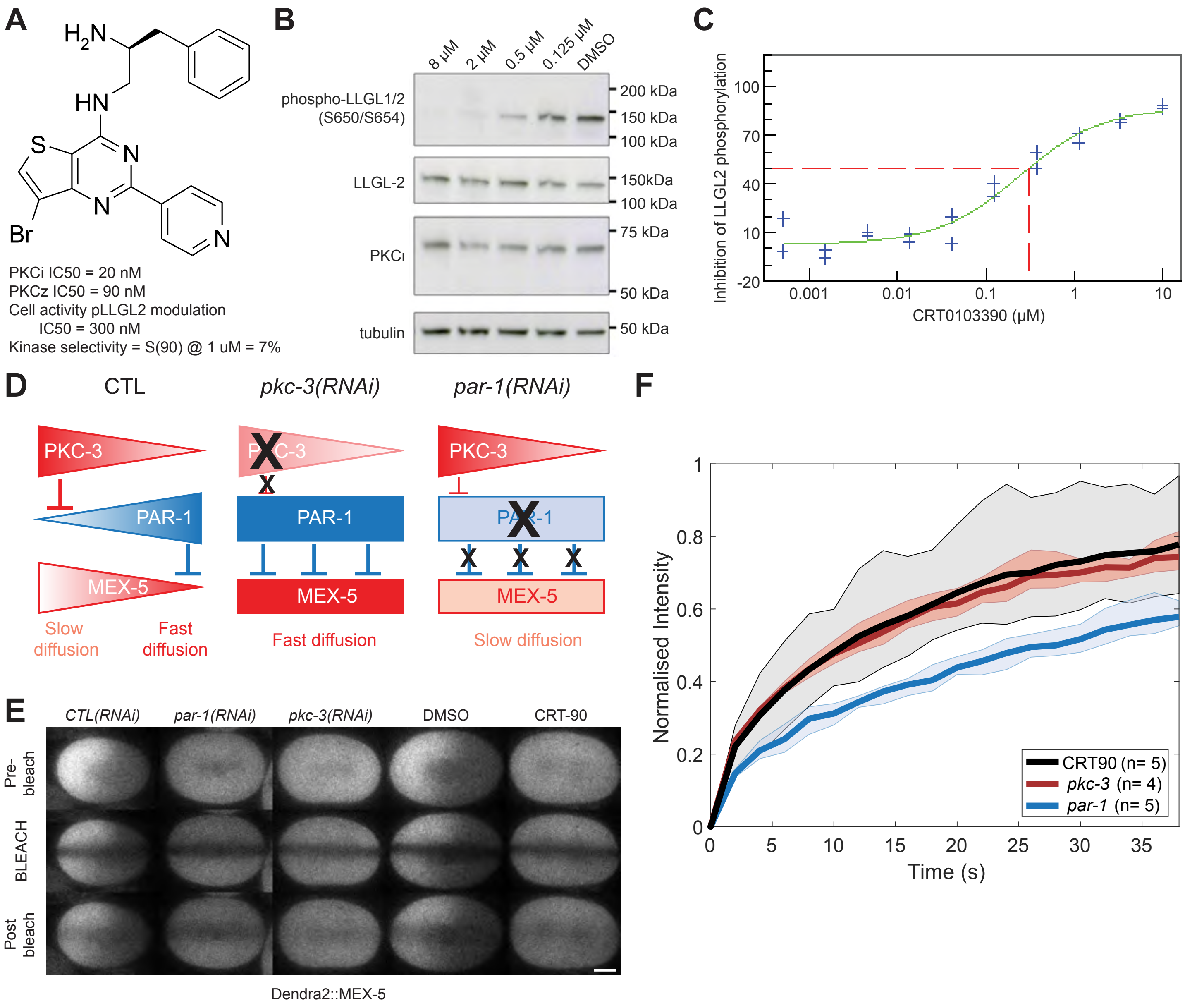

Figure S2. CRT0103390 inhibits atypical PKC without affecting PAR-1, Related to Figure 1

(A) Structure of CRT0103390 and key enzymatic data. Kinase selectivity represents the percent of kinases out of a panel of 442 diverse kinases inhibited by >90%.

(B) CRT0103390 inhibits LLGL2 phosphorylation in HEK-293 cells, co-transfected with LLGL2-FLAG and PKCι, after 1 hr of treatment as assayed by immunoblot analysis.

(C) IC50 curve of LLGL2 phosphorylation by CRT0103390 measured by ELISA.

(D) The diffusive state of MEX-5 along the antero-posterior zygote axis is regulated by PAR-1, which is epistatic to PKC-3. In *pkc-3(RNAi)* embryos, PAR-1 is uniformly active, resulting in uniform, fast diffusion of MEX-5. Conversely, in *par-1(RNAi)* or *par-1/pkc-3(RNAi)* embryos, MEX-5 is uniformly slow (Griffin et al., 2011). If CRT90 inhibited both PAR-1 and PKC-3, we would expect uniform slow MEX-5, which we do not observe in (E) and (F).

(E) Selected midsection confocal images of MEX-5 in control, *par-1(RNAi)*, *pkc-3(RNAi)*, DMSO and CRT90 treated zygotes, before (Pre-bleach), immediately after (BLEACH), or 20 seconds after fluorescence photobleaching of a central stripe along the AP axis (Post-bleach). Scale bar: 10μm.

(F) Pre-bleach-normalized fluorescence intensity recovery of a central region of the embryo, showing that MEX-5 mobility in CRT90-treated embryos closely matches the faster recovery of *pkc-3(RNAi)* embryos compared to *par-1*(*RNAi*). For each condition, mean values are shown (thick line) along with a shaded region indicating the full data range across samples.

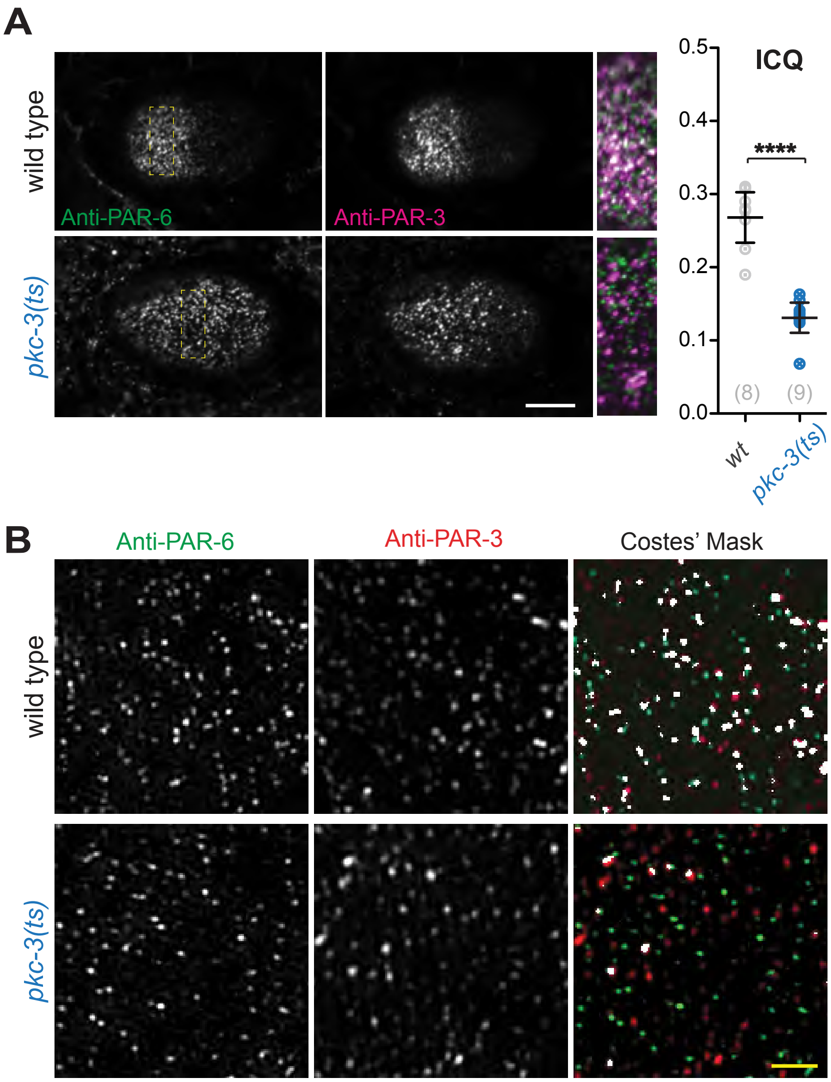

Figure S3. PAR-6 and PAR-3 colocalization is reduced in PKC-3-inhibited embryos, Related to Figure 2

(A) Cortical confocal images of wild type and *pkc-3(ts)* zygotes at late establishment phase stained for PAR-6 and PAR-3. Insets are magnifications of the ROIs delimited by dashed-line rectangles in PAR-6 image and show the overlap (white) of PAR-6 and PAR-3 in the anterior cortex. Scale bar: 10µm. Graphs shows the intensity correlation quotient (Li et al., 2004, Mean±CI 95%) between PAR-6 and PAR-3 in wild type (n=8) and *pkc-3(ts)* (n=9) zygotes.****p<0.0001.

(B) Super resolution images of wild type and *pkc-3(ts)* zygotes of an anterior cortical region stained for PAR-6 and PAR-3. In Costes' Mask images (JaCOP, Fiji) white regions indicate highly probable regions of colocalization (p-value of 100%). Scale bar: 2 µm.

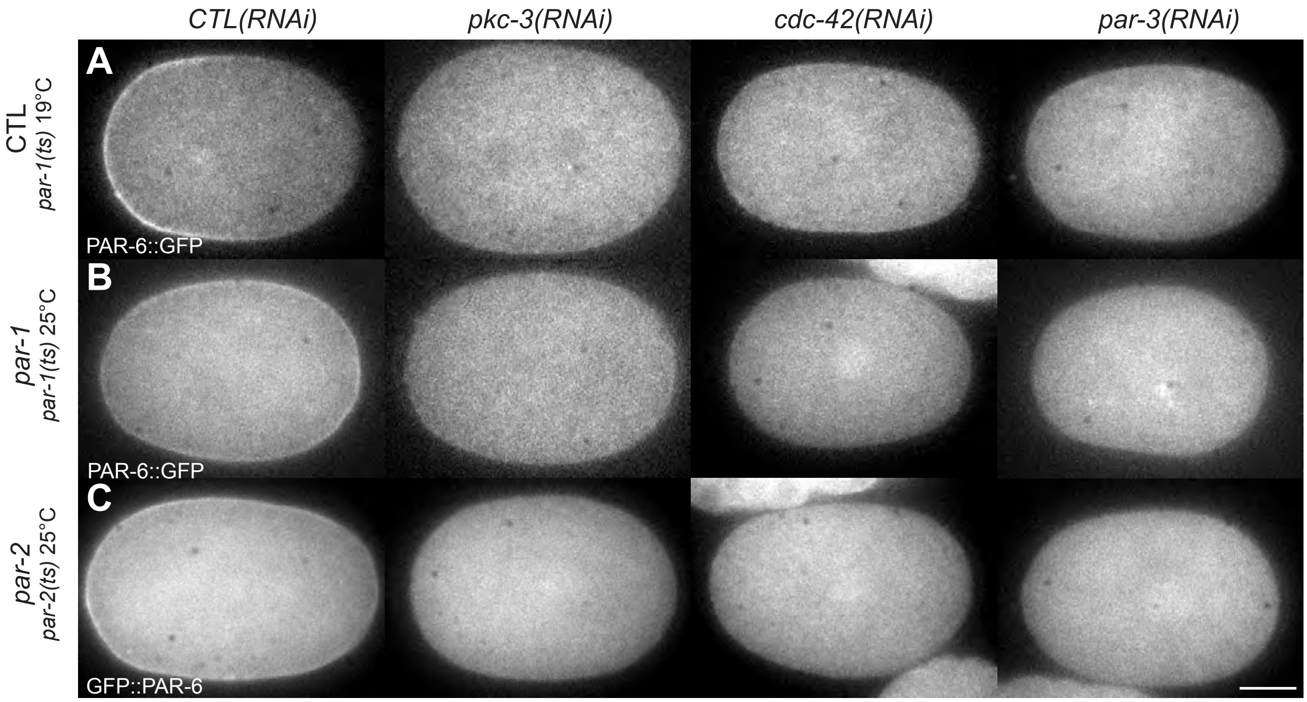

Figure S4. PAR-6 membrane localization depends directly on PAR-3, PKC-3 and CDC-42, Related to Figure 2

(A-C) Representative midsection confocal fluorescent images of PAR-6::GFP (A-B) and GFP::PAR-6 (C) expressing zygotes captured at nuclear envelope breakdown in the respective mutant backgrounds (*par-1(ts)*, A-B; *par-2(ts)*, C) at the permissive (19ºC, A) or restrictive (25ºC, B-C) temperature in combination with RNAi targeting *pkc-3*, *cdc-42* and *par-3* as indicated. Note that at the permissive temperature (CTL, first row), PAR-6 fails to localize to the membrane in *pkc-3(RNAi)*, *cdc-42(RNAi)* or *par-3(RNAi)* embryos. Membrane localization is not rescued when shifted to the restrictive temperature to inactivate PAR-1(B) or PAR-2(C) suggesting the failure of PAR-6 to bind membrane is not due to invasion of PAR-1 / PAR-2 into the anterior. Scale bar:10µm.

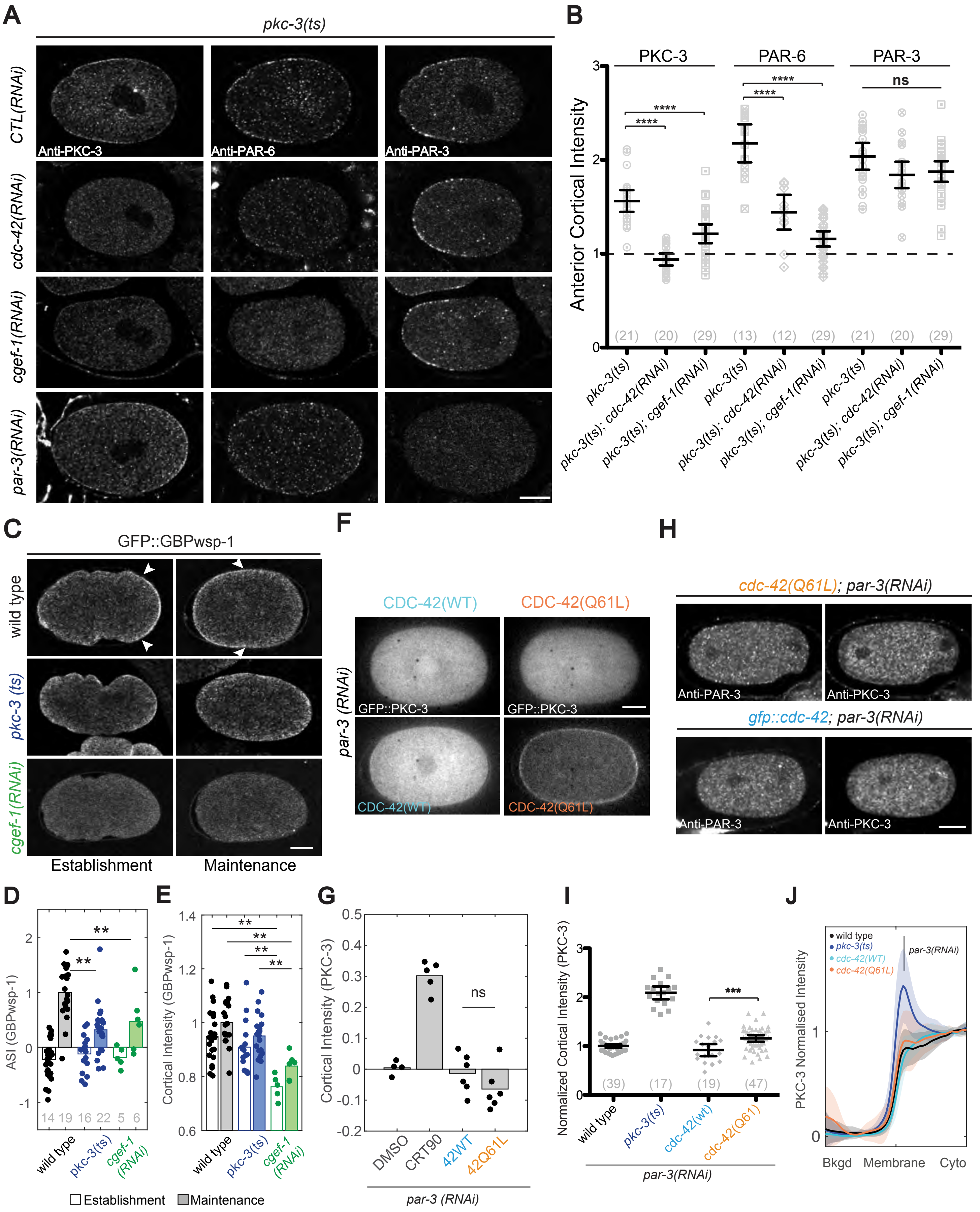
A
pkc-3(ts)
CTL(RNAi)
cdc-42(RNAi)
cgef-1(RNAi)
par-3(RNAi)
Anti-PKC-3
Anti-PAR-6
Anti-PAR-3
B
PKC-3
PAR-6
PAR-3
Anterior Cortical Intensity
****
****
****
****
ns
(21) (20) (29) (13) (12) (29) (21) (20) (29)
pkc-3(ts)
pkc-3(ts); cdc-42(RNAi)
pkc-3(ts); cgef-1(RNAi)
C
GFP::GBPwsp-1
wild type
pkc-3 (ts)
cgef-1(RNAi)
Establishment
Maintenance
F
CDC-42(WT)
CDC-42(Q61L)
par-3 (RNAi)
GFP::PKC-3
H
cdc-42(Q61L); par-3(RNAi)
gfp::cdc-42; par-3(RNAi)
Anti-PAR-3
Anti-PKC-3
D
ASI (GBPwsp-1)
**
**
14 19 16 22 5 6
wild type
pkc-3(ts)
cgef-1 (RNAi)
Establishment
Maintenance
E
Cortical Intensity (GBPwsp-1)
**
**
**
**
G
Cortical Intensity (PKC-3)
ns
DMSO
CRT90
42WT
42Q61L
par-3 (RNAi)
I
Normalized Cortical Intensity (PKC-3)
***
(39) (17) (19) (47)
wild type
pkc-3(ts)
cdc-42(wt)
cdc-42(Q61)
par-3(RNAi)
J
wild type
pkc-3(ts)
cdc-42(WT)
cdc-42(Q61L)
par-3(RNAi)
PKC-3 Normalised Intensity
Bkgd
Membrane
Cyto

Figure S5. Impact of CDC-42/GTP on membrane loading of anterior PARs, Related to Figure 3

(A) Midsection confocal images of zygotes showing that in *pkc-3(ts)* zygotes membrane localisation of PAR-6 and PKC-3, but not PAR-3, is dependent on CDC-42/GTP. *pkc-3(ts)* embryos are stained for PKC-3, PAR-6 and PAR-3. Representative images of control RNAi, top row, and defective phenotypes observed after RNAi of *cdc-42*, *cgef-1* or *par-3* are shown as indicated.

(B) Scatter plot of PKC-3, PAR-6 and PAR-3 anterior cortical intensities for datasets represented in (A). For each embryo (dot), the cortical intensity is divided by the corresponding PAR cytoplasmic intensity (mean±CI 95%). Values greater than 1 indicate presence at the cortex. See STAR Methods for details.

(C-E) Representative midsection confocal images (C), ASI quantifications (D), and normalized cortical intensity (E) for live wild-type, *pkc-3(ts)*, or *cgef-1(RNAi)* zygotes expressing the CDC-42/GTP-binding domain of WSP-1 (GBPwsp1), which monitors CDC-42 activity. White arrowheads highlight boundaries of spatial CDC-42 activity enrichment in wild type. Note that active CDC-42/GTP is localized uniformly to the membrane in PKC-3-inhibited zygotes (D) and shows similar levels to those present in wild-type (E). Therefore, CDC-42/GTP is in position to support PAR-6/PKC-3 anchoring at the membrane. Dataset for RNAi targeting *cgef-1* (CDC-42-GEF) is included for comparison. N (grey numbers) indicated in (D) and is the same for corresponding data in (E).

(F) Representative midsection confocal images of live *par-3(RNAi)* embryos expressing CDC-42(WT, mCherry) or CDC-42(Q61L, mCherry).

(G) Quantification of datasets represented in (F), which show no detectable rescue of GFP::PKC-3, despite observing significant enrichment of CDC-42(Q61L) at the membrane compared to wild-type CDC-42, suggesting we are stabilizing CDC-42 at the membrane. Note this analysis used heterozygous worms due to difficulties obtaining non-silenced homozygotes after crossing to a GFP::PKC-3 strain, which may account for the difference compared to (H-J). Intensity is normalized as in Figure 3D to *control(RNAi)* + CRT90 to facilitate comparison between the two figures.

(H) Representative midsection confocal images of *cdc-42(Q61L)* and *cdc-42(WT)* embryos co-stained for PAR-3 and PKC-3 after *par-3(RNAi)*.

(I) PKC-3 cortical intensity is normalized to cytoplasm and to wild type samples processed on the same day to account for variation between days (mean±CI 95%). Wild type (n=39), *pkc-3(ts)* (n=17), *cdc-42(WT)* (n=19) and *cdc-42(Q61L)* (n=47). Note that upon *par-3(RNAi)* in *cdc-42(Q61L)* embryos, we observe only a minor rescue of PKC-3 membrane localization compared to *pkc-3(ts)*. Thus, while increasing CDC-42/GTP levels can bias the system towards CDC-42-dependent PKC-3 assemblies (see Figure 5F-K), the bulk of PKC-3 membrane loading remains dependent on PAR-3, unlike in PKC-3-inhibited embryos.

(J) Comparison of membrane profiles of PKC-3 for the embryos in (I), highlighting membrane signal, showing mean ± SD. Briefly, normalized intensity profiles were extracted as 60 pixel stripes encompassing the embryo membrane, which was straightened using ImageJ. A 60x60px area in the region of peak PKC-3 membrane signal was projected in x to give a cross-section profile spanning background (Bkgd), crossing the membrane/cortex and into the cytoplasm (Cyto), which was then normalized such that background is set to 0 and cytoplasm to 1. Although *cdc-42(Q61L)* embryos show a minor peak of PKC-3 at the membrane, the magnitude of this peak in *cdc-42(Q61L)* embryos is significantly reduced compared to *pkc-3(ts)*. All analysis in (I-J) was limited to embryos with no detectable PAR-3 at the membrane.

**p<0.01, ***p<0.001, ****p<0.0001. Scale bars: 10μm.

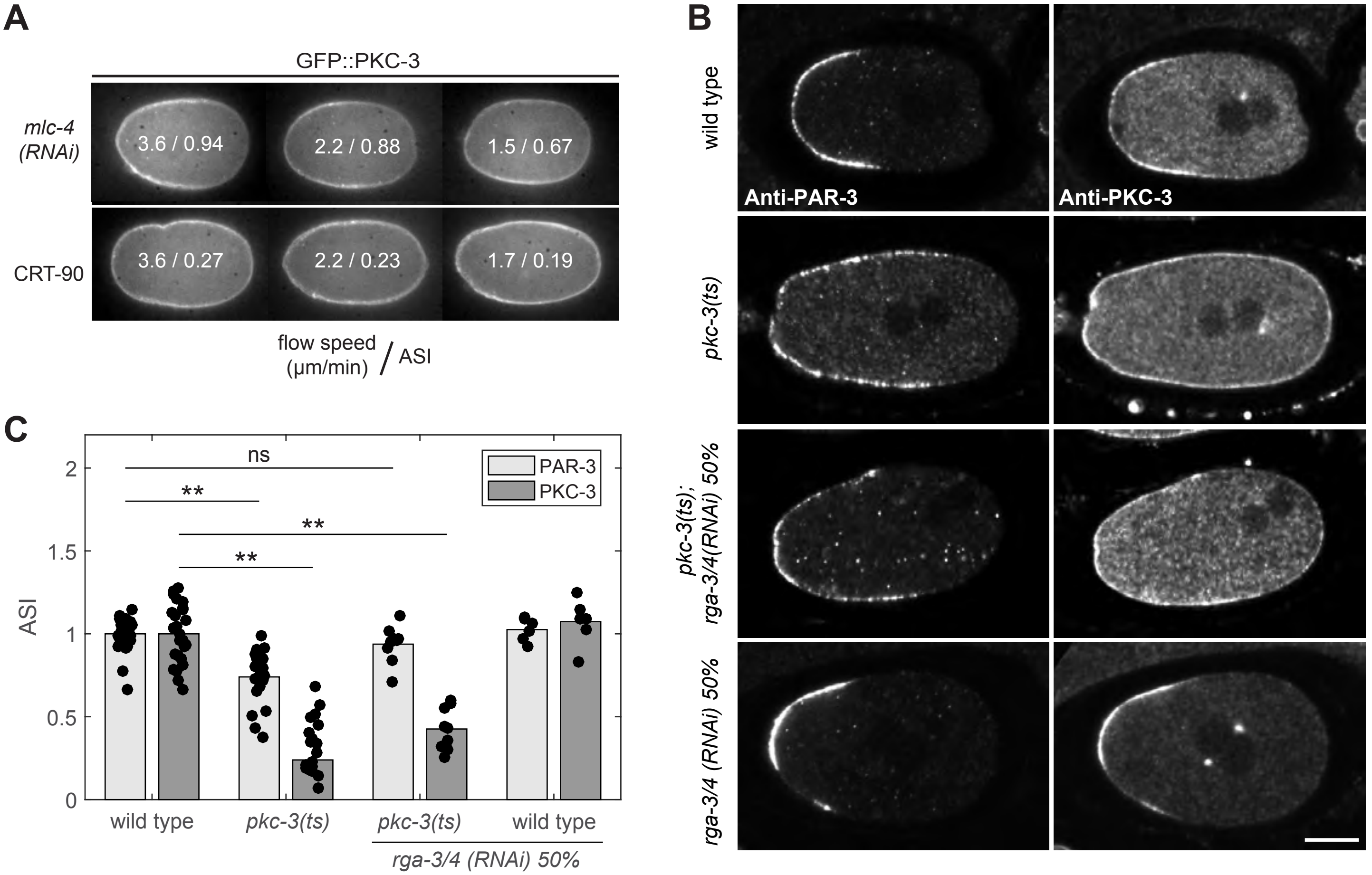

Figure S6. Analysis of the role of actomyosin flow and PAR segregation in wild type and PKC-3-inhibited embryos, Related to Figure 5

(A) Midsection confocal images of *mlc-4(RNAi)* and CRT90 treated embryos, matched by measured flow speed. Note all CRT90-treated embryos exhibit similar loss of asymmetry across all three flow rates, whereas *mlc-4(RNAi)* embryos retain asymmetry, though it decreases somewhat as flow rates are reduced. Flow rate (µm/min) / ASI are shown within each embryo.

(B) Representative midsection confocal images of maintenance phase wild-type or *pkc-3(ts)* embryos with or without partial RNAi-mediated depletion of the RhoGAPs RGA-3/4. Embryos were fixed and stained for both PAR-3 and PKC-3. Note that segregation of PAR-3 is fully rescued in *pkc-3(ts)* by *rga-3/4(RNAi)*, whereas PKC-3 remains present at the posterior. Scale bar: 10µm.

(C) ASI quantification of the full dataset represented in (B) reveals failure of *rga-3/4(RNAi)* to rescue PKC-3 asymmetry despite rescue of PAR-3 ASI to wild-type levels. ASI is shown normalized for each protein individually compared to N2. (N: wild type = 25, *pkc-3(ts)* = 22, *pkc-3(ts); rga-3/4(RNAi) 50% = 9,* wild type; *rga-3/4(RNAi)50% = 6)*

**p<0.01. Scale bars: 10µm.

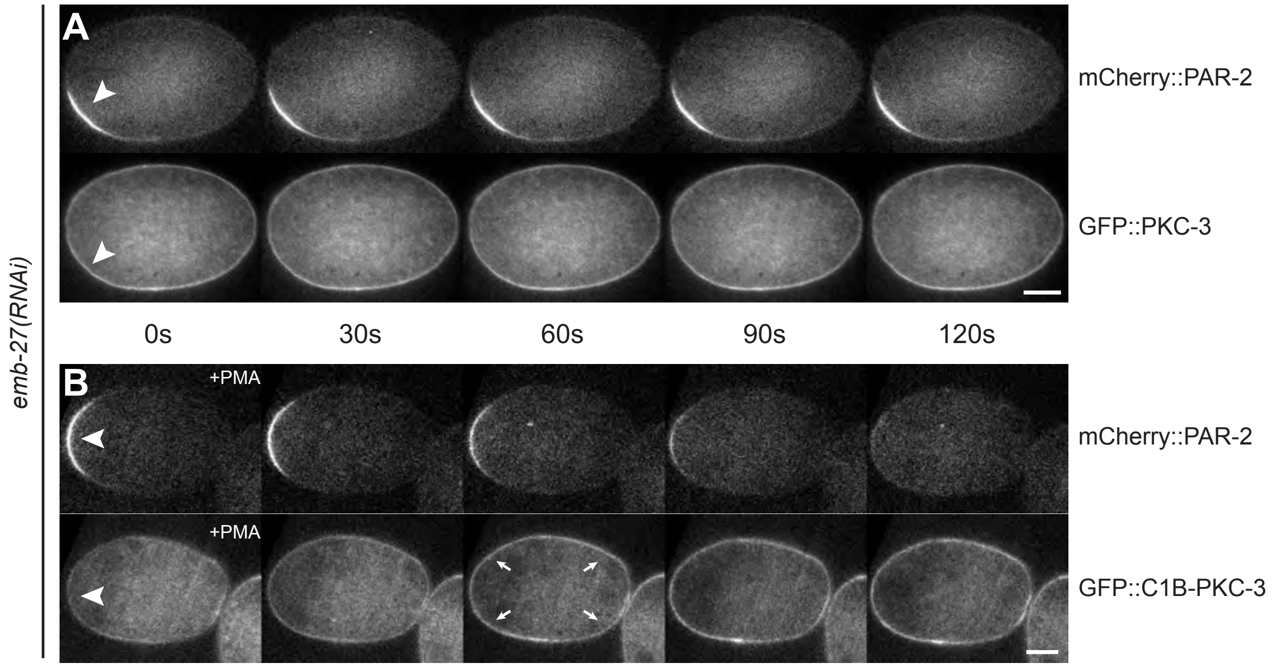

Figure S7. Anterior meiotic PAR-2 domains are rapidly cleared by targeting PKC-3 to the membrane, Related to Figure 6

(A) Midsection fluorescent images of *emb-27(RNAi)* zygotes expressing GFP::PKC-3 with mCherry::PAR-2. *emb-27(RNAi)* induces defects in meiotic progression, leading to inverted polarity with an anterior domain of PAR-2 near the meiotic spindle remnant, similar to *mei-1(RNAi)* (Wallenfang and Seydoux, 2000). This anterior meiotic domain remains on the membrane for more than 30 min (n=3).

(B) Same as (A), but zygotes express GFP::C1B-PKC-3, which is targeted to the membrane by the addition of PMA. (B). The increase in PKC-3 at the membrane rapidly clears the anterior PAR-2 domain upon PMA addition (3.2±1.7min, n=3). Arrows indicate enhanced PKC-3 membrane localisation 60 s after PMA addition.

Note that neither GFP::PKC-3 or C1B::GFP::PKC-3 are fully excluded by the PAR-2 domain (arrowheads). Scale bar: 10μm.
