# Supplementary figures and images for: aPKC Cycles between Functionally Distinct PAR Protein Assemblies to Drive Cell Polarity
Source: Dev Cell. 2017 Aug 21;42(4):400–415.e9. doi: 10.1016/j.devcel.2017.07.007 (PMC5563072; doi:10.1016/j.devcel.2017.07.007)

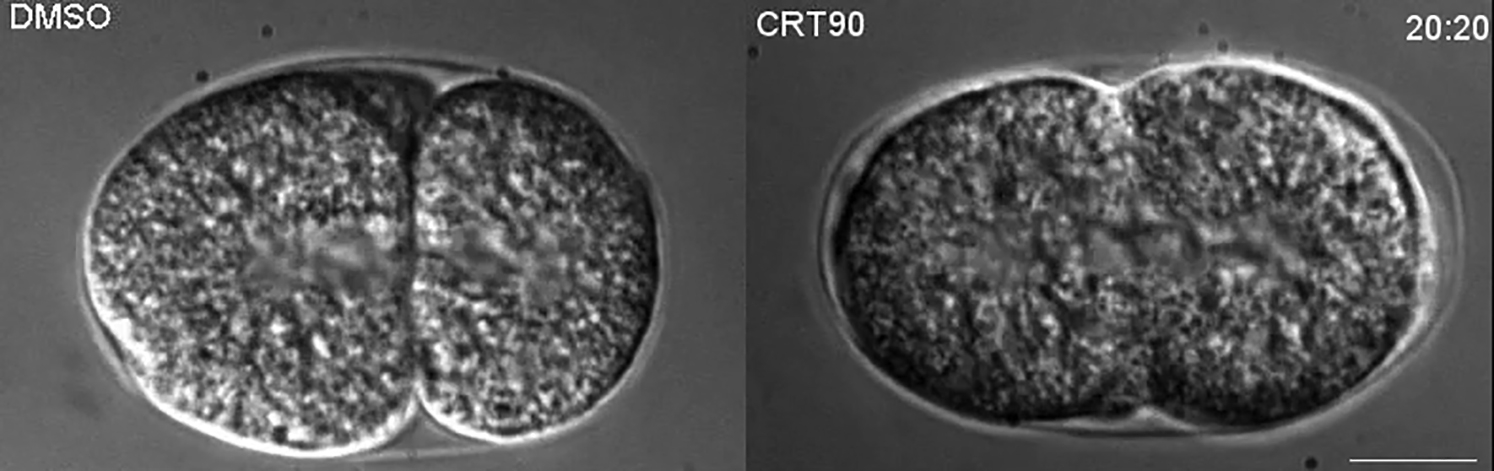

Supplement: Movie S1. Loss of PKC-3 Kinase Activity Leads to Symmetric Division in C. elegans Zygote, Related to Figure 1 — 0:00–0:05: CRT90-treated zygotes divide symmetrically. perm-1(RNAi) embryos are treated with DMSO (left) and CRT90 (10 μM, right) for ∼5 min prior to imaging. Acquisition of midplane DIC images starts at early establishment phase, and frames are captured every 20 s. Elapsed time, 21 min 20 s. Scale bar, 10 μm. 0:05–0:09: pkc-3(ts) zygotes divide symmetrically. Wild-type (left) and pkc-3(ts) (middle) zygotes are imaged at the restrictive temperature of 25°C. PKC-3-depleted pkc-3(RNAi) (right panel) is imaged at room temperature (∼19°C). Acquisition of midplane DIC images starts at early establishment phase, and frames are captured every 20 s. Elapsed time, 14 min 40 s. Scale bar, 10 μm. [file mmc2.jpg]

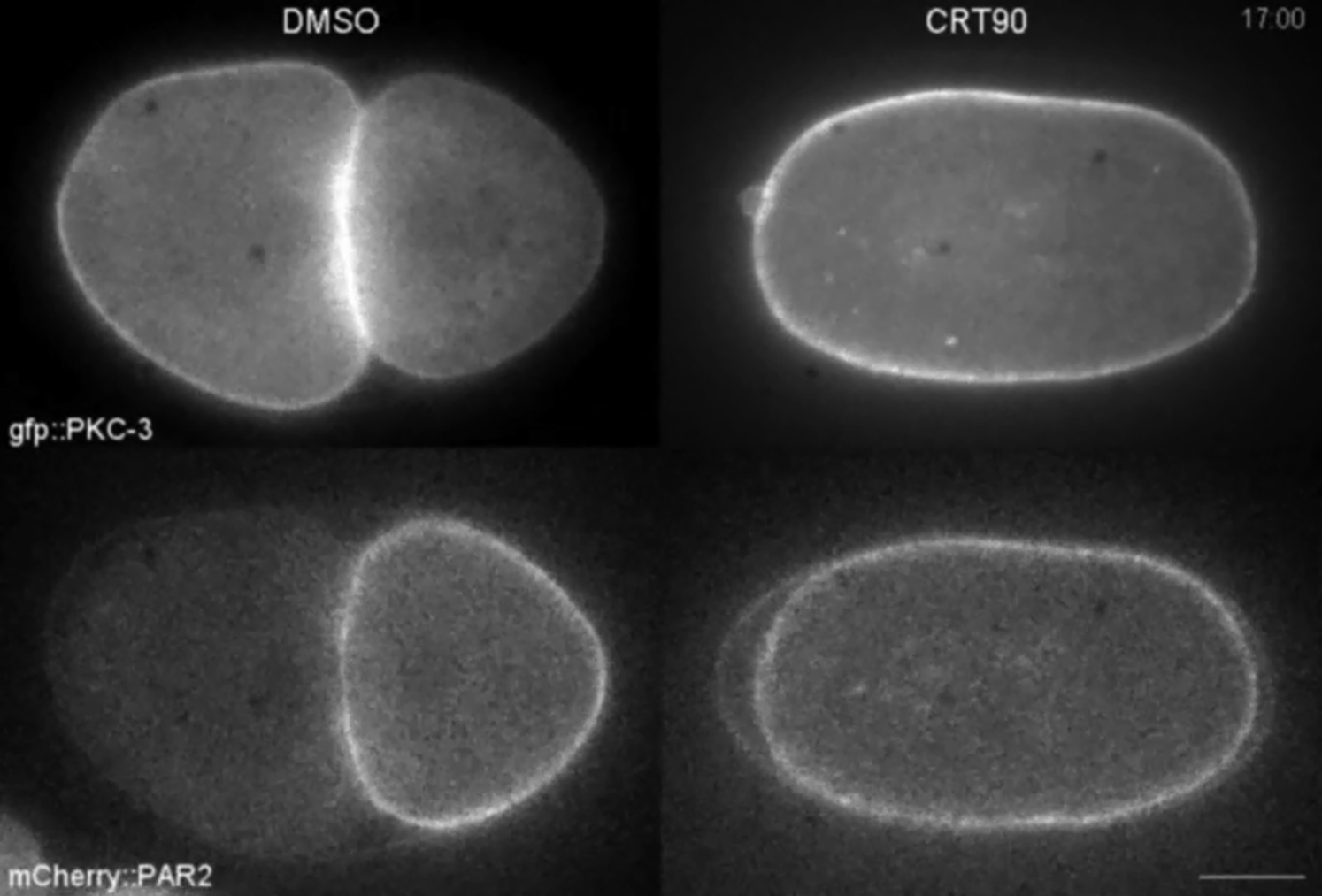

Supplement: Movie S2. Acute PKC-3 Inhibition Leads to Overlap of PAR-6/PKC-3 and PAR-2 in the C. elegans Zygote, Related to Figure 2 — perm-1(RNAi) embryos expressing either GFP::PKC-3 (0:00–0:07, elapsed time 23 min 00 s) or PAR-6::GFP (0:07–0:14, elapsed time 25 min 40 s) (top) and mCherry::PAR-2 (bottom) are treated with DMSO (left) or CRT-90 (10 μM, right) for ∼5 min prior to imaging. Acquisition of midplane fluorescent images begins at early establishment phase, and frames are captured every 20 s. Note that upon acute inhibition of PKC-3, PAR-6 and PKC-3 remain present at the membrane despite invasion of the anterior domain by PAR-2. Though both PAR-6 and PKC-3 are slightly asymmetric during the establishment phase, they become fully uniform, co-localized with PAR-2 throughout the embryo by maintenance phase. Scale bar, 10 μm. [file mmc3.jpg]

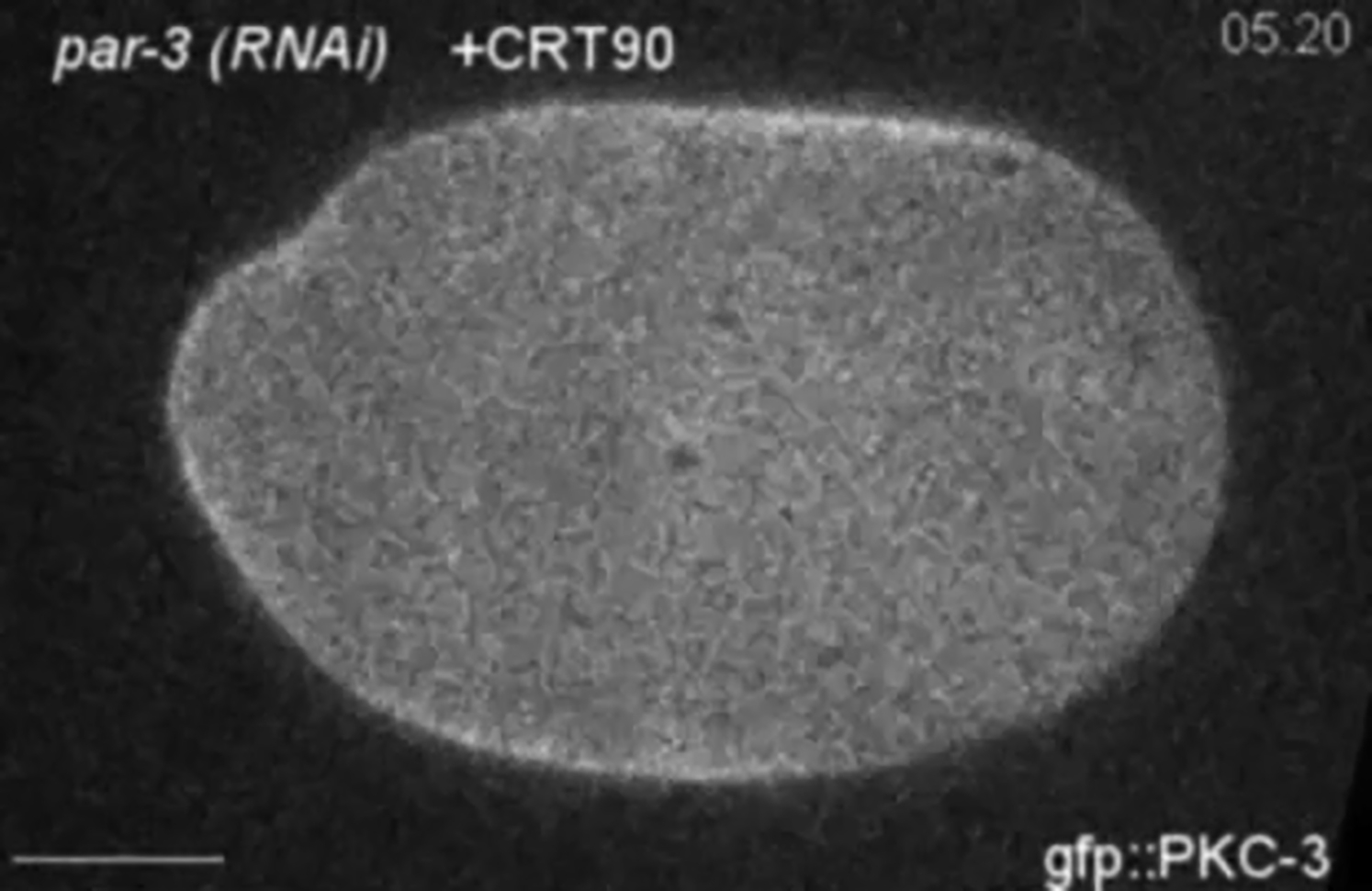

Supplement: Movie S3. PKC-3 Acute Inhibition Partially Rescues PKC-3 Membrane Loading in Absence of PAR-3, Related to Figure 3 — perm-1/par-3 (RNAi) embryo expressing GFP::PKC-3 is treated with CRT90 (10 μM) during image acquisition to illustrate the timescale of rescue. PKC-3 is visible within minutes of CRT90 treatment. Acquisition of midplane fluorescent images begins at early establishment phase, and frames are captured every 20 s. Elapsed time, 20 min. Scale bar, 10 μm. [file mmc4.jpg]

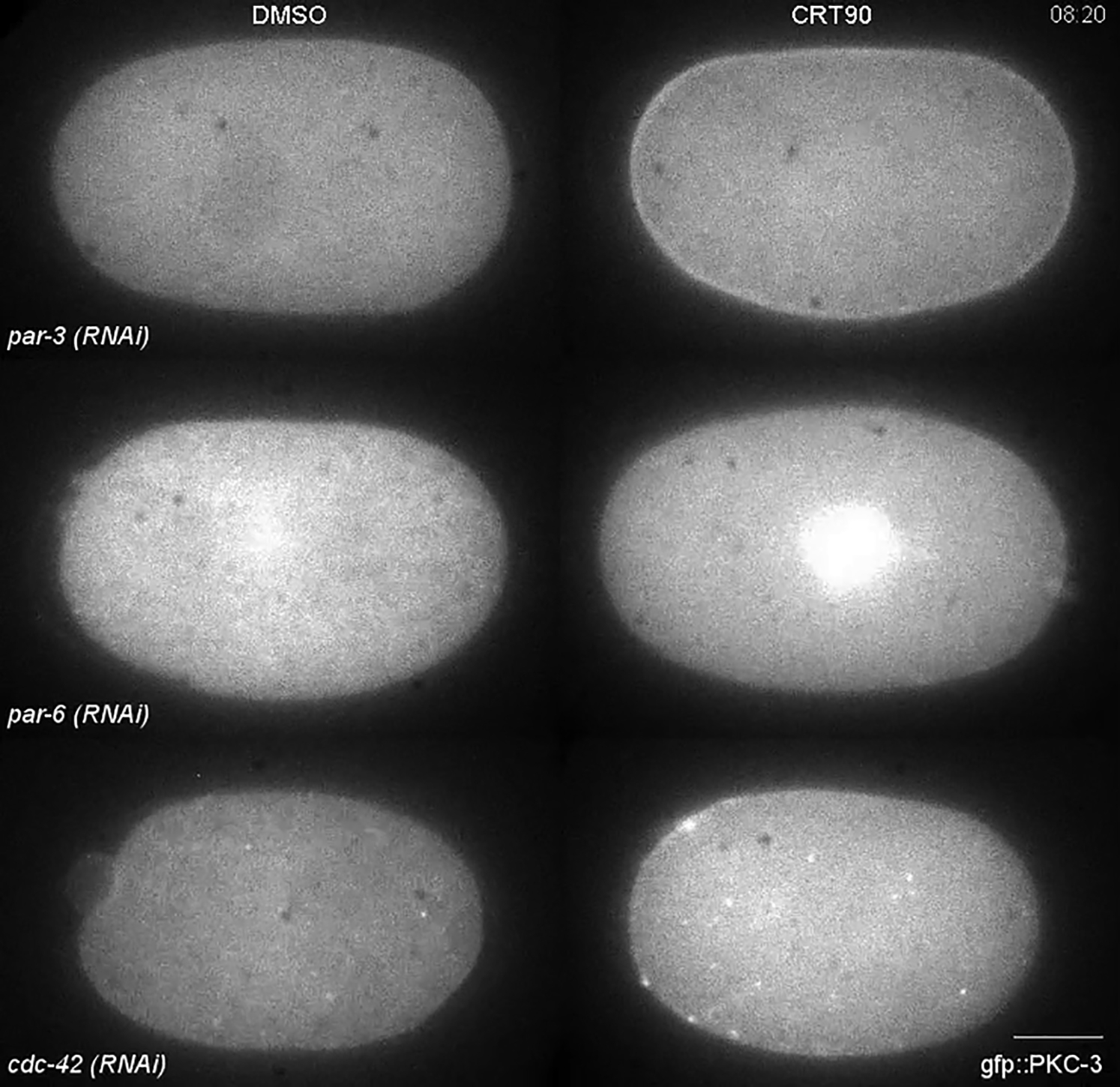

Supplement: Movie S4. Acute PKC-3 Inhibition Can Rescue PKC-3 Membrane Loading in the Absence of PAR-3, but Not CDC-42 and PAR-6, Related to Figure 3 — perm-1/par-3 (RNAi) (top), perm-1/par-6 (RNAi) (middle), and perm-1/cdc-42 (RNAi) (bottom) embryos expressing GFP::PKC-3 are treated with DMSO (left) and CRT90 (10 μM, right) for ∼5 min prior to imaging. Note that PKC-3 localization at the membrane is lost, from establishment phase in par-6 (RNAi) and from maintenance phase in cdc-42 (RNAi), neither of which is rescued by CRT90. Acquisition of midplane fluorescent images begins at early establishment phase, and frames are captured every 20 s. Elapsed time, 16 min. Scale bar, 10 μm. [file mmc5.jpg]

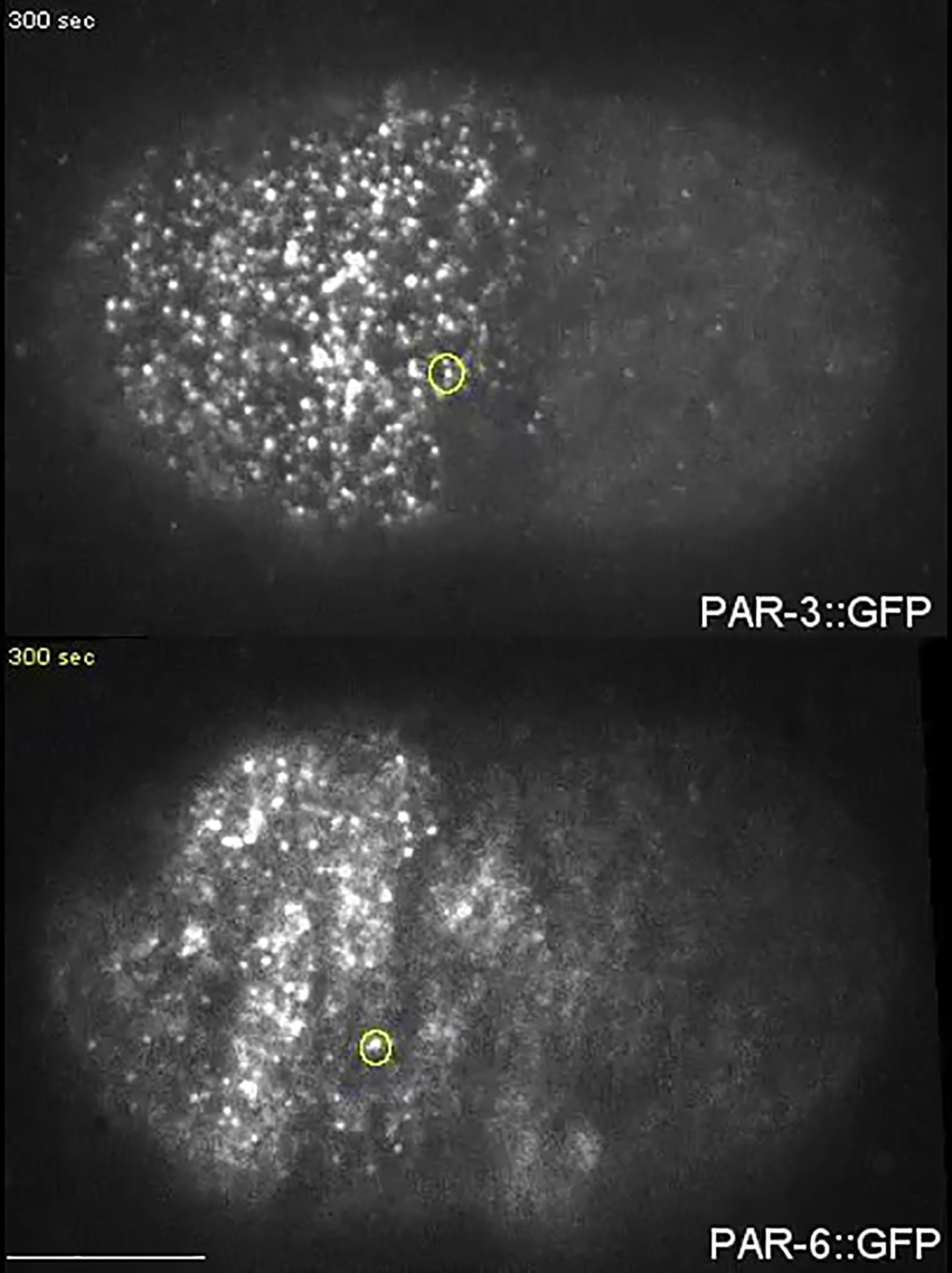

Supplement: Movie S5. Polarization Is Associated with aPAR Clusters, Related to Figure 4 — 0:00–0:04: PAR-3 and PAR-6 form clustered assemblies that are efficiently transported by anterior-directed cortical flows. Wild-type embryos expressing PAR-3::GFP (top) or PAR-6::GFP (bottom) are imaged by pseudo-TIRF microscopy every 5 s. Acquisition of cortical fluorescent images begins at the onset of anterior-directed cortical flow. Note robust advection of PAR-3 and PAR-6 clusters (yellow circle, and surrounding clusters). Also, unlike PAR-3, there appears to be a diffuse PAR-6 population, which lags behind PAR-6 clusters. Elapsed time, 7 min. Scale bar, 10 μm. 0:04–0:08: PAR-3 CR1 oligomerization domain is required for efficient transport by cortical flows. Embryos express either PAR-3(WT)::GFP (top) or PAR-3ΔCR1::GFP (bottom) together with PH-GBP, which tethers them to the membrane. Embryos are imaged by pseudo-TIRF microscopy every 5 s. Acquisition of cortical fluorescent images begins at the onset of anterior-directed cortical flow. Note that whereas PAR-3(WT) proteins are segregated to the anterior (top), oligomerization-deficient PAR-3 (PAR-3ΔCR1), even tethered to the membrane, is not, remaining mostly uniform at the cortex throughout the division. Elapsed time, 25 min 00 s. Scale bar, 10 μm. [file mmc6.jpg]

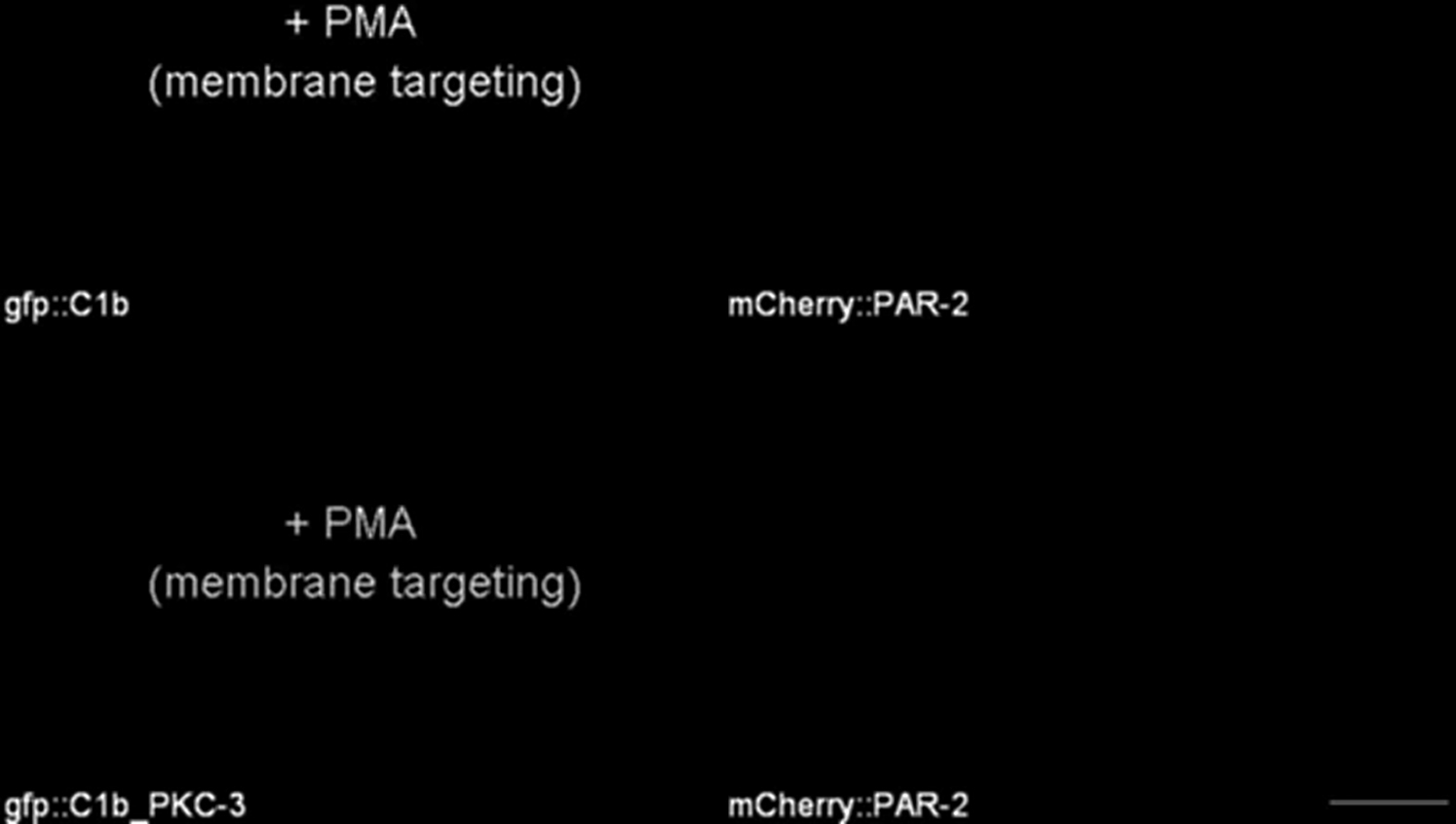

Supplement: Movie S6. PKC-3 Membrane-Targeting Assay for Assessing PKC-3 Activity In Vivo, Related to Figure 6 — Wild-type embryos expressing mCherry::PAR-2 (right) and either GFP::C1B (top left) or GFP::C1B::PKC-3 (bottom left). C1B constructs are targeted to the membrane uniformly upon addition of PMA (100 μM, left). Whereas targeting C1B alone to the membrane has little effect on PAR-2 domain size (top right), uniform membrane localization of C1B::PKC-3 leads to significant shrinkage of the PAR-2 domain (bottom right). Note that PAR-2 is not fully removed compared with par-3(RNAi) in Movie S7. Acquisition of midplane fluorescent images begins at late establishment phase (roughly when pronuclei meet), and frames are captured every 15 s. Elapsed time, 9 min 00 s. Scale bar, 10 μm. [file mmc7.jpg]

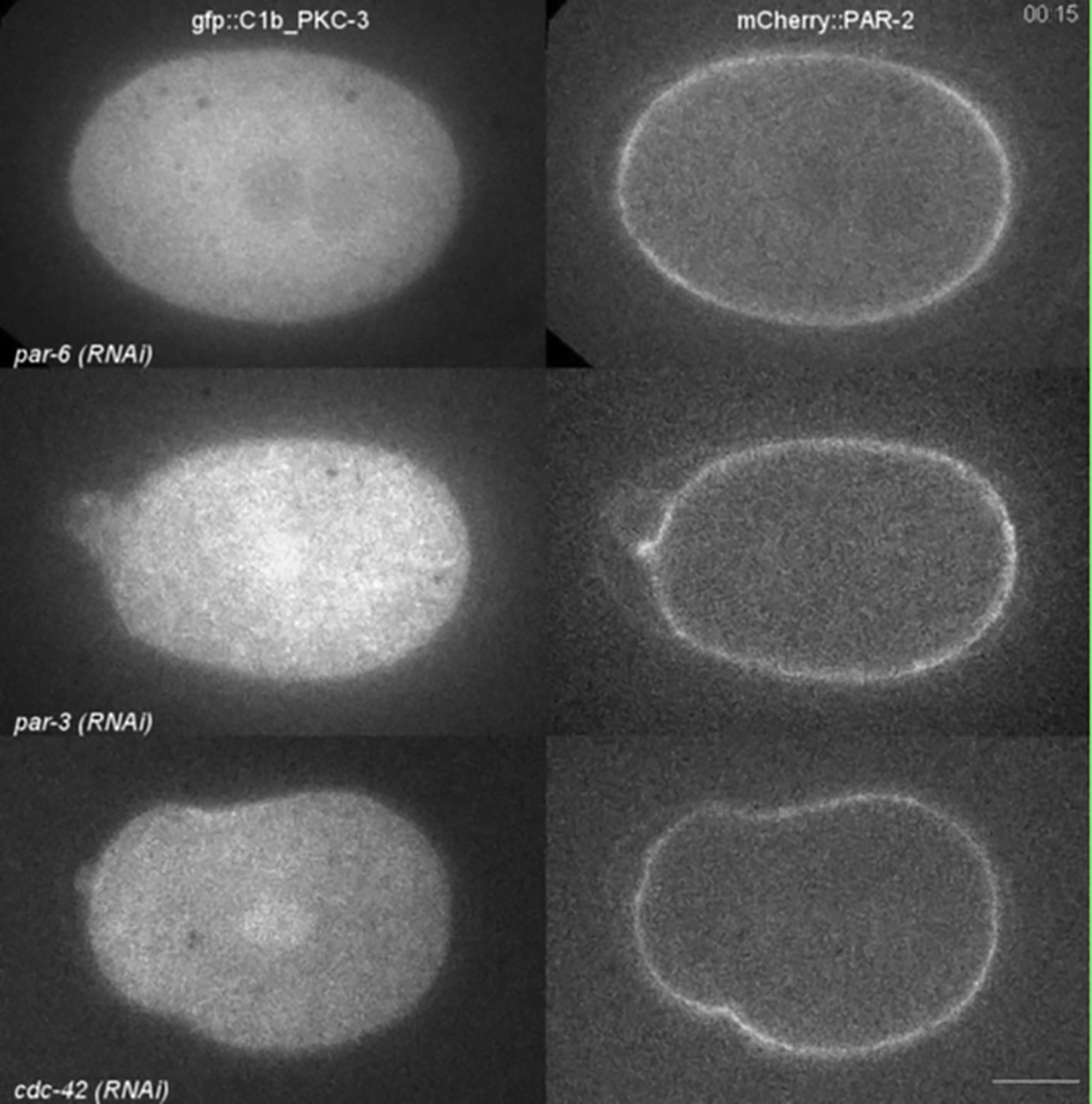

Supplement: Movie S7. PKC-3 Membrane-Targeting Assay Reveals Opposing Roles for PAR-3 and CDC-42 in Regulating PKC-3 Activity, Related to Figure 6 — Embryos expressing mCherry::PAR-2 (right) and GFP::C1B::PKC-3 (left) are depleted of PAR-6 (par-6 (RNAi), top), PAR-3 (par-3 (RNAi), middle), and CDC-42 (cdc-42 (RNAi), bottom). Upon PMA (100 μM) addition, C1B::PKC-3 is targeted uniformly to the membrane. Whereas this does not affect PAR-2 uniform distribution at the membrane in par-6 (RNAi) and cdc-42 (RNAi), it triggers a complete and rapid removal of PAR-2 in the absence of PAR-3 (bottom right). Acquisition of midplane fluorescent images begins at late establishment phase (roughly when pronuclei meet), and frames are captured every 15 s. Elapsed time, 8 min 45 s. Scale bar, 10 μm. [file mmc8.jpg]
