# Supplementary material for: aPKC Cycles between Functionally Distinct PAR Protein Assemblies to Drive Cell Polarity
Source: Dev Cell. 2017 Aug 21;42(4):400–415.e9. doi: 10.1016/j.devcel.2017.07.007 (PMC5563072; doi:10.1016/j.devcel.2017.07.007)
Supplement: Document S2. Article plus Supplemental Information [file mmc9.pdf]

Article

Developmental Cell

aPKC Cycles between Functionally Distinct PAR Protein Assemblies to Drive Cell Polarity

Graphical Abstract

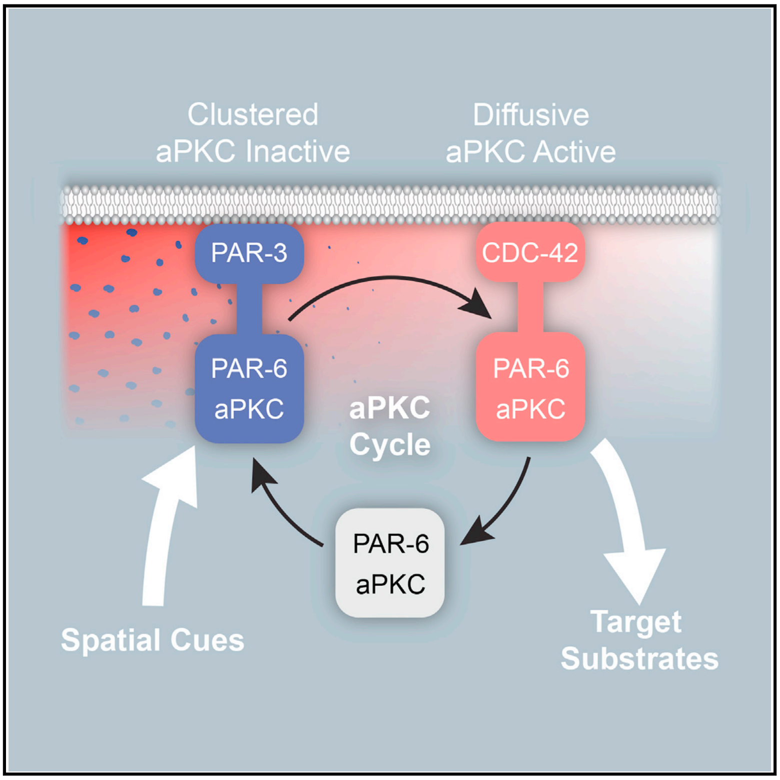

Authors

Josana Rodriguez, Florent Peglion, Jack Martin, ..., Daniel St Johnston, Julie Ahringer, Nathan W. Goehring

Correspondence

josana.rodriguez@ncl.ac.uk (J.R.),
nate.goehring@crick.ac.uk (N.W.G.)

In Brief

PAR polarity pathway-mediated cell polarization relies on a conserved network of proteins including PAR-3, CDC-42, PAR-6, and aPKC. Rodriguez, Peglion et al. uncover a division of labor whereby PAR-6 and aPKC cycle between distinct cue-sensing and effector assemblies that act cooperatively to polarize the one-cell *C. elegans* zygote.

Highlights

- Distinct aPKC-containing assemblies respond to cues and generate polarity signals
- Clustering of aPAR proteins on the membrane enables segregation by cortical flow
- PAR-3 promotes both loading and polarization of aPKC but limits its activity *in vivo*
- aPKC activity links cue-sensing and effector assemblies to drive efficient polarization

Rodriguez et al., 2017, Developmental Cell *42*, 400–415
August 21, 2017 © 2017 The Author(s). Published by Elsevier Inc.
http://dx.doi.org/10.1016/j.devcel.2017.07.007

Developmental Cell
Article

aPKC Cycles between Functionally Distinct PAR Protein Assemblies to Drive Cell Polarity

Josana Rodriguez,[1,2,6,7,*] Florent Peglion,[3,6] Jack Martin,[1] Lars Hubatsch,[3] Jacob Reich,[3] Nisha Hirani,[3] Alicia G. Gubieda,[1] Jon Roffey,[4] Artur Ribeiro Fernandes,[2] Daniel St Johnston,[2] Julie Ahringer,[2] and Nathan W. Goehring[3,5,*]
[1]Institute for Cell and Molecular Biosciences, Newcastle University, Newcastle upon Tyne NE2 4HH, UK
[2]Wellcome Trust/Cancer Research UK Gurdon Institute, Cambridge CB2 1QN, UK
[3]The Francis Crick Institute, London NW1 1AT, UK
[4]Cancer Research Technology, Wolfson Institute for Biomedical Research, London WC1E 6BT, UK
[5]Medical Research Council Laboratory for Molecular Cell Biology, University College London, London WC1E 6BT, UK
[6]These authors contributed equally
[7]Lead Contact
*Correspondence: josana.rodriguez@ncl.ac.uk (J.R.), nate.goehring@crick.ac.uk (N.W.G.)
http://dx.doi.org/10.1016/j.devcel.2017.07.007

SUMMARY

The conserved polarity effector proteins PAR-3, PAR-6, CDC-42, and atypical protein kinase C (aPKC) form a core unit of the PAR protein network, which plays a central role in polarizing a broad range of animal cell types. To functionally polarize cells, these proteins must activate aPKC within a spatially defined membrane domain on one side of the cell in response to symmetry-breaking cues. Using the *Caenorhabditis elegans* zygote as a model, we find that the localization and activation of aPKC involve distinct, specialized aPKC-containing assemblies: a PAR-3-dependent assembly that responds to polarity cues and promotes efficient segregation of aPKC toward the anterior but holds aPKC in an inactive state, and a CDC-42-dependent assembly in which aPKC is active but poorly segregated. Cycling of aPKC between these distinct functional assemblies, which appears to depend on aPKC activity, effectively links cue-sensing and effector roles within the PAR network to ensure robust establishment of polarity.

INTRODUCTION

A crucial step in the polarization of metazoan cells is the localization of conserved sets of polarity effectors, known as the partitioning-defective or PAR proteins, to discrete membrane-associated cortical domains. Regulation of PAR protein distribution is essential for the localized activation of signaling pathways that coordinate many aspects of embryonic development, including asymmetric cell division, epithelial organization, and embryo axis establishment (Goldstein and Macara, 2007; St Johnston and Ahringer, 2010). Although the precise details vary between systems, in most cases the conserved PDZ domain proteins PAR-3 and PAR-6, the small guanosine triphosphatase (GTPase) CDC-42 and atypical protein kinase C (aPKC) act together to establish polarity on one side of the cell and drive asymmetry of a range of downstream pathways (reviewed in Goehring, 2014; McCaffrey and Macara, 2012; Suzuki et al., 2004; Ziomek et al., 1982).

In *Caenorhabditis elegans*, PAR-3, PAR-6, CDC-42, and the aPKC ortholog, PKC-3, play an essential role in polarizing the one-cell embryo or zygote by defining an anterior domain and hence are referred to as anterior PARs or aPARs (Figures 1A–1C). An opposing set of posterior PARs or pPARs, consisting of PAR-1, PAR-2, LGL-1, and the CDC-42 GAP, CHIN-1, form a complementary domain at the posterior. Together, aPARs and pPARs define the anterior-posterior axis of the zygote and orchestrate an asymmetric division that restricts germline determinants to the posterior daughter cell (Beatty et al., 2010; Boyd et al., 1996; Etemad-Moghadam et al., 1995; Gotta et al., 2001; Guo and Kemphues, 1995; Hoege et al., 2010; Kay and Hunter, 2001; Kumfer et al., 2010; Tabuse et al., 1998; Watts et al., 1996).

Polarization is triggered by the sperm-donated centrosome via two semi-redundant pathways (Figures 1A and 1B). First, the centrosome induces actomyosin cortical flow away from the newly defined posterior pole, which transports membrane-associated aPAR proteins into the anterior (Cheeks et al., 2004; Goehring et al., 2011b; Mayer et al., 2010; Munro et al., 2004). Second, centrosomal microtubules promote local loading of PAR-2 in the posterior. PAR-2 then recruits PAR-1, which drives posterior exclusion of aPARs through phosphorylation of PAR-3 (Boyd et al., 1996; Hao et al., 2006; Motegi et al., 2011). Following this "establishment phase," the zygote enters a "maintenance phase" during which mutual antagonism between anterior and posterior PARs ensures their continued asymmetric localizations (Boyd et al., 1996; Cuenca et al., 2003; Etemad-Moghadam et al., 1995; Guo and Kemphues, 1995; Tabuse et al., 1998; Watts et al., 1996).

Anterior PAR protein function is mediated through the kinase activity of PKC-3, which can phosphorylate PAR-1, PAR-2, and LGL-1 and drive their dissociation from the membrane (Beatty et al., 2010; Hao et al., 2006; Hoege et al., 2010; Hurov et al., 2004; Motegi et al., 2011). PAR-3, PAR-6, and CDC-42 are all required for proper PKC-3 membrane localization (Gotta et al.,

 © 2017 The Author(s). Published by Elsevier Inc.
This is an open access article under the CC BY license (http://creativecommons.org/licenses/by/4.0/).

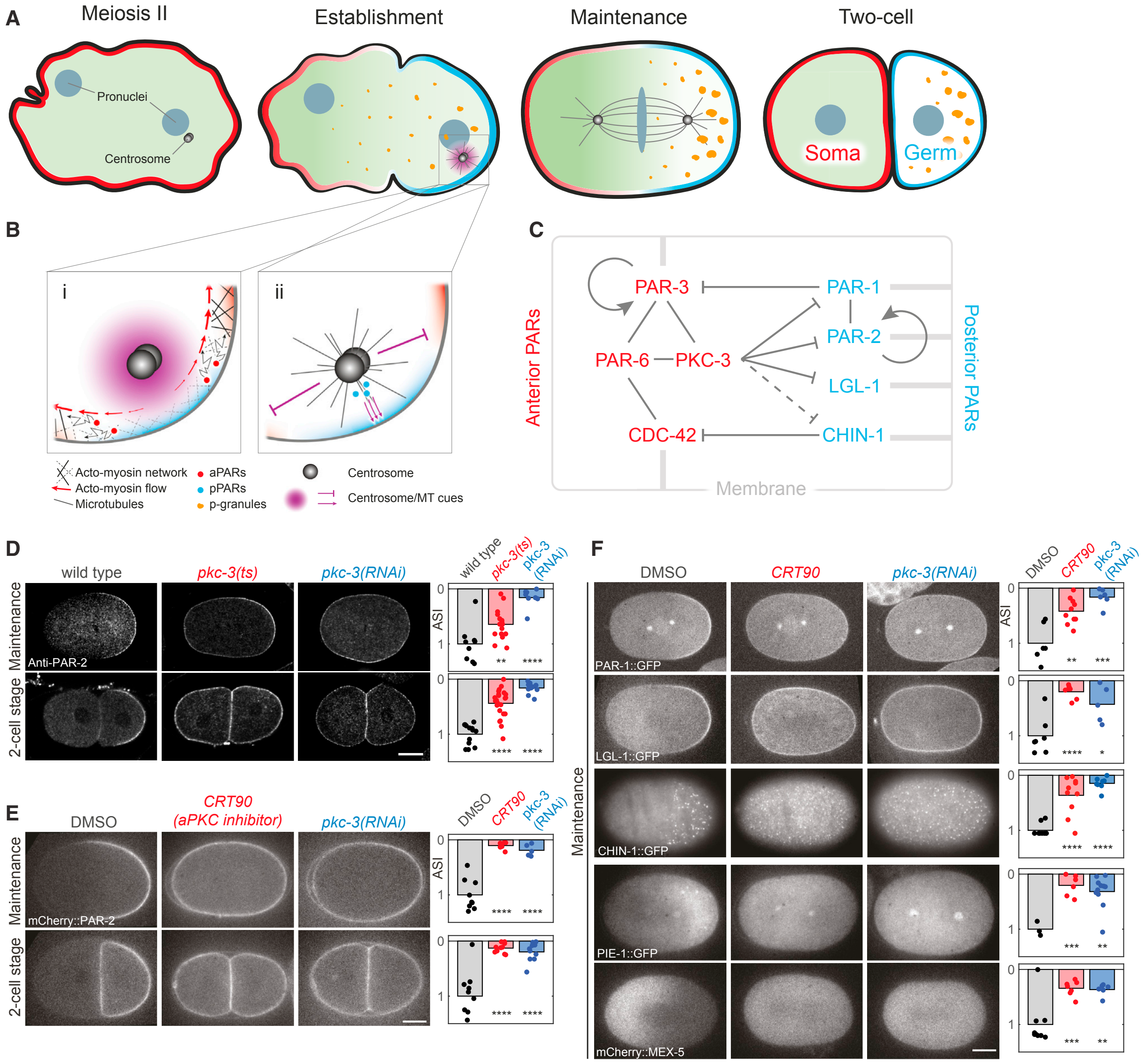

Figure 1. PKC-3 Kinase Inhibition Leads to Symmetric Division and Loss of Asymmetry of Downstream Polarity Markers

(A–C) Model for symmetry breaking by the PAR system in *C. elegans*. aPARs (red) initially occupy the membrane and pPARs (blue) are cytoplasmic (A, Meiosis II). A cue (purple) from the centrosome pair (black spheres) segregates aPARs into the anterior and promotes formation of a posterior PAR domain at the opposite pole (A, Establishment). PAR domains are then stable until cytokinesis (A, Maintenance) and drive polarization of cytoplasmic factors such as MEX-5/6 (green) and P granules (orange), which ensure the daughter cells acquire distinct fates (A, Two-cell). (B) Symmetry breaking can occur in two ways: (i) segregation of aPARs by cortical actomyosin flow (advection); and (ii) posterior PAR-2 loading. (C) A complex network of physical and regulatory interactions links the PAR proteins. Membrane binding (gray lines), physical interactions (black lines), as well as positive (→) and negative (⊥) feedback, are shown. Where links are indirect or unknown, dashed lines are used. Both CDC-42 and PAR-3 are required for stable membrane association of PAR-6/PKC-3. PAR-6 and PKC-3 depend on each other for membrane association. PAR-2, LGL-1, and presumably CHIN-1, are able to load onto the membrane independently. PAR-1 also binds membrane but requires PAR-2 to reach maximal concentrations. PKC-3 phosphorylates PAR-1, PAR-2, and LGL-1 and displaces them from the membrane. Exclusion of CHIN-1 from the anterior is dependent on PKC-3, but whether it is a direct target of PKC-3 is unknown. Together, PAR-1, via phosphorylation of PAR-3, and CHIN-1, by suppressing activated CDC-42, prevent invasion of the posterior domain by aPARs. PAR-3 and PAR-2 have been proposed to undergo oligomerization, which is thought to enhance their membrane association (noted by circular arrows). See recent reviews (Goehring, 2014; Hoege and Hyman, 2013; Motegi and Seydoux, 2013) for more information.

(D) Midsection confocal images of fixed zygotes stained for PAR-2 at polarity maintenance and two-cell stage comparing wild-type, *pkc-3*(*ts*), and *pkc-3*(*RNAi*) conditions.

(E) Midsection fluorescent images of mCherry:PAR-2-expressing zygotes at maintenance and two-cell stage in DMSO (control), CRT90-treated, and *pkc-3*(*RNAi*).

(legend continued on next page)

2001; Kay and Hunter, 2001; Schonegg and Hyman, 2006; Tabuse et al., 1998). Consequently, loss of any of these four proteins results in identical zygote polarity phenotypes: posterior PAR proteins are found uniformly on the embryo membrane and the first cell division is symmetric, leading to cell fate defects and embryo lethality (Etemad-Moghadam et al., 1995; Kay and Hunter, 2001; Tabuse et al., 1998; Watts et al., 1996).

This similarity of aPAR mutant phenotypes, their co-segregation within the anterior domain, and their ability to interact with one another in a wide range of systems (Izumi et al., 1998; Joberty et al., 2000; Lin et al., 2000; Qiu et al., 2000) has led to consideration of an effective aPAR unit comprising PAR-3, PAR-6, PKC-3, and CDC-42. However, work across a range of cell types suggests that such minimalism belies significant complexity in the regulation of aPAR localization and function, which we are only beginning to decipher.

For example, in epithelia, PAR-3 and aPKC localize to distinct regions of the apical domain: PAR-3 is primarily junctional, while PAR-6 and aPKC are more apical and, together with CDC-42 and Crumbs, exclude PAR-1 and LGL from the apical domain (Betschinger et al., 2003; Harris and Peifer, 2005; Morais-De-Sa et al., 2010; Yamanaka et al., 2006; Yamanaka et al., 2003).

In the *C. elegans* zygote, two modes of aPAR membrane association have been proposed: one associated with PAR-3 and independent of CDC-42, and one dependent on CDC-42 but not associated with PAR-3. Supporting this hypothesis, PAR-6 and PKC-3 only partially co-localize with PAR-3 in wild-type embryos, but co-localize strongly when CDC-42 is depleted (Beers and Kemphues, 2006; Hung and Kemphues, 1999; Tabuse et al., 1998). However, it remains unclear whether these observations reflect the existence of discrete functional modules and, if so, what their respective functions are.

A primary role of the aPAR network is to restrict PKC-3 kinase activity to the anterior domain. However, because localization, function, and regulation of PKC-3 are tightly coupled, parsing their individual contributions is difficult using traditional RNAi and knockout studies. Consequently, despite the central role of PKC-3 in polarity, we lack insight into how the individual contributions by PAR-3, PAR-6, CDC-42, and PKC-3 itself combine to ensure PKC-3 is activated only within the anterior domain. To address these questions, we require tools to independently modulate the localization and function of aPAR proteins.

Here we describe methods to independently manipulate PKC-3 activity and localization, which we use to investigate how PKC-3 kinase activity regulates organization of the aPAR network, and how PKC-3 activity is modulated by other network members. We find that localized PKC-3 kinase activity is linked to dynamic cycling of PAR-6/PKC-3 between two functionally distinct aPAR assemblies: (1) a PAR-3-dependent assembly that is associated with clusters and efficiently responds to polarizing cues, but in which PKC-3 activity is inhibited, and (2) a more diffuse CDC-42-dependent assembly that is less able to respond to polarizing cues but contains active PKC-3 and is responsible for posterior PAR protein exclusion. We propose that the dynamic exchange of PAR-6/PKC-3 between these two assemblies allows the PAR network to efficiently translate symmetry-breaking cues into an asymmetric homogeneous domain of PKC-3 activity.

RESULTS

Acute Inhibition of PKC-3 Function Leads to Loss of Asymmetric Division

We took two approaches to inhibit PKC-3 kinase activity. First, we examined a previously identified temperature-sensitive allele of *pkc-3*, *ne4246* (Fievet et al., 2012), which alters a conserved Asp residue (D386V) close to the active site. Strains carrying *pkc-3*(*ne4246*) are subsequently referred to as *pkc-3*(*ts*). Consistent with loss of PKC-3 function, in *pkc-3*(*ts*) zygotes at the restrictive temperature (25°C), PAR-2 is not restricted to the posterior membrane and is partitioned symmetrically into the two blastomeres at the first cell division (Figure 1D). Loss of asymmetry was quantified by the asymmetry index (ASI) (see STAR Methods), which measures the asymmetry of a feature, e.g., PAR-2 membrane intensity, relative to wild-type on a scale from zero (no asymmetry) to 1 (normal asymmetry) (Figure 1D and Movie S1). Results below and in Figure S1 indicate that loss of asymmetry in *pkc-3*(*ts*) zygotes is due to loss of PKC-3 activity rather than degradation.

In parallel, we examined PKC-3 inhibitors in permeable, *perm-1*(*RNAi*) embryos (Carvalho et al., 2011) to identify compounds that yielded a PKC-3 deficient polarity phenotype. One compound, CRT0103390 (CRT90), a derivative of CRT0066854 (Figures S2A–S2C) (Kjær et al., 2013; Dorsey et al., 2013) resulted in embryos that progressed normally through the cell cycle but showed loss of PAR-2 asymmetry and divided symmetrically (Figure 1E and Movie S1). CRT90 embryos exhibited other common phenotypes associated with *pkc-3*(*RNAi*) and *pkc-3*(*ts*), including simultaneous division of the two daughter cells and ectopic spindle rotation in the anterior daughter cell, leading to a chain-like arrangement of cells in the 4-cell embryo (data not shown). As expected for an inhibitor of PKC-3 activity, CRT90 treatment caused loss of asymmetry in other posterior PAR proteins (PAR-1, LGL-1, and CHIN-1) as well as the loss of cytoplasmic asymmetry in the cell fate determinants PIE-1 and MEX-5 (Figure 1F).

PKC-3 Inhibition Yields Distinct Phenotypes from PKC-3 Depletion

We next investigated the distributions of the anterior PAR proteins when PKC-3 activity was inhibited, and compared these to those observed when PKC-3 was depleted by RNAi. Normally, PAR-3, PAR-6, and PKC-3 are efficiently segregated into the anterior during the polarity establishment phase and remain asymmetric until cytokinesis (Figures 1A and 2A–2F) (Cuenca et al., 2003). When PKC-3 is depleted by RNAi of *pkc-3*, PAR-6 fails to localize to the membrane (Figure 2A) (Hung and

(F) Midsection (PAR-1, LGL-1, PIE-1, MEX-5) or cortical (CHIN-1) fluorescent images of maintenance-phase zygotes expressing markers to various downstream polarity markers in DMSO (control), CRT90-treated, and *pkc-3*(*RNAi*). Asymmetry in (D) to (F) is quantified by the asymmetry index, with one being normal asymmetry and zero, no asymmetry (ASI, normalized to DMSO/WT controls).

*$p < 0.05$, **$p < 0.01$, ***$p < 0.001$, ****$p < 0.0001$. Scale bars, 10 μm. See also Figures S1 and S2; Movie S1.

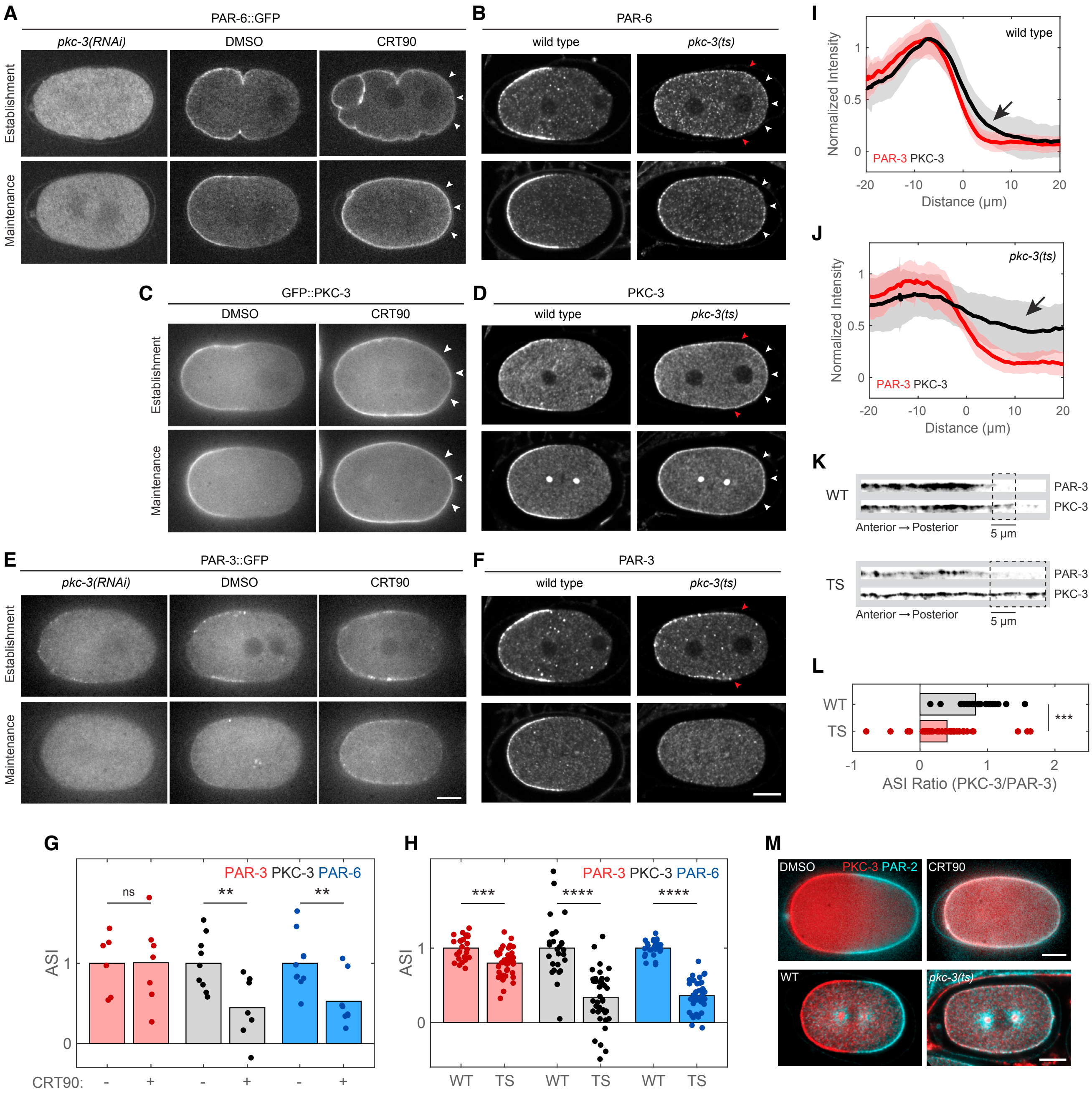

Figure 2. Membrane Localization of PAR-6/PKC-3 Is Decoupled from PAR-3 when PKC-3 Is Inactive

(A–F) Representative midsection confocal images of live and fixed zygotes at establishment and maintenance phase comparing control (DMSO, wild-type), *pkc-3*(*RNAi*), and PKC-3-inhibited (CRT90, *pkc-3*(*ts*)) conditions. PAR-6 (A and B) and PKC-3 (C and D) show loss of asymmetric membrane staining in PKC-3-inhibited zygotes at both establishment and maintenance phase (posterior localization indicated by white arrowheads). In *pkc-3*(*RNAi*), PAR-6 is absent from the membrane at all times. PAR-3 (E and F) still polarizes in PKC-3-inhibited zygotes, but becomes weaker and less asymmetric during maintenance phase. Note that (B), (D), and (F) show the same wild-type and TS zygotes with the PAR-3 boundary position in TS indicated (red arrowheads) to allow comparison: PAR-6 and PKC-3 are clearly visible at the posterior membrane (white arrowheads), while PAR-3 is undetectable, as in wild-type. Bright foci in (D) are non-specific centrosome staining.

(G and H) Normalized ASI measurements for late establishment phase datasets represented in (A) to (F). ASI is normalized to control (wild-type [WT] or DMSO) for each protein.

(I and J) Anterior to posterior membrane distributions of PAR-3 (red) and PKC-3 (black) in wild-type (I) and *pkc-3*(*ts*) (J) embryos. Arrows highlight the posterior extension of PKC-3 relative to PAR-3. Mean ± SD is shown.

(legend continued on next page)

Kemphues, 1999). By contrast, PAR-3 remains membrane associated and segregates into the anterior, but this population is generally reduced compared to wild-type and is lost as the cell proceeds through mitosis (Figure 2E) (Tabuse et al., 1998).

Unlike *pkc-3*(*RNAi*), when PKC-3 is inhibited by the D386V mutation or CRT90, PAR-6 and PKC-3 remain membrane associated, fail to segregate efficiently to the anterior, and a significant pool of both proteins remains localized at the posterior pole resulting in a loss of asymmetry relative to controls (Figures 2A–2D, 2G, and 2H; Movie S2). In contrast to PAR-6 and PKC-3, PAR-3 still segregates into the anterior during the establishment phase in PKC-3-inhibited zygotes (Figures 2E and 2F, Establishment). The domain is typically somewhat enlarged relative to wild-type zygotes, but PAR-3 asymmetry remains high and PAR-3 is absent from the posterior pole (Figures 2G and 2H). Following the establishment phase, PAR-3 levels at the membrane decline and become more symmetric (Figures 2E and 2F, Maintenance).

The distinct response of PAR-3 versus PAR-6 and PKC-3 is particularly clear in the quantification of dual-labeled fixed zygotes. In wild-type zygotes, the boundaries of the PAR-3 and PKC-3 domains are positioned similarly at the center of the zygote, although the PKC-3 domain extends a few microns further into the posterior (Figures 2I and 2K, WT). By contrast, in PKC-3-inhibited embryos, PKC-3 extends significantly further into the posterior compared with PAR-3, a condition we refer to as "decoupled" (Figures 2J–2L, TS). Consistent with this decoupling, upon PKC-3-inhibition we observe a decrease in co-localization between PAR-3 and PAR-6 at the membrane/cortex, even in the anterior domain where these PAR proteins overlap (Figure S3). Thus, we conclude that PKC-3 is required for the normally tight coupling between PAR-6/PKC-3 and PAR-3 during symmetry breaking.

These results point to an unappreciated complexity in the assembly and regulation of the PAR proteins at the cell membrane:

First, the loss of membrane-associated PAR-6 in PKC-3-depleted zygotes, but not in PKC-3-inhibited zygotes (Figure 2A), shows that disruption of PKC-3 activity or the resulting invasion of pPARs into the anterior (Figures 1D–1F) do not account for loss of PAR-6 membrane association. Rather, there appears to be a requirement for PKC-3 protein itself to target and stabilize PAR-6 at the membrane. Consistent with this interpretation, mutations in *par-2* and *par-1* fail to rescue PAR-6 membrane localization in *pkc-3*(*RNAi*) zygotes (Figure S4).

Second, in PKC-3-inhibited zygotes, anterior and posterior PAR protein distributions on the membrane overlap (Figure 2M). Posterior PAR proteins are thought to directly antagonize the ability of anterior PAR proteins to associate with the membrane, yet in these zygotes aPARs appear resistant to pPAR antagonism. PKC-3 inhibition could conceivably affect the activity of posterior PARs. However, we found that PAR-1 kinase activity, as measured by MEX-5 mobility (Griffin et al., 2011), appears normal in PKC-3-inhibited zygotes (Figures S2D–S2F). Thus, PKC-3 activity appears necessary to render anterior PARs sensitive to the antagonistic effects of posterior PARs, challenging the simple paradigm of mutual antagonism, which would predict pPAR dominance.

Finally, decoupling of PAR-6/PKC-3 from PAR-3 localization in PKC-3-inhibited zygotes during symmetry breaking suggests that PKC-3 drives formation of distinct PAR complexes or assemblies during polarity establishment in the *C. elegans* zygote. Contrary to what has been observed in *Drosophila* epithelia, where aPKC activity promotes decoupling of PAR-3 from PAR-6/aPKC and their targeting to distinct sites (Morais-De-Sa et al., 2010), here we observe the opposite: PKC-3 kinase activity is implicated in coupling the behaviors of PAR-3 and PAR-6/PKC-3, allowing their coordinated segregation during symmetry breaking.

PKC-3 Inhibition Promotes PAR-3-Independent Formation of CDC-42-Dependent PAR-6/PKC-3 Assemblies

If PKC-3 inhibition favors formation or trapping of a distinct functional assembly, we reasoned that it might affect the normal dependencies of PAR-6 and PKC-3 on PAR-3 and CDC-42. PKC-3 and PAR-6 normally require both PAR-3 and CDC-42 to localize stably to the membrane (Beers and Kemphues, 2006; Sailer et al., 2015). The dependency on PAR-3 is stronger: PKC-3 and PAR-6 fail to localize to the membrane in PAR-3-depleted zygotes (*par-3*(*RNAi*) in Figures 3A–3F) (Tabuse et al., 1998). By contrast, in CDC-42-depleted zygotes, PKC-3 and PAR-6 initially localize to the membrane and segregate to the anterior, but their membrane localization is gradually lost during the maintenance phase, becoming weaker and more uniform as zygotes approach cytokinesis (*cdc-42*(*RNAi*) Figures 3A–3F and Movie S4)(Beers and Kemphues, 2006; Gotta et al., 2001; Motegi and Sugimoto, 2006; Sailer et al., 2015; Schonegg and Hyman, 2006). Importantly, depletion of PAR-1 or PAR-2, which invade the anterior in the absence of PAR-3 or CDC-42 (Etemad-Moghadam et al., 1995; Gotta et al., 2001; Kay and Hunter, 2001; Schonegg and Hyman, 2006), fails to rescue PAR-6 membrane localization in these conditions (Figure S4). Thus, both PAR-3 and CDC-42 are directly required to promote membrane association of PAR-6 and PKC-3.

We find that under conditions of PKC-3 inhibition (D386V or CRT90), PKC-3 and PAR-6 no longer depend on PAR-3 to localize to the membrane (Figures 3A–3F and S5A; Movies S3 and S4). The degree of localization varies between the two methods of PKC-3 inhibition, possibly reflecting differences in the mechanism or timing/kinetics of kinase inhibition. By contrast, CDC-42 is still required for PKC-3 and PAR-6 membrane localization in PKC-3-inhibited zygotes (Figures 3A–3F, S5A, and S5B; Movie S4), indicating that CDC-42 is still required for PAR-6/PKC-3 membrane targeting even when PKC-3 is inhibited. Consistent with previous work showing that

(K) Close-up view of the boundary region showing PAR-3 (top) and PKC-3 (bottom) for one representative zygote for wild-type (WT) and *pkc-3*(*ts*) backgrounds as indicated. Dashed rectangular selection denotes regions where PKC-3 is present in absence of PAR-3.

(L) PKC-3 to PAR-3 ASI ratio for wild-type (WT) and *pkc-3*(*ts*).

(M) Dual labeling of PAR-2 and PKC-3 in live, CRT90-treated zygotes (top) and fixed, *pkc-3*(*ts*) embryos (bottom) reveal overlap of aPAR and pPAR proteins.

**$p < 0.01$, ***$p < 0.001$, ****$p < 0.0001$. ns, not significant. Scale bars, 10 μm. See also Figures S3 and S4; Movie S2.

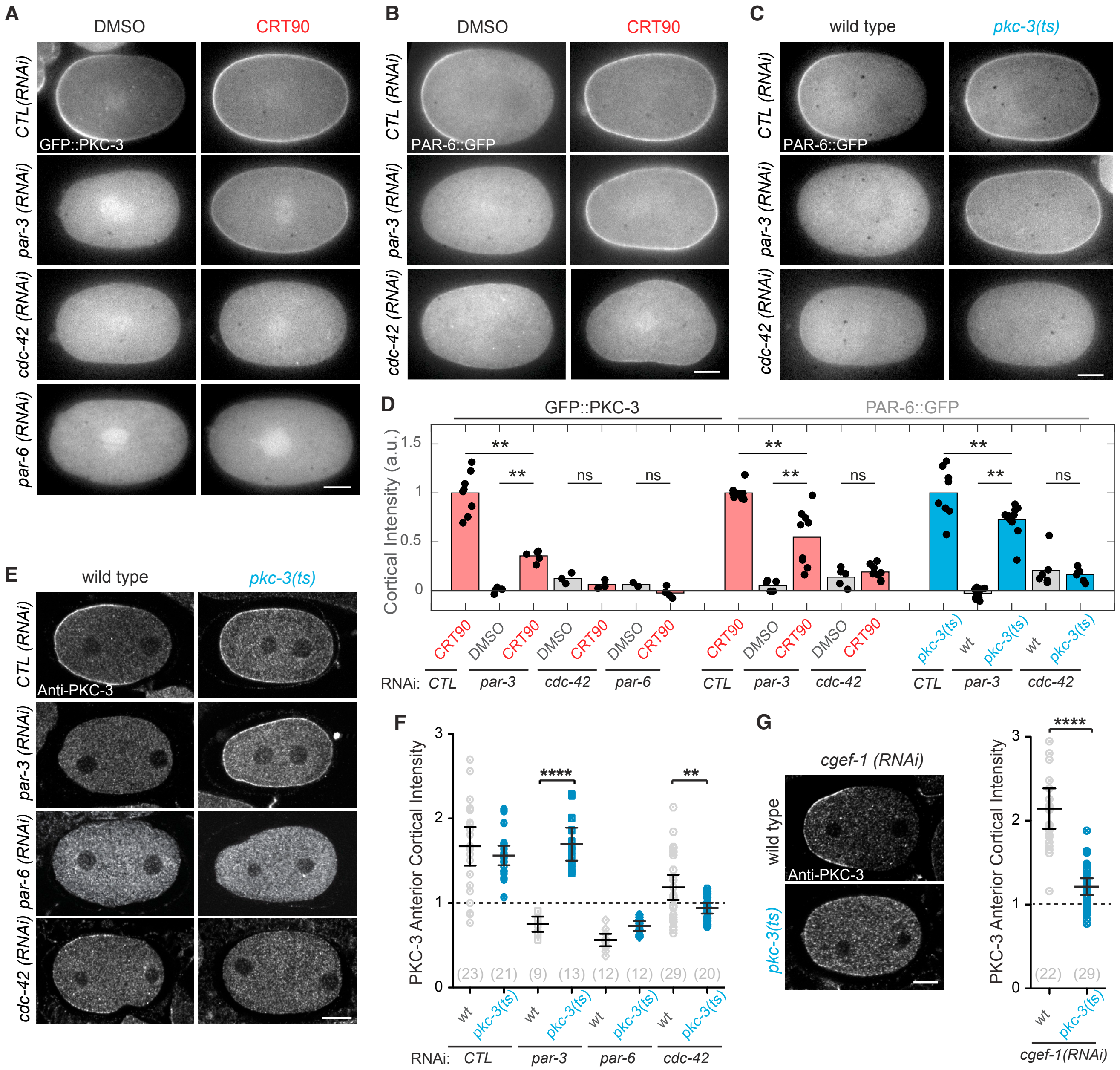

Figure 3. PKC-3 Inhibition Promotes PAR-3-Independent Formation of CDC-42-Dependent PAR-6/PKC-3 Assemblies

(A–C) Representative midsection confocal images of live embryos at maintenance phase showing GFP::PKC-3 (A) or PAR-6::GFP (B and C) of DMSO, CRT90-treated, and *pkc-3*(*ts*) zygotes subject to RNAi as indicated.

(D) Quantification of rescue for datasets represented in (A) to (C), normalized to membrane signal in control RNAi and CRT90-treated/*pkc-3*(*ts*) zygotes for each dataset.

(E) Representative midsection confocal images of wild-type and *pkc-3*(*ts*) zygotes during polarity establishment subject to RNAi as indicated and immunostained for PKC-3.

(F) Quantification of rescue as measured by anterior domain cortical intensity of PKC-3 for datasets represented in (E). For each zygote, anterior PKC-3 cortical intensity is divided by cytoplasmic intensity. Values greater than 1 indicate presence at the membrane. Mean ± 95% confidence interval (CI) (N) is shown. See STAR Methods for further details.

(G) Representative midsection confocal images during polarity establishment of wild-type and *pkc-3*(*ts*) embryos upon *cgef-1*(*RNAi*), stained for PKC-3. Scatterplot representing the anterior domain cortical intensity of PKC-3 as in (F) in *cgef-1*(*RNAi*) and *pkc-3*(*ts*);*cgef-1*(*RNAi*). Mean ± 95% CI (N) is shown.

$^{**}p < 0.01$, $^{****}p < 0.0001$. ns, not significant. Scale bars, 10 μm. See also Figure S5; Movies S3 and S4.

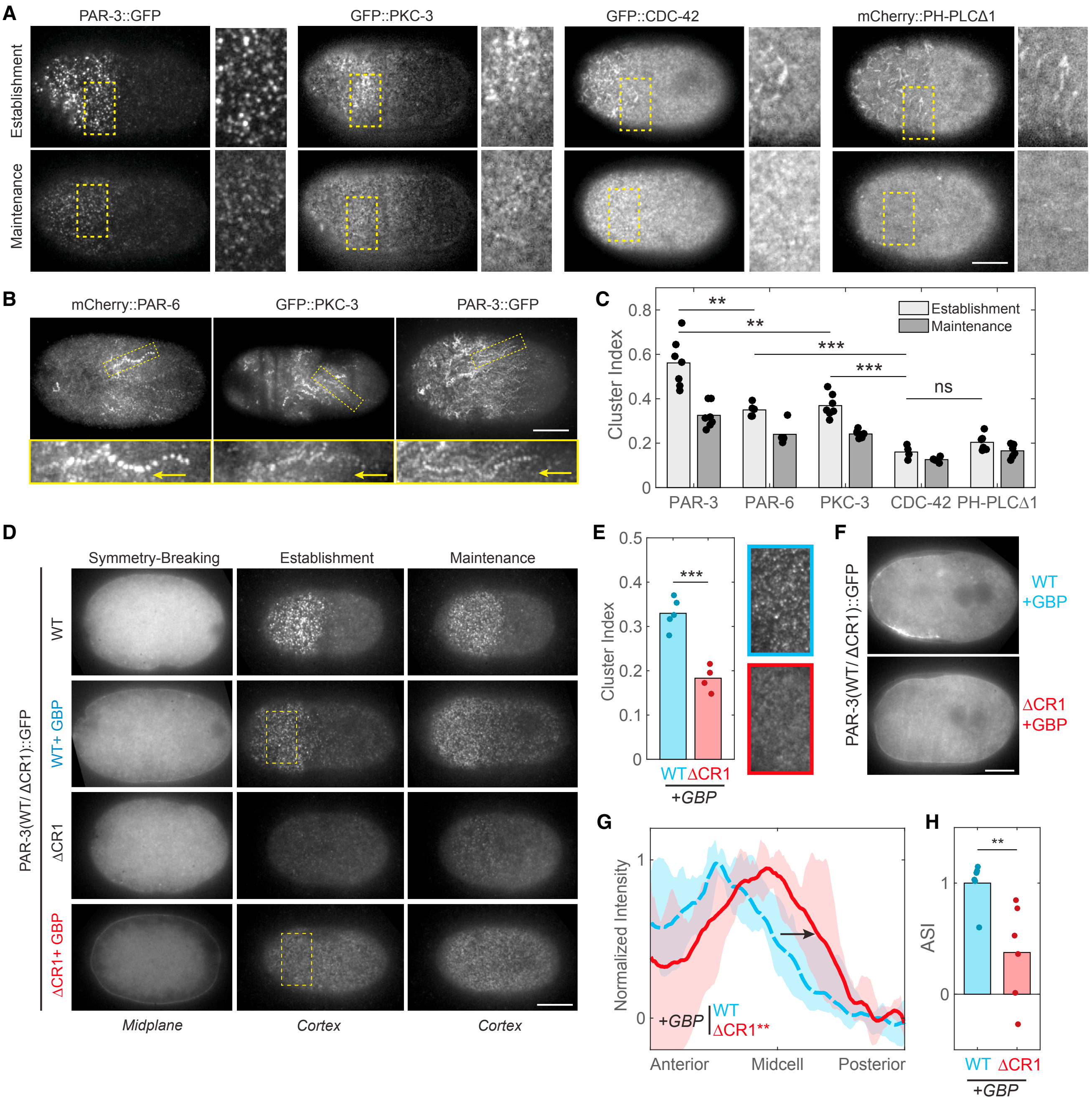

Figure 4. Segregation of Anterior PAR Proteins Involves Cortical Clusters

(A) Representative cortical images of PAR-3, PKC-3, CDC-42, and PH-PLCΔ1 in late-establishment and maintenance-phase zygotes along with zoom of inset region (yellow box).

(B) Time-averaged cortical images spanning 180 s reveal anterior-directed tracks of cortical clusters of PAR-3, PAR-6, and PKC-3. Insets highlight the motion (arrows) of a representative single cluster in the image above.

(C) Cluster index for the full dataset in (A) and PAR-6::GFP (images not shown). Significance between establishment and maintenance: $p < 0.01$ for PAR-3, PAR-6, and PKC-3.

(D) Representative midsection or cortical images of PAR-3::GFP (WT) or PAR-3ΔCR1::GFP (ΔCR1) with and without co-expression of the membrane tether PH::GBP (±GBP) shown at symmetry-breaking, establishment, or maintenance phase. Note enhancement of membrane signal visible in GBP-expressing zygotes viewed in midsection and lack of bright clusters of PAR-3ΔCR1::GFP viewed at the cortex.

(E) Cluster index for WT and ΔCR1 in the presence of the PH-GBP membrane tether (+GBP) along with magnification of insets (yellow dashed-line rectangles in D) indicated to highlight the difference in clustering.

(F) Representative midplane images of WT and ΔCR1 subject to GBP-membrane targeting showing defective segregation of ΔCR1. Images shown are from late-establishment phase.

(legend continued on next page)

PAR-6/aPKC are typically associated with an active, guanosine triphosphate (GTP)-bound form of CDC-42 (Atwood et al., 2007; Gotta et al., 2001; Joberty et al., 2000; Lin et al., 2000; Qiu et al., 2000), we found that decreasing CDC-42/GTP by depletion of the CDC-42 GEF, CGEF-1, reduces membrane association of PAR-6/PKC-3 in *pkc-3*(*ts*) embryos compared with wild-type, while leaving PAR-3 levels unchanged (Figures 3G and S5A–S5E). Finally, PKC-3 remains dependent on PAR-6 in PKC-3-inhibited embryos, consistent with PAR-6 being required to mediate the interactions of the PAR-6/PKC-3 heterodimer with PAR-3 and/or CDC-42 (Figures 3A and 3D–3F; Movie S4) (Joberty et al., 2000; Qiu et al., 2000), as well as our general findings that PKC-3 and PAR-6 respond similarly in all assays described.

Thus, inhibition of PKC-3 activity allows PKC-3 to bypass its normal requirement on PAR-3 to load onto the membrane and form stable membrane-associated CDC-42/GTP-dependent complexes. We postulate that it is this dependency on PAR-3, enforced by PKC-3 kinase activity, that ensures the coupled distributions of PKC-3 and PAR-3 in the embryo.

Segregation of Anterior PAR Proteins Is Associated with PAR-3-Dependent Clustering at the Membrane

So far, we have shown that inhibition of PKC-3 kinase activity promotes CDC-42-dependent assemblies, and in so doing prevents PKC-3 and PAR-6 from segregating efficiently with PAR-3 into the anterior during symmetry breaking. Previous work has shown that the efficient segregation of anterior PAR proteins is due to advective transport by anteriorly-directed actomyosin cortical flow (Cheeks et al., 2004; Goehring et al., 2011b; Munro et al., 2004). Because PAR-3 continues to be segregated efficiently in PKC-3-inhibited zygotes, we reasoned that PKC-3 inhibition may selectively alter the molecular organization of PAR-6 and PKC-3 at the membrane relative to PAR-3, which would be consistent with the observed shift toward CDC-42-dependent PKC-3 assemblies.

To investigate these possibilities, we imaged PAR-3, PAR-6, and PKC-3 at the membrane using variable-angle epifluorescence microscopy (VAEM or pseudo-TIRF) (Konopka and Bednarek, 2008). All three proteins exhibit a distinct clustered appearance during the polarity establishment phase, consistent with reports of non-homogenous distributions of PAR proteins at the membrane (Figures 4A and 5A, Establishment) (Munro et al., 2004; Robin et al., 2014; Sailer et al., 2015). Similar to previous analysis of PAR-6 (Munro et al., 2004), we find that clusters of PAR-6, PAR-3, and PKC-3 move in a highly directional manner in the direction of cortical flow, coinciding with increasing overall asymmetry (Figure 4B and Movie S5). While aPAR clusters have been noted (Hung and Kemphues, 1999; Munro et al., 2004; Sailer et al., 2015), the relationship between clustered and non-clustered PAR proteins and their ability to segregate in response to flow has not been explored.

To test whether clustering is a key driver of aPAR segregation, we examined whether PAR-3 transport depends on its ability to cluster. PAR-3 contains a conserved CR1 oligomerization domain, which is required for membrane binding and is targeted by PAR-1 kinase to induce displacement form the membrane (Benton and St Johnston, 2003; Feng et al., 2007; Li et al., 2010; Mizuno et al., 2003). We reasoned that this domain would be required for clustering; however, because mutations in the CR1 domain disrupt membrane binding (Figure 4D, WT versus ΔCR1), assessing clustering and segregation of a PAR-3ΔCR1 mutant requires an alternative mode of membrane targeting. We restored membrane localization of GFP:PAR-3ΔCR1 using a membrane-tethered anti-GFP nanobody (PH-GBP) and compared this with the behavior of GFP:PAR-3 (wild-type) that was also tethered to the membrane via PH-GBP. Targeting both wild-type and ΔCR1 to the membrane with PH-GBP reduces potential confounding effects of PAR-1-induced membrane displacement in the posterior. Thus, differences in segregation in PAR-3ΔCR1 relative to wild-type should be due to changes in clustering rather than differential sensitivity to PAR-1.

Consistent with oligomerization being required for clustering, membrane-tethered PAR-3ΔCR1 exhibited more diffuse membrane localization compared with wild-type controls (Figures 4D and 4E, WT + GBP versus ΔCR1 + GBP). PAR-3ΔCR1 also showed reductions in both segregation into the anterior and overall asymmetry (Figures 4F–4H and Movie S5). Thus, the ability of the CR1 domain to drive formation of membrane-associated PAR-3 clusters ensures that PAR-3 is efficiently transported by cortical flows in addition to its known role in promoting membrane association.

Balance between PAR-3 and CDC-42 Assemblies Tunes Cortical Organization and Sensitivity to Cortical Flow

The correlation between lack of PAR-3 clustering and defects in advective transport prompted us to examine the organization of anterior PAR proteins at the membrane in more detail. Although PAR-6 and PKC-3 exhibit a distinct clustered appearance similar to PAR-3 during polarity establishment, clusters are less pronounced and accompanied by a background of a more diffuse population (Figures 4A, 4C, and 5A). By contrast, CDC-42 exhibits a more uniform signal overall that resembles typical membrane markers, such as the PIP_2 (phosphatidylinositol-4,5-bisphosphate) probe PH-PLCΔ1 (Figures 4A and 4C). Membrane markers do exhibit enriched signals in membrane folds and protrusions, which are also enriched in the anterior, but these signals are clearly distinguishable from clusters.

With the transition into maintenance phase, clusters of PAR-6 and PKC-3 become less prominent and a diffuse population dominates. This change coincides temporally with a decrease in the prominence of PAR-3 clusters and an overall reduction in PAR-3 membrane localization (Figures 4A and 4C) as well as an increase in anterior CDC-42 activity (Figures S5C–S5E) (Kumfer et al., 2010). We therefore speculated that the mix of diffuse and clustered PAR-6/PKC-3 observed during establishment phase may reflect the distinct CDC-42- and PAR-3-dependent assemblies that we describe above. Consequently,

(G) Membrane intensity profiles for the full dataset represented in (F), showing average (solid line) and full range of data (shaded). Arrow highlights posterior expansion of the PAR-3 domain boundary.

(H) ASI quantification of membrane intensity profiles in (G) showing significant reduction in asymmetry in the ΔCR1 mutant.

**p < 0.01, ***p < 0.001. ns, not significant. Scale bars, 10 μm. See also Movie S5.

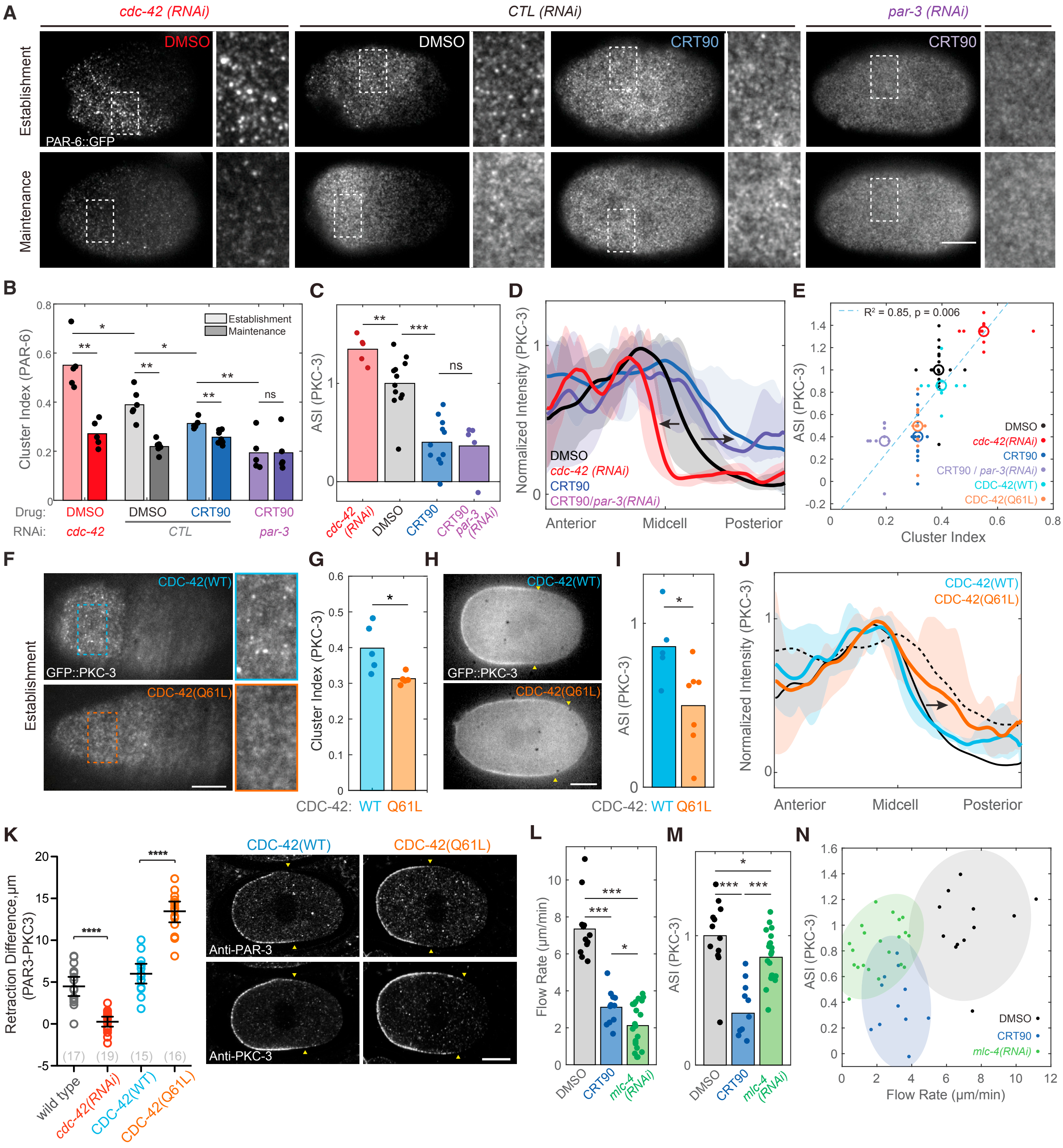

Figure 5. Regulation of PKC-3/PAR-6 Cluster Association by PAR-3/CDC-42 Balance Tunes Responsiveness to Cortical Flows

(A) Representative cortical images of PAR-6::GFP at late-establishment and maintenance-phase embryos for indicated conditions, shown along with a zoom of inset region (white boxes).

(B) Cluster index measurements of full dataset reveal a gradient of cluster association across conditions. Note that clustering decreases when embryos enter maintenance phase, except for CRT90/*par-3*(*RNAi*) embryos, which show minimal clustering even in establishment phase.

(C) ASI measurements of midsection images taken at late-establishment phase for a similar set of embryos as in (A) and (B), but expressing GFP::PKC-3, show a similar trend.

(D) Profiles of membrane signal for zygotes in (C) showing average (solid line) and full range of data (shaded) reveal shift of the PKC-3 domain boundary (arrows) toward the anterior in *cdc-42*(*RNAi*) embryos and toward the posterior in CRT90-treated zygotes, resulting in significantly ($p < 0.01$) smaller and larger domain sizes, respectively.

(legend continued on next page)

cell-cycle-dependent changes in the balance between assembly types could effectively tune the system to promote efficient transport of aPAR species during the polarity establishment phase.

To test this hypothesis, we altered the balance between CDC-42- and PAR-3-dependent assemblies and monitored the corresponding changes in (1) organization at the membrane, and (2) segregation efficiency. In general, we find a striking correlation between quantitative measures of cortical clustering and overall asymmetry.

Depletion of CDC-42 is known to increase co-localization of PAR-6 with PAR-3 during polarity establishment (Beers and Kemphues, 2006). We find that this also increases overall clustering of PAR-6 (Figures 5A and 5B). Examination of PKC-3 distributions clearly reveals enhanced segregation, with increased ASI (Figure 5C) and a visibly steeper domain boundary that is shifted toward the anterior (Figure 5D), resulting in a smaller anterior domain (*p < 0.01). Consistent with these data, we also observe a tighter coupling between the PAR-3 and PKC-3 domain boundaries in dual-labeled fixed zygotes (Figure 5K).

In contrast to CDC-42 depletion, inhibition of PKC-3 using CRT90, which favors CDC-42-dependent assemblies, shows reduced clustering of PAR-6, which could be reduced further by also depleting PAR-3 (Figures 5A and 5B). Under these conditions that favor CDC-42-dependent assemblies and reduced clustering, PKC-3 segregated less efficiently than DMSO controls, exhibited a reduced ASI, and failed to be fully excluded from the posterior (Figures 5C and 5D). To confirm that this reduction in clustering and segregation is due to favoring CDC-42-dependent assemblies, we examined the effect of expressing CDC-42 (Q61L), which stabilizes the active GTP-bound form of CDC-42 (Aceto et al., 2006; Ziman et al., 1991). Unlike PKC-3 inhibition, CDC-42 (Q61L) does not efficiently bypass the normal dependence of PKC-3 on PAR-3 (Figures S5F–S5J). This suggests that inhibition of PKC-3 favors CDC-42-associated assemblies via a mechanism distinct from stabilizing the GTP-bound form of CDC-42. However, similar to what we see when PKC-3 is inhibited, expression of CDC-42 (Q61L) reduced clustering and resulted in less efficient segregation of PKC-3 (Figures 5F–5K). Thus, regardless of the mechanism by which we alter the balance between PAR-3- and CDC-42-dependent assemblies, we achieve similar effects on clustering and segregation. This is particularly striking when we plot mean cluster index versus asymmetry across all conditions at establishment phase (Figure 5E).

Because PAR proteins are known to regulate actomyosin dynamics (Cheeks et al., 2004; Munro et al., 2004), and changes in flow velocities could, in principle, affect advective transport (Goehring et al., 2011b), we wanted to confirm that clustering rather than potential changes in flow velocity were the cause of reduced segregation efficiency. Measurements of flow rates from yolk granule motion in differential interference contrast (DIC) images allowed us to test the relationship between cortical flow rates and asymmetry in individual zygotes. Consistent with anterior PARs promoting their own segregation via stimulation of cortical flows, we find that PKC-3 inhibition results in a reduction of flow rates from approximately 6–10 μm/min in controls to approximately 2–5 μm/min in CRT90-treated embryos (Figure 5L). To test whether alterations in flow velocities could account for the observed segregation defects, we performed a partial depletion of MLC-4 to generate embryos with flow velocities of a similar range as observed in PKC-3-inhibited embryos (Figure 5L). Despite a similar range of flow velocities, MLC-4-depleted zygotes show a minimal reduction in asymmetry versus controls (Figures 5M and S6A). Plotting flow velocity versus ASI reveals a weak decline in ASI as flow rates are reduced (Figure 5N). By contrast, CRT90-treated embryos show a lower ASI across the full range of observed flow rates (~2–5 μm/min, Figures 5N and S6A). Finally, to test whether restoring flows could rescue efficient segregation of PKC-3, we used RNAi to target the RhoGAPs, RGA-3/4, which results in excess actomyosin contractility and increased cortical flow rates (Fievet et al., 2012; Schonegg et al., 2007). Despite fully rescuing the moderate reduction in asymmetry of PAR-3 observed in *pkc-3(ts)* embryos to levels indistinguishable from wild-type, RGA-3/4 depletion failed to restore asymmetry of PKC-3 (Figures S6B and S6C).

Together these data suggest that it is the failure of PKC-3 to associate with clusters rather than changes in flow rates that are the dominant factor in the decoupling between the localization of PAR-3 and PAR-6/PKC-3 observed in PKC-3-inhibited embryos. In fact, the resilience of PKC-3 asymmetry in embryos partially depleted of MLC-4 suggests that there is a relatively low threshold velocity required for efficient segregation of aPAR proteins by cortical flow, provided aPARs are able to associate normally into clusters.

We therefore conclude that although both PAR-3 and CDC-42 are critical for normal PAR-6/PKC-3 localization at the membrane in wild-type embryos, they drive formation of distinct aPAR assemblies, with distinct physical properties and

(E) Combining clustering data from pseudo-TIRF imaging in (A) and (B) and (F) and (G) with ASI measurements of a complete GFP::PKC-3 dataset for late-establishment phase across all conditions allows us to plot cluster index versus ASI for the mean of each condition, revealing a strong correlation (linear regression: $R^2 = 0.85$, $p < 0.01$).

(F–I) Representative cortical images (F, full zygote and inset zoom), cluster index (G), representative midsection images (H), and ASI (I) for late-establishment phase zygotes expressing GFP::PKC-3 in combination with either CDC-42(WT) or CDC-42 (Q61L). Yellow arrowheads in (H) highlight PKC-3 domain boundaries.

(J) Profiles of membrane signal for zygotes in (I) showing average (solid line) and full range of data (shaded) highlight the posterior shift (black arrow) of the PKC-3 boundary in Q61L-expressing zygotes ($p < 0.05$). Profiles for wild-type (solid black line) and CRT90-treated (dashed line) from (D) shown for comparison.

(K) Quantification of the difference in boundary position between PAR-3 and PKC-3 in dual-labeled fixed zygotes for indicated conditions. Mean ± 95% CI (N) is shown. Positive values indicate reduced PKC-3 segregation relative to PAR-3. Representative images of PAR-3 and PKC-3 in zygotes overexpressing CDC-42(WT) of CDC-42 (Q61L). Yellow arrowheads indicate the posterior boundary of the PAR-3 or PKC-3 domains.

(L and M) Comparison of cortical flow velocities (L) and PKC-3 asymmetry (M, ASI) for DMSO, CRT90, or *mlc-4(RNAi)* embryos taken at late-establishment phase.

(N) A plot of PKC-3 ASI versus cortical flow rates for individual zygotes treated with DMSO, CRT90, or *mlc-4(RNAi)*. Data points for individual embryos are shown with a 90% confidence boundary (shaded region).

*p < 0.05, **p < 0.01, ***p < 0.001, ****p < 0.0001. ns, not significant. Scale bars, 10 μm. See also Figure S6.

responsiveness to cortical flow: PAR-3-dependent assemblies exhibit pronounced clustering, at least during the establishment phase, and are efficiently segregated by cortical flow. By contrast, CDC-42-dependent assemblies are more diffuse, likely reflecting enhanced diffusional mobility, and are inefficiently segregated by flow. Importantly, the balance between these two species appears to be subject to cell-cycle-dependent regulation to ensure maximal clustering and transport during the period of peak actomyosin cortical flows.

A PKC-3 Membrane-Targeting Assay Reveals Opposing Roles for PAR-3 and CDC-42 in Regulating PKC-3 Activity

We next sought to explore whether there were other functional differences in these two types of assemblies. Specifically, we wondered whether PAR-3 and CDC-42 may have distinct regulatory effects on PKC-3 activity *in vivo*, which is difficult to analyze given their roles in PKC-3 membrane loading. While CDC-42 is generally thought to play an activating role (Atwood et al., 2007; Gotta et al., 2001; Joberty et al., 2000; Lin et al., 2000; Qiu et al., 2000), the roles for PAR-3 and PAR-6 are less clear and may vary in different contexts (Achilleos et al., 2010; Atwood et al., 2007; David et al., 2013; Graybill et al., 2012; Lin et al., 2000; McCaffrey and Macara, 2009; Wirtz-Peitz et al., 2008).

To directly assess whether PKC-3 activity differs in PAR-3-associated and CDC-42-associated assemblies *in vivo*, we targeted PKC-3 to the membrane by fusing it to the C1B domain of human PKCα, which can be induced to bind the membrane by the addition of phorbol ester (Figure 6A) (Lekomtsev et al., 2012). This bypasses the membrane-binding requirement of PKC-3 on PAR-3, PAR-6, and CDC-42, allowing us to test their contribution to PKC-3 activity by monitoring membrane removal of the PKC-3 target, PAR-2.

In the absence of phorbol ester, C1B-PKC-3 mirrors endogenous PKC-3 localization and is anteriorly enriched, with PAR-2 restricted to the posterior as in wild-type (Figure 6B, No PMA). Upon addition of 100 μM phorbol 12-myristate 13-acetate (PMA), C1B-PKC-3 is recruited uniformly to the membrane and the PAR-2 domain shrinks, consistent with an increase in posterior PKC-3 activity (Figure 6B, +PMA; Figure 6C; Movie S6). The reduction in PAR-2 domain size is not seen in the absence of PMA, when targeting the C1B domain alone to the membrane, or if we inhibit PKC-3 with CRT90 (Figures 6B and 6D). The failure to completely remove PAR-2 in polarized zygotes is not simply due to PAR-2 being concentrated in a domain, because ectopic PAR-2 domains that form in meiotic arrest mutants, e.g., *mei-1* and *emb-27* (Wallenfang and Seydoux, 2000), are rapidly cleared (Figure S7).

In *par-3*, *par-6*, or *cdc-42*(*RNAi*) zygotes, both endogenous PKC-3 and the C1B-PKC-3 fusion are cytoplasmic in the absence of PMA, allowing PAR-2 to localize uniformly to the membrane (Figures 6E and 6F). In *par-6* and *cdc-42*(*RNAi*) zygotes, membrane targeting of C1B-PKC-3 (+PMA) has no effect on PAR-2 distribution: it remains uniformly enriched at the membrane with no difference compared with controls in which C1B alone is targeted to the membrane (Figures 6E and 6F; Movie S7). Thus, both PAR-6 and CDC-42 are required for PKC-3 activity *in vivo*.

By contrast, membrane targeting of C1B-PKC-3 in *par-3*(*RNAi*) zygotes induces rapid loss of PAR-2 from the membrane, with near complete removal within minutes (Figures 6E and 6F; Movie S7). The displacement of PAR-2 is stronger than in wild-type zygotes, suggesting that PAR-3 normally acts to inhibit or suppress PKC-3 activity (Figure 6B, +PMA). Thus PAR-3 has two roles *in vivo*: it promotes PKC-3 membrane targeting while at the same time limiting its activation, reconciling *in vivo* reports whereby PAR-3 positively regulates PAR polarity (Achilleos et al., 2010; McCaffrey and Macara, 2009) with data indicating that PAR-3 can inhibit PKC-3 activity *in vitro* (Graybill et al., 2012; Lin et al., 2000; Soriano et al., 2016).

DISCUSSION

Taken together, our data suggest that efficient polarization requires PKC-3 to cycle between functionally specialized modules of the anterior PAR network: a PAR-3-dependent module that segregates in response to symmetry-breaking signals, but which is inactive, and a CDC-42-dependent module that uses spatial information provided by PAR-3 to create an anterior domain of PKC-3 activity on the membrane (Figure 7).

In previous work, we showed that the diffusion and membrane dissociation rates of aPARs were in principle sufficient to explain segregation in response to flows (Goehring et al., 2011b). Here we show that segregation of aPARs is directly linked to PAR-3-dependent clustering. Clustering reduces the effective diffusion of membrane-associated aPARs, which should favor advective transport. Alternatively, the sheer size of clusters may allow them to sense flow that would not affect individual proteins, possibly by allowing them to extend into the cortical actomyosin layer. Regardless of the physical mechanism, as we show here, clustering drives robust segregation of PAR-3 by cortical flow. This fact, coupled with PAR-3 exclusion from the posterior by PAR-1-dependent phosphorylation (Motegi et al., 2011), supports a model in which PAR-3 is responsible for sensing asymmetry-generating cues. Importantly, once it is asymmetric, PAR-3 provides a landmark for polarized loading of PAR-6/PKC-3, explaining recent observations that PAR-6 loads preferentially in the anterior of polarized embryos (Sailer et al., 2015).

Because our *in vivo* PKC-3 activity assay indicates that PKC-3 activity is suppressed within PAR-3-dependent assemblies, PAR-6/PKC-3 molecules must be converted into an activated CDC-42-dependent species, a state that we show is non-clustered and diffusive. Whereas this diffusive behavior of CDC-42-assemblies is a disadvantage for transport by cortical flow, it is an advantage for creating a uniform, wider-range field of PKC-3 activity that can efficiently exclude pPARs. If the same complexes had to respond to flow and exclude pPARs, there would be a trade-off between efficiency of transport by flow and uniformity of pPAR inhibition at the anterior.

For this field of activated CDC-42-dependent PKC-3 assemblies to remain coupled to the spatial information provided by PAR-3, two conditions must be satisfied. First, PKC-3 membrane localization must be dependent on PAR-3, and second, diffusion of CDC-42-associated PKC-3 away from loading sites must be limited, with PKC-3 ultimately being released back into the cytoplasm, where it again becomes dependent on PAR-3. This turnover restricts the effective distance these

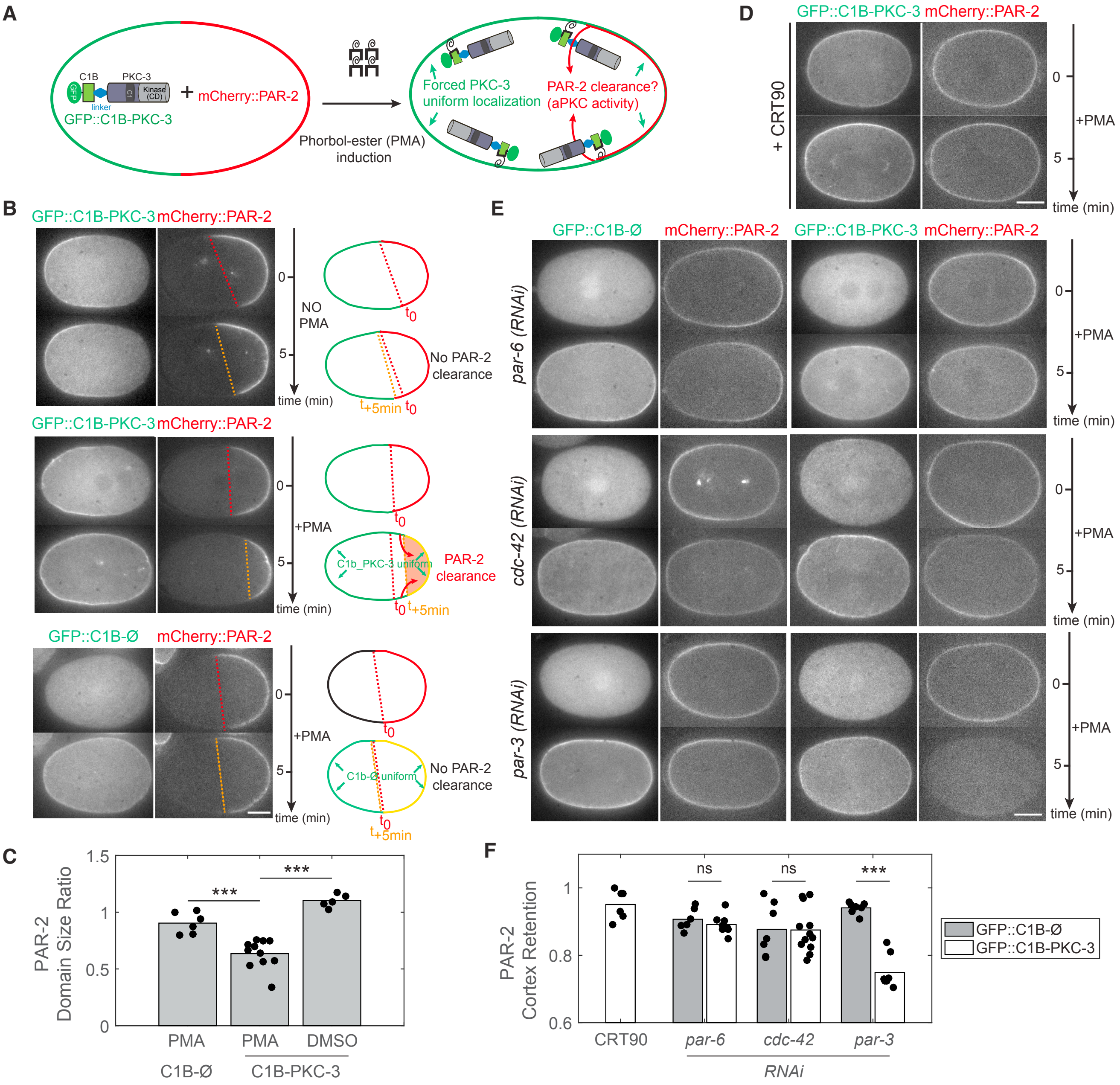

Figure 6. PAR-3 and CDC-42 Have Opposing Regulatory Roles in an *In Vivo* PKC-3 Activity Assay

(A) C1B targeting strategy for inducing PKC-3 membrane loading by PMA. PKC-3 kinase activity is monitored by following loss of PAR-2 from the membrane.

(B) Zygotes expressing GFP::C1B alone (GFP::C1B-Ø) or GFP::C1B-PKC-3 along with mCherry::PAR-2 were subject to the indicated treatment. Note that uniform membrane targeting of C1B-PKC-3 leads to reduction of PAR-2 domain size, whereas omitting PMA or expressing C1B alone has no effect. Right: cartoon representation of results.

(C) Quantification of PAR-2 domain size ratio for embryos shown in (B).

(D) PAR-2 retention in GFP::C1B-PKC-3 expressing zygotes treated with PMA and CRT90 confirms that induced PAR-2 loss is dependent on PKC-3 kinase activity.

(E) Zygotes expressing mCherry::PAR-2 with GFP::C1B-Ø or GFP::C1B-PKC-3 subject to *par-6*, *cdc-42*, or *par-3*(*RNAi*) before and 5 min after PMA addition.

(F) Quantification of PAR-2 cortex retention comparing GFP::C1B-PKC-3 and GFP::C1B-Ø zygotes after treatment with PMA as in (E).

Representative midsection confocal images are shown in (B), (D), and (E) before and 5 min after PMA/DMSO addition. ***$p < 0.001$. ns, not significant. Scale bars, 10 μm. See also Figure S7; Movies S6 and S7.

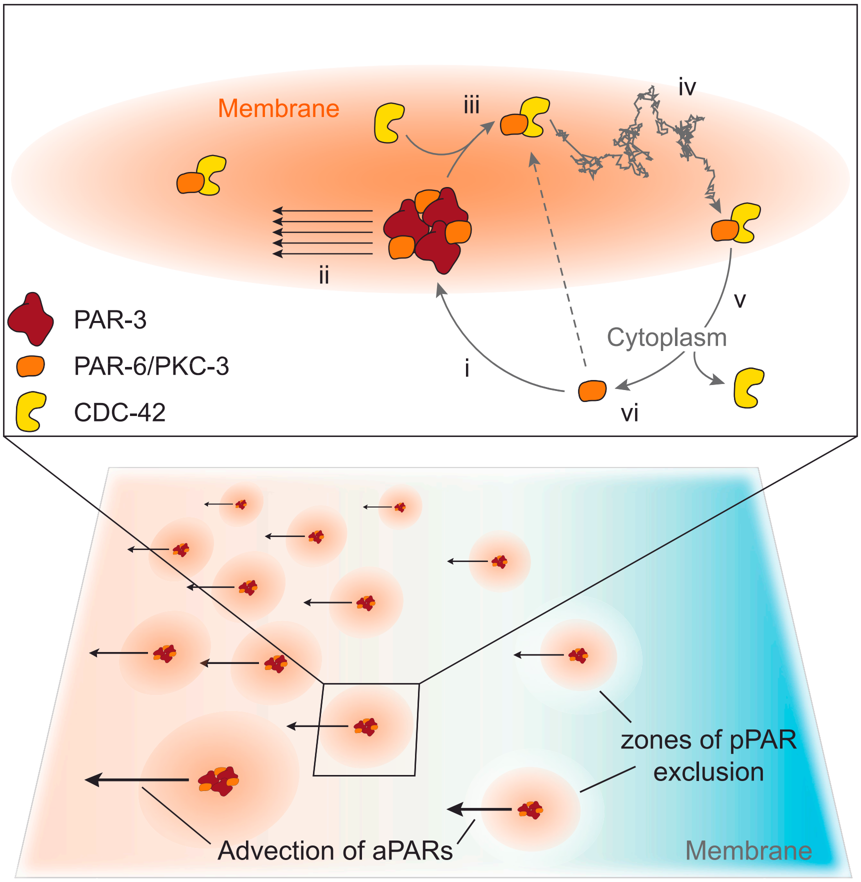

Figure 7. Polarization through Coupling of PAR-3- and CDC-42-Dependent aPAR Assemblies

(i) PKC-3 kinase activity ensures that PKC-3 loads via a PAR-3 intermediate, in which PKC-3 activity is suppressed. This dependence on PAR-3 can be bypassed upon inhibition of PKC-3 (dashed arrow). (ii) Clustering of membrane-associated PAR-3 allows it to be segregated by cortical flow into the anterior, carrying along associated PKC-3 molecules and generating asymmetric sites for further PKC-3 loading. (iii) PKC-3 activation requires conversion into a CDC-42-associated assembly, which relieves inhibition of PKC-3 by PAR-3. (iv) The CDC-42-dependent module is freely diffusible on the membrane and locally excludes pPARs. (v) Dissociation of CDC-42-dependent assemblies limits the spread of active PKC-3 at the membrane from the PAR-3 recruiting site. (vi) PKC-3 returns to the cytoplasm where it must load again via PAR-3.

complexes can diffuse from their initial sites of formation, defining an effective "sphere of influence" around PAR-3 loading sites. Together, these requirements result in a cycle of localized membrane loading, activation, and release (Figure 7).

Our data suggest that the first of these requirements, PAR-3-dependent loading, is dependent on the kinase activity of PKC-3 itself, although the precise mechanism is unclear. Given the limited ability of CDC-42 (Q61L) to rescue PKC-3 membrane localization in PAR-3-depleted embryos, PKC-3 is likely to act at the step of CDC-42 complex generation, either inhibiting its own association with CDC-42, and/or destabilizing nascent CDC-42/PAR-6/PKC-3 assemblies. We speculate that it could be the very act of inhibiting PKC-3 through which PAR-3 promotes generation of stable CDC-42-dependent assemblies, but further work will be required to reveal the details of this molecular handover. Because the inhibitory role of PAR-3 appears to be broadly conserved (David et al., 2013; Graybill et al., 2012; Lin et al., 2000; McCaffrey et al., 2012; Soriano et al., 2016), this apparent paradoxical role of PAR-3 in promoting formation of membrane-associated aPKC complexes, yet also suppressing PKC-3 activity, may be a general feature of aPKC regulation.

How diffusion of CDC-42-associated PKC-3 is limited also remains unclear. Measurements elsewhere suggest that the distance these activated assemblies travel is on the order of 5–10 μm (Goehring et al., 2011a; Robin et al., 2014), consistent with the PKC-3 gradient extending further into the posterior than PAR-3 during the establishment phase even in wild-type embryos (see Figure 2I). Because CDC-42 assemblies appear to be resistant to removal by posterior PAR proteins (Figure 2M), including the CDC-42 GAP CHIN-1, it seems likely that it is not preferential removal of these complexes in the posterior by pPARs, but rather the intrinsic lifetime of CDC-42-dependent PKC-3 assemblies that limits their diffusion into the posterior. This is compatible with a model in which aPKC undergoes asymmetric membrane loading but symmetric dissociation (Robin et al., 2014).

By loading PKC-3 via what we presume is an inhibited PAR-3-associated state, which must then be activated, PKC-3 localization and activation are segregated into distinct modules of the PAR network, which can be regulated independently. This division of labor may be critical for PAR proteins, which operate across diverse contexts, where the polarity cues, substrates, scales, and even the concentrations of PAR molecules themselves may vary substantially. Even within the *C. elegans* zygote, the mechanisms of PAR segregation vary. During polarity establishment, when cortical flow is the major cue for anterior PAR segregation, PAR-3 clustering is prominent (Cheeks et al., 2004; Goehring et al., 2011b; Munro et al., 2004). As the system enters the maintenance phase, flow ceases and continued aPAR segregation becomes dependent on the activity of PAR-1 and CHIN-1 (Beatty et al., 2013; Guo and Kemphues, 1995; Kumfer et al., 2010; Sailer et al., 2015). Notably, clustering appears to be reduced during this phase, which Dickinson et al. (2017) show is dependent on PLK-1. This change in PAR molecular organization potentially reflects the shift in the spatial signals to which the PAR network must respond. At the same time, despite these changes, PKC-3 activity must remain efficient at displacing pPARs from the anterior domain, highlighting the adaptability of the PAR network.

In summary, here we have identified a critical role for the separation of signal-receiving and signal-transducing functions between modules of the aPAR network that are distinct, but

coupled via dynamic exchange of the shared signaling component, PKC-3. We suggest that functional specialization of coupled modules resolves potential molecular constraints between components that must be sensitive to polarity cues and those that must propagate the signals. It further allows the system to independently modulate responsiveness to cues as well as the extent and strength of the output signal. The adaptability of such a paradigm suggests it is likely to be a common strategy in patterning systems.

STAR★METHODS

Detailed methods are provided in the online version of this paper and include the following:

- KEY RESOURCES TABLE
- CONTACT FOR REAGENT AND RESOURCE SHARING
- EXPERIMENTAL MODEL AND SUBJECT DETAILS
  - *C. elegans* Strains and Maintenance
  - *C. elegans* Transgenic Animals
  - Cell Lines
  - Bacterial Strains
- METHOD DETAILS
  - *C. elegans* - RNAi Culture Conditions
  - *C. elegans* Embryos – Western Blots
  - *C. elegans* Zygotes – Drug Treatment
  - *C. elegans* Zygotes - Immunofluorescence
  - *C. elegans zygotes* - Live Imaging
  - *In vitro* PKC Enzyme Assays
  - Cellular Biochemical Assay
  - Kinase Selectivity
- QUANTIFICATION AND STATISTICAL ANALYSIS
  - Image Analysis - General
  - Image Analysis - Asymmetry Index (ASI)
  - Image Analysis – Cluster Index
  - Image Analysis – CHIN-1 Foci
  - Image Analysis – MEX-5 Mobility
  - Image Analysis - Colocalization
  - Image Analysis – Flow Speeds
  - Image Analysis – Anterior Cortical Intensity (Immunofluorescence)
  - Image Analysis – Anterior PAR Retraction (Immunofluorescence)
  - Image Analysis – Intensity Profile Extraction
  - Image Analysis – PAR-3 versus PKC-3 Cortical Profile Comparison (Immunofluorescence)
  - Image Analysis – Domain Size/Segregation Efficiency
  - Image Analysis – Total Membrane Signal Change
  - Statistics
  - Data and Software Availability

SUPPLEMENTAL INFORMATION

Supplemental Information includes seven figures and seven movies and can be found with this article online at http://dx.doi.org/10.1016/j.devcel.2017.07.007.

AUTHOR CONTRIBUTIONS

Conceptualization, J. Rodriguez, F.P., and N.W.G.; Methodology, J. Rodriguez, F.P., J.M., L.H., J. Reich, N.H. and N.W.G.; Investigation, J. Rodriguez, F.P., J.M., L.H., N.H, A.G.G., A.R.F., and N.W.G.; Resources, J. Roffey; Writing – Original Draft, J. Rodriguez, F.P., and N.W.G.; Writing – Review & Editing, J. Rodriguez, F.P., D.StJ., J.A., and N.W.G.; Funding Acquisition, J. Rodriguez, D.StJ., J.A., and N.W.G.; Supervision, J. Rodriguez, D.StJ., J.A., and N.W.G. To satisfy Cell Press guidelines, one corresponding author was chosen at random to serve as Lead Contact, although both will be maintaining the reagents.

ACKNOWLEDGMENTS

The authors wish to thank Nic Tapon, Elizabeth Veal, Jonathan Higgins and members of the N.W.G. and J. Rodriguez laboratories for critical comments, Neil McDonald for helpful discussions, Ken Kemphues for his generosity in providing unpublished strains and PKC-3 antibody, Monica Gotta for providing PAR-6 antibody, and Dan Dickinson for sharing results prior to publication. This work was supported by a Faculty Fellowship from Newcastle University and a Royal Society Research Grant (RG2015R2 to J. Rodriguez), a BBSRC PhD fellowship (J.M.), a PhD fellowship from Newcastle University (A.G.G.), Wellcome Trust Senior and Principal Research Fellowships (054523, to J.A.; 080007, to D.StJ.), a University of Cambridge Studentship via the Wellcome Trust PhD Program in Developmental Biology (A.R.F.), and the Francis Crick Institute (N.W.G.), which receives its core funding from Cancer Research UK (FC001086), the UK Medical Research Council (FC001086), and the Wellcome Trust (FC001086). N.W.G. and J. Rodriguez are members of the GENiE network supported by COST Action BM1408 and EMBO. Some strains were provided by the CGC, which is funded by NIH Office of Research Infrastructure Programs (P40 OD010440). The P4A1 monoclonal antibody developed by J. Priess was obtained from the Developmental Studies Hybridoma Bank developed under the auspices of the NICHD and maintained by The University of Iowa, Department of Biology, Iowa City, IA 52242. J. Roffey is an employee of Cancer Research Technology Ltd. (CRT), a company registered in England and Wales under number 1626049 with registered office at Angel Building, 407 St John Street London, EC1V 4AD, England. CRT is wholly owned by Cancer Research UK (a charity and company registered in England and Wales with registered charity number 1089464 and registered company number 4325234).

Received: November 17, 2016
Revised: June 19, 2017
Accepted: July 10, 2017
Published: August 3, 2017

REFERENCES

Aceto, D., Beers, M., and Kemphues, K.J. (2006). Interaction of PAR-6 with CDC-42 is required for maintenance but not establishment of PAR asymmetry in *C. elegans*. Dev. Biol. *299*, 386–397.

Achilleos, A., Wehman, A.M., and Nance, J. (2010). PAR-3 mediates the initial clustering and apical localization of junction and polarity proteins during *C. elegans* intestinal epithelial cell polarization. Development *137*, 1833–1842.

Andrews, R., and Ahringer, J. (2007). Asymmetry of early endosome distribution in *C. elegans* embryos. PLoS One *2*, e493.

Atwood, S.X., Chabu, C., Penkert, R.R., Doe, C.Q., and Prehoda, K.E. (2007). Cdc42 acts downstream of Bazooka to regulate neuroblast polarity through Par-6 aPKC. J. Cell Sci. *120*, 3200–3206.

Audhya, A., Hyndman, F., McLeod, I.X., Maddox, A.S., Yates, J.R., Desai, A., and Oegema, K. (2005). A complex containing the Sm protein CAR-1 and the RNA helicase CGH-1 is required for embryonic cytokinesis in *Caenorhabditis elegans*. J. Cell Biol. *171*, 267–279.

Beatty, A., Morton, D., and Kemphues, K. (2010). The *C. elegans* homolog of *Drosophila* Lethal giant larvae functions redundantly with PAR-2 to maintain polarity in the early embryo. Development *137*, 3995–4004.

Beatty, A., Morton, D.G., and Kemphues, K. (2013). PAR-2, LGL-1 and the CDC-42 GAP CHIN-1 act in distinct pathways to maintain polarity in the *C. elegans* embryo. Development *140*, 2005–2014.

Beers, M., and Kemphues, K. (2006). Depletion of the co-chaperone CDC-37 reveals two modes of PAR-6 cortical association in *C. elegans* embryos. Development *133*, 3745–3754.

Benton, R., and St Johnston, D. (2003). A conserved oligomerization domain in *Drosophila* bazooka/PAR-3 is important for apical localization and epithelial polarity. Curr. Biol. *13*, 1330–1334.

Betschinger, J., Mechtler, K., and Knoblich, J.A. (2003). The Par complex directs asymmetric cell division by phosphorylating the cytoskeletal protein Lgl. Nature *422*, 326–330.

Boyd, L., Guo, S., Levitan, D., Stinchcomb, D.T., and Kemphues, K.J. (1996). PAR-2 is asymmetrically distributed and promotes association of P granules and PAR-1 with the cortex in *C. elegans* embryos. Development *122*, 3075–3084.

Brenner, S. (1974). The genetics of *Caenorhabditis elegans*. Genetics *77*, 71–94.

Carvalho, A., Olson, S.K., Gutierrez, E., Zhang, K., Noble, L.B., Zanin, E., Desai, A., Groisman, A., and Oegema, K. (2011). Acute drug treatment in the early *C. elegans* embryo. PLoS One *6*, e24656.

Caussinus, E., Kanca, O., and Affolter, M. (2011). Fluorescent fusion protein knockout mediated by anti-GFP nanobody. Nat. Struct. Mol. Biol. *19*, 117–121.

Cheeks, R.J., Canman, J.C., Gabriel, W.N., Meyer, N., Strome, S., and Goldstein, B. (2004). *C. elegans* PAR proteins function by mobilizing and stabilizing asymmetrically localized protein complexes. Curr. Biol. *14*, 851–862.

Cuenca, A.A., Schetter, A., Aceto, D., Kemphues, K., and Seydoux, G. (2003). Polarization of the *C. elegans* zygote proceeds via distinct establishment and maintenance phases. Development *130*, 1255–1265.

David, D.J.V., Wang, Q., Feng, J.J., and Harris, T.J.C. (2013). Bazooka inhibits aPKC to limit antagonism of actomyosin networks during amnioserosa apical constriction. Development *140*, 4719–4729.

Dickinson, D.J., Ward, J.D., Reiner, D.J., and Goldstein, B. (2013). Engineering the *Caenorhabditis elegans* genome using Cas9-triggered homologous recombination. Nat. Methods *10*, 1028–1034.

Dickinson, D.J., Schwager, F., Pintard, L., Gotta, M., and Goldstein, B. (2017). Dev. Cell *42*, this issue, in press.

Dong, Y., Bogdanova, A., Habermann, B., Zachariae, W., and Ahringer, J. (2007). Identification of the *C. elegans* anaphase promoting complex subunit Cdc26 by phenotypic profiling and functional rescue in yeast. BMC Dev. Biol. *7*, 19.

Dorsey, B.D., Learn, K.S., Morris, E.L., Ott, G.R., Roffey, J.R., Soudy, C.N., and Wagner, J.C. (2013). Thienopyrimidine inhibitors of atypical protein kinase C. Patent WO 2013078126 A1, filed 19 November, 2012.

Edgar, L.G., and Goldstein, B. (2012). Culture and manipulation of embryonic cells. Methods Cell Biol. *107*, 151–175.

Etemad-Moghadam, B., Guo, S., and Kemphues, K.J. (1995). Asymmetrically distributed PAR-3 protein contributes to cell polarity and spindle alignment in early *C. elegans* embryos. Cell *83*, 743–752.

Feng, W., Wu, H., Chan, L.-N., and Zhang, M. (2007). The Par-3 NTD adopts a PB1-like structure required for Par-3 oligomerization and membrane localization. EMBO J. *26*, 2786–2796.

Fievet, B.T., Rodriguez, J., Naganathan, S., Lee, C., Zeiser, E., Ishidate, T., Shirayama, M., Grill, S.W., and Ahringer, J. (2012). Systematic genetic interaction screens uncover cell polarity regulators and functional redundancy. Nat. Cell Biol. *15*, 103–112.

Goehring, N.W. (2014). PAR polarity: from complexity to design principles. Exp. Cell Res. *328*, 258–266.

Goehring, N.W., Hoege, C., Grill, S.W., and Hyman, A.A. (2011a). PAR proteins diffuse freely across the anterior-posterior boundary in polarized *C. elegans* embryos. J. Cell Biol. *193*, 583–594.

Goehring, N.W., Trong, P.K., Bois, J.S., Chowdhury, D., Nicola, E.M., Hyman, A.A., and Grill, S.W. (2011b). Polarization of PAR proteins by advective triggering of a pattern-forming system. Science *334*, 1137–1141.

Goldstein, B., and Macara, I.G. (2007). The PAR proteins: fundamental players in animal cell polarization. Dev. Cell *13*, 609–622.

Gotta, M., Abraham, M.C., and Ahringer, J. (2001). CDC-42 controls early cell polarity and spindle orientation in *C. elegans*. Curr. Biol. *11*, 482–488.

Graybill, C., Wee, B., Atwood, S.X., and Prehoda, K.E. (2012). Partitioning-defective protein 6 (Par-6) activates atypical protein kinase C (aPKC) by pseudosubstrate displacement. J. Biol. Chem. *287*, 21003–21011.

Griffin, E.E., Odde, D.J., and Seydoux, G. (2011). Regulation of the MEX-5 gradient by a spatially segregated kinase/phosphatase cycle. Cell *146*, 955–968.

Guo, S., and Kemphues, K.J. (1995). par-1, a gene required for establishing polarity in *C. elegans* embryos, encodes a putative Ser/Thr kinase that is asymmetrically distributed. Cell *81*, 611–620.

Hao, Y., Boyd, L., and Seydoux, G. (2006). Stabilization of cell polarity by the *C. elegans* RING protein PAR-2. Dev. Cell *10*, 199–208.

Harris, T.J.C., and Peifer, M. (2005). The positioning and segregation of apical cues during epithelial polarity establishment in Drosophila. J. Cell Biol. *170*, 813–823.

Hoege, C., Constantinescu, A.-T., Schwager, A., Goehring, N.W., Kumar, P., and Hyman, A.A. (2010). LGL can partition the cortex of one-cell *Caenorhabditis elegans* embryos into two domains. Curr. Biol. *20*, 1296–1303.

Hoege, C., and Hyman, A.A. (2013). Principles of PAR polarity in *Caenorhabditis elegans* embryos. Nat. Rev. Mol. Cell Biol. *14*, 315–322.

Hung, T.J., and Kemphues, K.J. (1999). PAR-6 is a conserved PDZ domain-containing protein that colocalizes with PAR-3 in *Caenorhabditis elegans* embryos. Development *126*, 127–135.

Hurov, J.B., Watkins, J.L., and Piwnica-Worms, H. (2004). Atypical PKC phosphorylates PAR-1 kinases to regulate localization and activity. Curr. Biol. *14*, 736–741.

Izumi, Y., Hirose, T., Tamai, Y., Hirai, S., Nagashima, Y., Fujimoto, T., Tabuse, Y., Kemphues, K.J., and Ohno, S. (1998). An atypical PKC directly associates and colocalizes at the epithelial tight junction with ASIP, a mammalian homologue of *Caenorhabditis elegans* polarity protein PAR-3. J. Cell Biol. *143*, 95–106.

Joberty, G., Petersen, C., Gao, L., and Macara, I.G. (2000). The cell-polarity protein Par6 links Par3 and atypical protein kinase C to Cdc42. Nat. Cell Biol. *2*, 531–539.

Kachur, T.M., Audhya, A., and Pilgrim, D.B. (2008). UNC-45 is required for NMY-2 contractile function in early embryonic polarity establishment and germline cellularization in *C. elegans*. Dev. Biol. *314*, 287–299.

Kamath, R.S., Fraser, A.G., Dong, Y., Poulin, G., Durbin, R., Gotta, M., Kanapin, A., Le Bot, N., Moreno, S., Sohrmann, M., et al. (2003). Systematic functional analysis of the *Caenorhabditis elegans* genome using RNAi. Nature *421*, 231–237.

Kay, A.J., and Hunter, C.P. (2001). CDC-42 regulates PAR protein localization and function to control cellular and embryonic polarity in *C. elegans*. Curr. Biol. *11*, 474–481.

Kjær, S., Linch, M., Purkiss, A., Kostelecky, B., Knowles, P.P., Rosse, C., Riou, P., Soudy, C., Kaye, S., Patel, B., et al. (2013). Adenosine-binding motif mimicry and cellular effects of a thieno[2,3-d]pyrimidine-based chemical inhibitor of atypical protein kinase C isoenzymes. Biochem. J. *451*, 329–342.

Konopka, C.A., and Bednarek, S.Y. (2008). Variable-angle epifluorescence microscopy: a new way to look at protein dynamics in the plant cell cortex. Plant J. *53*, 186–196.

Kumfer, K.T., Cook, S.J., Squirrell, J.M., Eliceiri, K.W., Peel, N., O'Connell, K.F., and White, J.G. (2010). CGEF-1 and CHIN-1 regulate CDC-42 activity during asymmetric division in the *Caenorhabditis elegans* embryo. Mol. Biol. Cell *21*, 266–277.

Lekomtsev, S., Su, K.-C., Pye, V.E., Blight, K., Sundaramoorthy, S., Takaki, T., Collinson, L.M., Cherepanov, P., Divecha, N., and Petronczki, M. (2012). Centralspindlin links the mitotic spindle to the plasma membrane during cytokinesis. Nature *492*, 276–279.

Li, B., Kim, H., Beers, M., and Kemphues, K. (2010). Different domains of *C. elegans* PAR-3 are required at different times in development. Dev. Biol. *344*, 745–757.

Li, Q., Lau, A., Morris, T.J., Guo, L., Fordyce, C.B., and Stanley, E.F. (2004). A syntaxin 1, Gα(o), and N-type calcium channel complex at a presynaptic nerve terminal: analysis by quantitative immunocolocalization. J. Neurosci. *24*, 4070–4081.

Lin, D., Edwards, A.S., Fawcett, J.P., Mbamalu, G., Scott, J.D., and Pawson, T. (2000). A mammalian PAR-3-PAR-6 complex implicated in Cdc42/Rac1 and aPKC signalling and cell polarity. Nat. Cell Biol. *2*, 540–547.

Mayer, M., Depken, M., Bois, J.S., Jülicher, F., and Grill, S.W. (2010). Anisotropies in cortical tension reveal the physical basis of polarizing cortical flows. Nature *467*, 617–621.

McCaffrey, L.M., and Macara, I.G. (2012). Signaling pathways in cell polarity. Cold Spring Harb. Perspect. Biol. *4*.

McCaffrey, L.M., and Macara, I.G. (2009). The Par3/aPKC interaction is essential for end bud remodeling and progenitor differentiation during mammary gland morphogenesis. Genes Dev. *23*, 1450–1460.

McCaffrey, L.M., Montalbano, J., Mihai, C., and Macara, I.G. (2012). Loss of the Par3 polarity protein promotes breast tumorigenesis and metastasis. Cancer Cell *22*, 601–614.

Mizuno, K., Suzuki, A., Hirose, T., Kitamura, K., Kutsuzawa, K., Futaki, M., Amano, Y., and Ohno, S. (2003). Self-association of PAR-3-mediated by the conserved N-terminal domain contributes to the development of epithelial tight junctions. J. Biol. Chem. *278*, 31240–31250.

Morais-De-Sa, E., Mirouse, V., and St Johnston, D. (2010). aPKC phosphorylation of Bazooka defines the apical/lateral border in *Drosophila* epithelial cells. Cell *141*, 509–523.

Motegi, F., and Seydoux, G. (2013). The PAR network: redundancy and robustness in a symmetry-breaking system. Philos. Trans. R. Soc. Lond. B Biol. Sci. *368*, 20130010.

Motegi, F., and Sugimoto, A. (2006). Sequential functioning of the ECT-2 RhoGEF, RHO-1 and CDC-42 establishes cell polarity in *Caenorhabditis elegans* embryos. Nat. Cell Biol. *8*, 978–985.

Motegi, F., Zonies, S., Hao, Y., Cuenca, A.A., Griffin, E.E., and Seydoux, G. (2011). Microtubules induce self-organization of polarized PAR domains in *Caenorhabditis elegans* zygotes. Nat. Cell Biol. *13*, 1361–1367.

Munro, E., Nance, J., and Priess, J.R. (2004). Cortical flows powered by asymmetrical contraction transport PAR proteins to establish and maintain anterior-posterior polarity in the early *C. elegans* embryo. Dev. Cell *7*, 413–424.

Neukomm, L.J., Zeng, S., Frei, A.P., Huegli, P.A., and Hengartner, M.O. (2014). Small GTPase CDC-42 promotes apoptotic cell corpse clearance in response to PAT-2 and CED-1 in *C. elegans*. Cell Death Differ. *21*, 845–853.

Pelletier, V., Gal, N., Fournier, P., and Kilfoil, M.L. (2009). Microrheology of microtubule solutions and actin-microtubule composite networks. Phys. Rev. Lett. *102*, 188303.

Praitis, V., Casey, E., Collar, D., and Austin, J. (2001). Creation of low-copy integrated transgenic lines in *Caenorhabditis elegans*. Genetics *157*, 1217–1226.

Qiu, R.G., Abo, A., and Steven Martin, G. (2000). A human homolog of the *C. elegans* polarity determinant Par-6 links Rac and Cdc42 to PKCzeta signaling and cell transformation. Curr. Biol. *10*, 697–707.

Redemann, S., Pecreaux, J., Goehring, N.W., Khairy, K., Stelzer, E.H.K., Hyman, A.A., and Howard, J. (2010). Membrane invaginations reveal cortical sites that pull on mitotic spindles in one-cell *C. elegans* embryos. PLoS One *5*, e12301.

Redemann, S., Schloissnig, S., Ernst, S., Pozniakowsky, A., Ayloo, S., Hyman, A.A., and Bringmann, H. (2011). Codon adaptation-based control of protein expression in *C. elegans*. Nat. Methods *8*, 250–252.

Robin, F.B., McFadden, W.M., Yao, B., and Munro, E.M. (2014). Single-molecule analysis of cell surface dynamics in *Caenorhabditis elegans* embryos. Nat. Methods *11*, 677–682.

Sailer, A., Anneken, A., Li, Y., Lee, S., and Munro, E. (2015). Dynamic opposition of clustered proteins stabilizes cortical polarity in the *C. elegans* zygote. Dev. Cell *35*, 131–142.

Schindelin, J., Arganda-Carreras, I., Frise, E., Kaynig, V., Longair, M., Pietzsch, T., Preibisch, S., Rueden, C., Saalfeld, S., Schmid, B., et al. (2012). Fiji: an open-source platform for biological-image analysis. Nat. Methods *9*, 676–682.

Schonegg, S., and Hyman, A.A. (2006). CDC-42 and RHO-1 coordinate acto-myosin contractility and PAR protein localization during polarity establishment in *C. elegans* embryos. Development *133*, 3507–3516.

Schonegg, S., Constantinescu, A.T., Hoege, C., and Hyman, A.A. (2007). The Rho GTPase-activating proteins RGA-3 and RGA-4 are required to set the initial size of PAR domains in *Caenorhabditis elegans* one-cell embryos. Proc. Natl. Acad. Sci. USA *104*, 14976–14981.

Seitz, A., and Surrey, T. (2006). Processive movement of single kinesins on crowded microtubules visualized using quantum dots. EMBO J. *25*, 267–277.

Soriano, E.V., Ivanova, M.E., Fletcher, G., Riou, P., Knowles, P.P., Barnouin, K., Purkiss, A., Kostelecky, B., Saiu, P., Linch, M., et al. (2016). aPKC inhibition by Par3 CR3 flanking regions controls substrate access and underpins apical-junctional polarization. Dev. Cell *38*, 384–398.

St Johnston, D., and Ahringer, J. (2010). Cell polarity in eggs and epithelia: parallels and diversity. Cell *141*, 757–774.

Suzuki, A., Hirata, M., Kamimura, K., Maniwa, R., Yamanaka, T., Mizuno, K., Kishikawa, M., Hirose, H., Amano, Y., Izumi, N., et al. (2004). aPKC acts upstream of PAR-1b in both the establishment and maintenance of mammalian epithelial polarity. Curr. Biol. *14*, 1425–1435.

Tabuse, Y., Izumi, Y., Piano, F., Kemphues, K.J., Miwa, J., and Ohno, S. (1998). Atypical protein kinase C cooperates with PAR-3 to establish embryonic polarity in *Caenorhabditis elegans*. Development *125*, 3607–3614.

Wallenfang, M.R., and Seydoux, G. (2000). Polarization of the anterior-posterior axis of *C. elegans* is a microtubule-directed process. Nature *408*, 89–92.

Watts, J.L., Etemad-Moghadam, B., Guo, S., Boyd, L., Draper, B.W., Mello, C.C., Priess, J.R., and Kemphues, K.J. (1996). par-6, a gene involved in the establishment of asymmetry in early *C. elegans* embryos, mediates the asymmetric localization of PAR-3. Development *122*, 3133–3140.

Wirtz-Peitz, F., Nishimura, T., and Knoblich, J.A. (2008). Linking cell cycle to asymmetric division: aurora-A phosphorylates the Par complex to regulate Numb localization. Cell *135*, 161–173.

Yamanaka, T., Horikoshi, Y., Izumi, N., Suzuki, A., Mizuno, K., and Ohno, S. (2006). Lgl mediates apical domain disassembly by suppressing the PAR-3-aPKC-PAR-6 complex to orient apical membrane polarity. J. Cell Sci. *119*, 2107–2118.

Yamanaka, T., Horikoshi, Y., Sugiyama, Y., Ishiyama, C., Suzuki, A., Hirose, T., Iwamatsu, A., Shinohara, A., and Ohno, S. (2003). Mammalian Lgl forms a protein complex with PAR-6 and aPKC independently of PAR-3 to regulate epithelial cell polarity. Curr. Biol. *13*, 734–743.

Ziman, M., O'Brien, J.M., Ouellette, L.A., Church, W.R., and Johnson, D.I. (1991). Mutational analysis of CDC42Sc, a *Saccharomyces cerevisiae* gene that encodes a putative GTP-binding protein involved in the control of cell polarity. Mol. Cell Biol. *11*, 3537–3544.

Ziomek, C.A., Johnson, M.H., and Handyside, A.H. (1982). The developmental potential of mouse 16-cell blastomeres. J. Exp. Zool. *221*, 345–355.

Zipperlen, P., Fraser, A.G., Kamath, R.S., Martinez-Campos, M., and Ahringer, J. (2001). Roles for 147 embryonic lethal genes on *C. elegans* chromosome I identified by RNA interference and video microscopy. EMBO J. *20*, 3984–3992.

STAR★METHODS

KEY RESOURCES TABLE

REAGENT or RESOURCE	SOURCE	IDENTIFIER
Antibodies		
Rabbit Anti-PAR-2	(Dong et al., 2007)	N/A
Rabbit Anti-PAR-6	(Gotta et al., 2001)	N/A
Rat Anti-PKC-3	(Tabuse et al., 1998)	N/A
Mouse Anti-PAR-3	Developmental Studies Hybridoma Bank	P4A1; RRID: AB_528424
Mouse Anti-αTubulin	Sigma	DM1A (T9026); RRID: AB_477593
Rabbit Anti-pLLGL1/2(S650/S654)	Abnova	PAB4657; RRID: AB_1577970
α-rabbit-Alexa488/594/647	Molecular Probes	RRID: AB_2576217 / RRID: AB_2534095 / RRID: AB_2535813
α-mouse-Alexa488/594/647	Molecular Probes	RRID: AB_138404 / RRID: AB_141672 / RRID: AB_141725
α-rat-Alexa488/594/647	Molecular Probes	RRID: AB_141373 / RRID: AB_141374 / RRID: AB_141778
α-mouse-HRP	DAKO	P0447; RRID: AB_2617137
α-rat-HRP	DAKO	P0450; RRID: AB_2630354
Bacterial and Virus Strains		
E. coli: OP50: *E. coli* B, uracil auxotroph	CGC	WB Strain: OP50
E. coli: HT115(DE3): F-, mcrA, mcrB, IN(rrnD-rrnE)1, rnc14::Tn10(DE3 lysogen: lavUV5 promoter-T7 polymerase).	CGC	WB Strain: HT115(DE3)
E. coli : DH5α Electrocompetent cells	Gift from Colin Dolphin	N/A
Chemicals, Peptides, and Recombinant Proteins		
aPKC inhibitor: CRT0103390	Cancer Research Technology LTD	CRT0103390
phorbol 12-myristate 13-acetate (PMA)	Sigma-Aldrich	Cat#P1585-1MG
PKCι-(recombinant human baculovirus-expressed)	EMD Millipore	Cat#14-505
PKCζ-(recombinant active protein, His tagged, Sf21 cells-expressed)	EMD Millipore	Cat#14-525
FAM-PKCε-pseudosubstrate	Molecular Devices	Cat#RP7548
ATP	Sigma-Aldrich	Cat#A7699
Critical Commercial Assays		
IMAP fluorescence polarization progressive binding system	Molecular Devices	#R8127
KINOMEscan	DiscoveRx	N/A
Deposited Data		
CRT0103390 synthesis	Patent	WO/2013/078126
pkc-3(ne4246)	Allele Sequence	*ne4246*
Experimental Models: Cell Lines		
HEK-293	Cell Production, Cancer Research UK (CRUK)	HEK-293
Experimental Models: Organisms/Strains		
C. elegans: N2 (Bristol)	CGC	WB Strain: N2
C. elegans: HT1593: *unc-119*(*ed3*) *III.*	CGC	WB Strain: HT1593
C. elegans: DR466: *him-5*(*e1490*) *V.*	CGC	WB Strain: DR466
C. elegans: DP38: unc-119(ed3) *III*; *daf-?.*	CGC	WB Strain: DP38
C. elegans: JA1643*[gfp*::*wsp-1*; *pkc-3* *(ts)]*: *ojIs40 [Ppie-1*::*gfp*::GBDwsp-1 + *unc-119*(+)*]*;*pkc-3*(*ne4246*)*II*	this paper	JA1643

(*Continued on next page*)

Continued

REAGENT or RESOURCE	SOURCE	IDENTIFIER
C. elegans: JA1644*[gfp::cdc-42; pkc-3(ts)]: unc-119(ed3) III; tjls6 [Ppie-1::gfp::cdc-42 + unc-119(+)]; pkc-3(ne4246)II.*	this paper	JA1644
C. elegans: JH2802*[Dendra2::MEX-5]: unc-119(ed3) III; axIs1950[mex-5p:: Dendra2::TEV::S-peptide::mex-5RR:: mex-5 3'UTR + unc-119(+)]*	CGC	WB Strain: JH2802
C. elegans: *JH2840[mCherry::mex-5]: axIs??? [nmy-2p::pgl-1::GFP::patr-1:: nmy-2 3'UTR]. axIs1731 [pie-1p:: mCherry::mex-5::pie-1 3'UTR + unc-119(+)]*	CGC	WB Strain: JH2840
C. elegans: KK1063*[lgl-1::gfp]: it256 [lgl-1::gfp + unc-119(+)]; unc-119(ed4) III; lgl-1(tm2616) X*	(Beatty et al., 2010)	WB Strain: KK1063
C. elegans: KK114*[par-2(ts)]: daf-7(e1372) par-2(it5) III*	CGC	WB Strain: KK114
C. elegans: KK1216*[par-3::gfp]: par-3(it298 [par-3::gfp]) III*	Ken Kemphues / CGC	WB Strain: KK1216
C. elegans: KK1228*[gfp::pkc-3]: pkc-3(it309 [gfp::pkc-3]) II*	Liam Coyne<br>Ken Kemphues / CGC	WB Strain: KK1228
C. elegans: KK1248*[par-6::gfp]: par-6(it310[par-6::gfp]) I*	Anushae Syed<br>Ken Kemphues / CGC	WB Strain: KK1248
C. elegans: KK1262*[par-1::gfp]: par-1 (it324[par-1::gfp::par-1 exon 11a])*	Diane Morton / CGC	WB Strain: KK1262
C. elegans: KK822*[par-1(ts)]: par-1(zu310) V*	CGC	WB Strain: KK822
C. elegans: KK973*[par-3:Δcr1:gfp]: itIs169 [Ppar-3::par-3 CR1 Δ(69-82):::gfp, unc-119(+)]; unc-119(ed4) III*	Ken Kemphues	KK973
C. elegans: *NWG0003[par-2(ts); gfp:: par-6]: daf-7(e1372) par-2(it5) III; unc-119 (ed3) III; ddIs8 [gfp::par-6(cDNA); unc-119(+)]*	this paper	NWG0003
C. elegans: *NWG0012[gfp::c1b]: unc-119(ed3)III; crkIs4[Ppie-1::sfgfp:: c1b::pie-1utr; unc-119(+)]*	this paper	NWG0012
C. elegans: *NWG0016[gfp::c1b::pkc-3]: unc-119(ed3)III; crkIs10[Ppie-1::sfgfp:: c1b::pkc-3::pie-1utr; unc-119(+)]*	this paper	NWG0016
C. elegans: *NWG0021[gfp::c1b::pkc-3; mCherry::par-2]:unc-119(ed3)III; ddIs31[pie-1p::mCherry::par-2::pie-1utr; unc-119(+)]; crkIs10[Ppie-1::sfgfp::c1b:: pkc-3::pie-1utr; unc-119(+)]*	this paper	NWG0021
C. elegans: *NWG0022[gfp::c1b;mCherry:: par-2]: unc-119(ed3)III; ddIs31[pie-1p:: mCherry::par-2::pie-1utr; unc-119(+)]; crkIs4[Ppie-1::sfgfp::c1b::pie-1utr; unc-119(+)]*	this paper	NWG0022
C. elegans: *NWG0026[par-6::gfp; mCherry:: par-2]: par-6(it310[par-6::gfp]) I;unc-119 (ed3)III; ddIs31[pie-1p::mCherry::par-2; unc-119(+)]*	this paper	NWG0026
C. elegans: *NWG0027[gfp::pkc-3; mCherry:: par-2]: pkc-3(it309 [gfp::pkc-3]) II;unc-119(ed3)III; ddIs31[pie-1p::mCherry::par-2; unc-119(+)]*	this paper	NWG0027

(*Continued on next page*)

Continued

REAGENT or RESOURCE	SOURCE	IDENTIFIER
C. elegans: *NWG0028[par-3::gfp; mCherry::par-6]: par-3(it298 [par-3::gfp]) III;unc-119(ed3)III;ddIs26 [mCherry::T26E3.3;unc-199(+)]*	this paper	NWG0028
C. elegans: *NWG0039[par-1(ts); par-6::gfp]: par-1(zu310) V; par-6(it310[par-6::gfp]) I.*	this paper	NWG0039
C. elegans: *NWG0047*[PH-GBP]: *unc-119(ed3) III; crkEx1[pNG19: mex-5p::PH(PLC1Δ1)::GBP::mKate::nmy-2UTR + unc-119(+)]; him-5 (e1490) V.*	this paper	NWG0047
C. elegans: *NWG0053[par-6::gfp;pkc-3(ts)]: par-6(it310[par-6::gfp]) I; pkc-3(ne4246)II*	this paper	NWG0053
C. elegans: *OD70[mCherry::PH-PLCΔ1]: unc-119(ed3) III; ltIs44[pie-1p-mCherry:: PH(PLC1Δ1) +unc-119(+)] V*	(Kachur et al., 2008)	WB Strain: OD70
C. elegans: *SA131[gfp::cdc-42]: unc-119(ed3)III; tjIs 6[Ppie-1::gfp:: cdc-42+unc-119(+)]*	(Motegi and Sugimoto, 2006)	WB Strain: SA131
C. elegans: *TH129[gfp::par-2]: unc-119 (ed3)III;ddIs25[GFP::F58B6.3;unc-119(+)];*	(Schonegg et al., 2007)	TH129
C. elegans: *TH159[mCherry-cdc-42]*: unc-119(ed3)III; ddIs46[WRM0625bA11 GLCherry::cdc-42; Cbr-unc-119(+)]	Tony Hyman	TH159
C. elegans: *TH209[mCherry::par-2]: unc-119(ed3)III; ddIs31[pie-1p::mCherry:: par-2; unc-119(+)]*	(Schonegg et al., 2007)	TH209
C. elegans: *TY3558[gfp::his-11; b-tubulin:: gfp]: ruIs[pie-1::GFPhis-11] III; ojIs1 [β-tubulin::GFP]*	CGC	WB Strain: TY3558
C. elegans: *UE37[pie-1::gfp]: axEx73 [pie-1p::pie-1::GFP + rol-6(su1006) + N2 genomic DNA]; tubulin mCherry*	Carrie Cowan	UE37
C. elegans: *WH423[mCherry::cdc-42(Q61L)]: Ppie-1::mcherry::cdc-42(Q61L)*	(Kumfer et al., 2010)	WH423
C. elegans: *WH497[gfp::chin-1]: ojIs69 [pie-1::mGFP::chin-1 + unc-119(+)]*	CGC	WB Strain: WH497
C. elegans: *WH517[gfp::wsp-1]: ojIs40 [Ppie-1::gfp::GBDwsp-1 + unc-119(+)]*	CGC	WB Strain: WH517
C. elegans: *WM150[pkc-3(ts)]: pkc-3(ne4246) II*	(Fievet et al., 2012)	WM150
C. elegans: *WS5018[gfp::cdc-42]: cdc-42(gk388); opIs295 [cdc-42p::gfp:: cdc-42(genomic)::cdc-42 3'UTR + unc-119(+)] II.*	(Neukomm et al., 2014)	WB Strain: WS5018
**Oligonucleotides**		
pkc-3(genomic) fwd:CCCACTAGTATGTCG TCTCCGACAT (SpeI)	IDT DNA	N/A
pkc-3(genomic) rev:CCCAGGCCTTCAGAC TGAATCTTCC (StuI)	IDT DNA	N/A
PH-GBP gBlock: fwd: TTCCGTTTTCTCATTGTATTCTCTC	IDT DNA	N/A
PH-GBP gBlock: rev: ATGATGCCGGCTTAGCTAGC	IDT DNA	N/A
Site-directed mutagenesis (PAM site) in pNG0018, fwd: GTCTGTTTCGTAACTGTCTTCTGTATAACT	IDT DNA	N/A

(*Continued on next page*)

Continued

REAGENT or RESOURCE	SOURCE	IDENTIFIER
Site-directed mutagenesis (PAM site) in pNG0018, fwd: TGATATCGAAACAAACACTG	IDT DNA	N/A
ctl (RNAi): fwd: ATCGATAAGCTTTGTATCCTCTTG	IDT DNA	N/A
ctl (RNAi): rev: ACCGGCGGATCCTTAAATACGG	IDT DNA	N/A
Recombinant DNA		
Fosmid: WRM069dD11	Source BioScience	WB Clone: WRM069dD11
Plasmid: L4440	Addgene	plasmid#1654
Plasmid: pUC57-C1B(codon-optimized)	GenScript	N/A
Plasmid: pTH699	Gift from Tony Hyman	N/A
Plasmid: pC1B-Ø	This paper	N/A
Plasmid: pC1B-pkc-3	This paper	N/A
Plasmid: CmKate2 MosSci vector	Gift from Tony Hyman	N/A
Plasmid: pNG0018	This paper	N/A
Plasmid: pNG0019	This paper	N/A
Ahringer Feeding RNAi: *cdc-42*	Source BioScience	WB Clone: sjj_R07G3.1
Ahringer Feeding RNAi: emb-27	Source BioScience	WB Clone: sjj_F10B5.6
Ahringer Feeding RNAi: *par-3*	Source BioScience	WB Clone: sjj_F54E7.3
Ahringer Feeding RNAi: *par-6*	Source BioScience	WB Clone: sjj_T26E3.3
Ahringer Feeding RNAi: *pkc-3*	Source BioScience	WB Clone: sjj_F09E5.1
Ahringer Feeding RNAi: *perm-1*	Source BioScience	WB Clone: sjj_T01H3.4
Ahringer Feeding RNAi: *rga-3*	Source BioScience	WB Clone: sjj_K09H11.3
Ahringer Feeding RNAi: *cgef-1*	Source BioScience	WB Clone: sjj_C14A11.3
Feeding RNAi: *mlc-4*	(Redemann et al., 2010)	N/A
Feeding RNAi: control(ctl)	This paper	N/A
PH-GBP gBlock (sequence on request)	IDT DNA	N/A
ctl (RNAi): gBlock (sequence on request)	IDT DNA	N/A
Software and Algorithms		
Matlab	Mathworks	R2016a
Kilfoil Feature Tracking (feature2D.m)	http://people.umass.edu/kilfoil/downloads.html	N/A
Fiji (ImageJ)	https://fiji.sc/#	N/A
ActivityBase	IDBS	N/A

CONTACT FOR REAGENT AND RESOURCE SHARING

Requests for resources and reagents should be directed to and will be fulfilled by the Lead Contact, Josana Rodriguez (josana.rodriguez@ncl.ac.uk). CRT0103390 may be obtained through an MTA from Cancer Research Technology (jroffey@cancertechnology.com).

EXPERIMENTAL MODEL AND SUBJECT DETAILS

C. elegans Strains and Maintenance

C. elegans strains were maintained on nematode growth media (NGM) under standard conditions (Brenner, 1974) at 16°C or 20°C unless otherwise indicated. Strains listed in the Key Resources Table. Note analysis of zygotes precludes determination of animal sex.

C. elegans Transgenic Animals

Following the scheme of (Lekomtsev et al., 2012), a codon-optimized (Redemann et al., 2011) sequence encoding the C1B domain from human PKCα (GenScript) was inserted into pTH699 via BamHI and SmaI to generate a *sfgfp::c1b* fusion under control of the *pie-1* promoter and -3' UTR (pC1B-Ø). Genomic *pkc-3* was amplified from fosmid WRM069dD11 (Source BioScience, WB Clone: WRM069dD11) using the following primers (fwd:cccactagtatgtcgtctccgacat; rev:cccaggccttcagactgaatcttcc) and inserted into

(pC1B-Ø) using SpeI and StuI to generate pC1B-pkc-3. Both plasmids were introduced by biolistic bombardment into HT1593 worms (Praitis et al., 2001), yielding NWG0012 and NWG0016.

The membrane-tethered GFP-binding protein (PH-GBP) was generated by combining amino acids 1-175 corresponding to the PH domain of rat PH-PLCΔ1 (Audhya et al., 2005) and VHH4GFP (Caussinus et al., 2011) coupled by a SGQGGSGGSGGS linker. The resulting sequence was codon-optimized (CAI = 0.49) and a single GFP intron inserted as described (Redemann et al., 2011). A synthetic gBlock (IDT DNA) encoding the PH-GBP was PCR amplified and cloned in frame with a C-terminal codon-optimized mKate2 under the control of the *mex-5* promoter and *nmy-2* 3' UTR in a MosSCI vector containing wild-type *unc-119* obtained from the Hyman Lab. The resulting plasmid (pNG0018) was inserted at the ttTi5605 *mos1* site locus of DP38 worms via CRISPR after mutating the sgRNA/PAM site following the method described (pNG0019) (Dickinson et al., 2013). Modified worms were crossed into DR466 to generate a stable male line expressing PH-GBP (NWG0047). To rescue membrane localization of PAR-3 variants, we crossed NWG0047 with KK1216 (*par-3*::*gfp*) or KK973 (*par-3Δcr1*::*gfp*) lines. We were unable to obtain a stable homozygous line for the endogenously tagged PAR-3::GFP, presumably due to the toxic effects of continuously targeting all PAR-3 to the membrane throughout embryogenesis. Thus, we used F1 progeny heterozygous for PAR-3::GFP for analysis. By contrast, for PAR-3ΔCR1::GFP, which is expressed ectopically from a multi-copy random insertion, we readily obtained animals homozygous for both PAR-3ΔCR1::GFP and PH-GBP, which were used for subsequent analysis. However, no significant difference in the segregation of PAR-3ΔCR1::GFP was seen between heterozygous and homozygous animals.

For analysis of GFP::CDC-42, SA131 was used unless otherwise indicated.

For analysis of the effects of CDC-42(Q61L) on GFP::PKC-3 localization, zygotes were taken from F1 animals resulting from crossing KK1228 with either TH159 or WH423 due to difficulties obtaining stable animals homozygous for both markers.

Cell Lines

HEK-293 are female and were obtained from Cell Production, Cancer Research UK (CRUK) and cultivated in DMEM (Dulbecco's modified Eagle's medium), 10% FBS (fetal bovine serum) and penicillin–streptomycin (Invitrogen) (Kjær et al., 2013).

Bacterial Strains

OP50 bacteria and HT115(DE3) were obtained from CGC. DH5α was obtained from Colin Dolphin. Feeding by RNAi used HT115(DE3) bacteria strains containing a plasmid carrying the indicated RNAi feeding plasmid.

METHOD DETAILS

C. elegans - RNAi Culture Conditions

RNAi by feeding was performed similar to described methods (Kamath et al., 2003). Briefly, HT115(DE3) bacterial feeding clones were inoculated from LB agar plates to LB liquid cultures and grown overnight at 37°C in the presence of 10 μg/ml carbenicillin. Bacterial cultures were induced with 5 mM IPTG at 37° for 4h with agitation before spotting 100 μl of induced bacteria onto 60 mm agar RNAi plates (10 μg/ml carbenicillin, 1 mM IPTG). L4 larva were added to RNAi feeding plates and incubated for 24-72 hr depending on gene and temperature. For temperature sensitive lines, feeding was performed at 15°C for 48-72 hr and shifted to 25°C for 2-5 hr before imaging. For double depletion experiments, L3/L4 larva carrying *par-1*(*zu310*) or *par-2*(*it5*) temperature sensitive mutants were placed on RNAi plates at 15°C for 24 hr before a fraction of those were moved to fresh RNAi expressing plates for 18 to 22 hr at 25°C. Partial RNAi for *mlc-4* was performed for 14-24 hr at 20°C. For partial depletion of *perm-1*, *rga-3/4* or *pkc-3*, bacteria expressing the desired clone were mixed at the indicated ratios with bacteria expressing control RNAi. *par-3*, *par-6*, *cdc-42*, *pkc-3*, *perm-1*, rga-3/4, *cgef-1* and *emb-27* clones are from the Ahringer library (Kamath et al., 2003). *mlc-4* is from Redemann et al., 2010. A control RNAi clone was generated by synthesizing a random 500bp sequence using the Matlab random number generator with no homology to the worm genome, cloned into Bgl-II / HindIII sites of L4440 (Addgene, plasmid#1654), and transformed into HT115 bacteria.

C. elegans Embryos – Western Blots

Embryos were obtained by a standard bleaching protocol and resuspended in NuPAGE LDS sample buffer (Invitrogen) prior to sonication using the Biorupture (Diagenode) for 5 - 30 s on, 30 s off cycles. Samples were heated at 70°C for 10 min before centrifugation at 13.000 rpm for 20 min to obtain cleared supernatant. Samples were run on a 12% NuPAGE gel using MOPS SDS running buffer (Invitrogen) and transferred onto PVDF membrane (Immobilon-P membrane 0.45 um, Millipore). PKC-3 and tubulin was detected using the primary (anti-PKC-3 1:10.000 and anti-tubulin 1:20.000) and secondary antibodies (as recommended by provider) indicated in Key Resources Table and detected via chemiluminescence (ECL prime, GE Healthcare Life Sciences). PKC-3 band intensity was analyzed using the Fiji Gel analysis tool.

C. elegans Zygotes – Drug Treatment

All drug treatment experiments were performed in 10 to 50% *perm-1*(*RNAi*) (Carvalho et al., 2011). Drugs were dissolved in DMSO and used at the following concentrations: phorbol 12-myristate 13-acetate (PMA, Sigma-Aldrich, P1585-1MG), 100 μM; CRT90 (CRT0103390, Cancer Research Technology LTD), 10 μM. When drug treatment alone was required, we obtained zygotes with permeable eggshells by placing L4 animals on a 1:1 mix of bacteria expressing *perm-1*(*RNAi*) and *ctl* (*RNAi*) for 16 to 20 hr at

20°C. When drug treatment was combined with additional RNAi treatment, L4 animals were placed on bacteria expressing *perm-1*(*RNAi*) mixed at a 1:9 ratio with bacteria expressing the desired RNAi (*par-3*, *par-6*, *cdc-42*, *control*) and incubated for 40-48 hr at 20°C.

C. elegans Zygotes - Immunofluorescence

Immunofluorescence was performed as previously described (Andrews and Ahringer, 2007). Briefly, gravid hermaphrodite worms were washed and then transferred to a 7 μl drop of M9 on a 0.1% poly-lysine coated well. Embryos were released using a needle and then covered with a coverslip to compress the embryos. Slides were snap-frozen on dry ice for 30 min after which the coverslip was quickly removed and the slide fixed in methanol at room temperature for 20 min. Samples were washed and re-hydrated with PBS followed by two 10 min washes in PBS+0.2% Tween-20 before proceeding with antibody incubations, DAPI staining and mounting in Mowiol (Sigma-Aldrich). All antibodies used in this study are listed in Key Resources Table. Primary antibody dilutions used: anti-PAR-2 1:500, anti-PAR-6 1:10, anti-PKC-3 1:500 and anti-PAR-3 1:50. Secondary antibodies were used as recommended by provider. Confocal images were acquired using Carl Zeiss Axioplan 2, LSM510 Meta confocal equipped with LSM image software and Nikon A1R equipped with Nikon elements software and a 63× objective. Cortical super-resolution images were acquired using the DeltaVision OMX system equipped with SoftWoRx and OMX acquisition software. Secondary processing of images was performed using Photoshop CS5 and Illustrator CS5 (Adobe).

C. elegans zygotes - Live Imaging

Embryos were dissected in 2-4 μl of M9 buffer (22 mM KH_2PO_4, 42 mM $NaHPO_4$, 86 mM NaCl and 1 mM $MgSO_4$) on a coverslip and mounted under 2% agarose pads (Zipperlen et al., 2001) or dissected in Shelton's Growth Medium (Edgar and Goldstein, 2012) and mounted with 16-21 μm polystyrene beads between a slide and coverslip and sealed with VALAP (Goehring et al., 2011a). 16-18 μm beads were used for cortex imaging to maximize imaging surface. In all other cases, 21 μm beads were used to minimize compression effects on development. For CRT90 experiments, embryos were dissected in the presence of 10 μM CRT90. For C1B targeting experiments, two sides of the coverslip were left unsealed to create a flow chamber (Goehring et al., 2011a) and PMA washed in at the indicated times.

To maximize viability, embryos were typically imaged at 20-22°C, except for temperature sensitive alleles, which were imaged at the indicated temperatures using an objective temperature control collar (Bioptechs / Linkam, PE94). For consistency, establishment phase embryos were taken at pronuclear meeting, and maintenance phase was defined as the interval from nuclear envelope breakdown to metaphase.

Cortex images were captured with a 100x 1.49 NA TIRF objective on a Nikon TiE (Nikon) equipped with an iLas2 TIRF unit (Roper), 488 or 561 fiber coupled diode lasers (Obis), and an Evolve Delta camera (Photometrics). Midplane imaging was performed on Carl Zeiss Axioplan 2, LSM510 Meta confocal or a Nikon TiE with 63x or 100x objectives, further equipped with either a Spectra-X LED light source (wide-field) or a custom X-Light V1 spinning disk system (CrestOptics, S.p.A.) with 50μm slits, 488, 561 fiber-coupled diode lasers (Obis) and either a CoolSnap HQ or Evolve Delta (Photometrics). Imaging systems were run using Metamorph (Molecular Devices) and configured by Cairn Research (Kent, UK).

In vitro PKC Enzyme Assays

The ability of compounds to inhibit the kinase activity of recombinant human baculovirus-expressed full-length PKCι was measured using the IMAP fluorescence polarization (FP) progressive binding system (Molecular Devices #R8127, Sunnyvale, CA) in 384-well black, non-binding, flat-bottom assay plates (Corning #3575, Corning, NY). The assay mixture (final volume = 10 μL) contained 20 mM Tris-HCL (pH 7.5), 150 μM ATP, 10 mM MgCl2, 0.01% Triton X-100, 250 μM EGTA, 1 mM DTT, 15 pM PKCι (EMD Millipore #14-505, Billerica, MA), 100 nM FAM-PKCε-pseudosubstrate (Molecular Devices #RP7548), 0.1% DMSO and various concentrations of test compound. Compound dilutions (prepared in 100% DMSO) were added to the assay plate at 100 nL using the BioMek NX pin tool (Beckman Coulter, Indianapolis, IN). Enzyme reactions were initiated by the addition of ATP (Sigma- Aldrich #A7699, St. Louis, MO), followed by incubation of the plates for 1 hour in a 25°C incubator. A 20 μL aliquot of IMAP detection reagent (1:400 in 85% 1X Binding Buffer A and 15% 1X Binding Buffer B) was added to each well followed by a 2-hour incubation at 25°C. Fluorescence polarization was then measured using the PerkinElmer Envision 2102 multi-label plate reader (PerkinElmer, Waltham, MA) using the FP dual mirror, FP480 excitation filter and P-pol 535 and S-pol 535 emission filters. Data analysis was performed using ActivityBase (IDBS, Guilford, UK). IC_{50} values were calculated by plotting percent inhibition versus log10 of the concentration of compound and fitting to the 4-parameter logistic model (top and bottom constrained to 100 and 0, respectively) in XLFit 4 (IDBS).

The PKCζ kinase assay was performed using the IMAP FP progressive binding system as described above for PKCι but with some modifications. The concentration of PKCζ (recombinant active protein, His tagged, expressed in Sf21 cells, Millipore, 14-525) was 10pM, while the substrate concentrations were 100 nM and 40 μM for the FAM-PKCε-pseudosubstrate (Molecular Devices #RP7548) and ATP, respectively.

Cellular Biochemical Assay

HEK-293 cells were transfected in a 10 cm dish as per the manufacturer's instructions (Corning). After 16 hr, the cells were trypsinized and seeded into a 96-well plate at 1.5×10^4 cells/well and medium was replenished. After a further 24 hr, the medium was replaced by new medium and a range of CRT0103390 inhibitor concentrations. After 1 hr of inhibitor treatment, lysates were prepared using

ice-cold Tris lysis buffer [150 mM NaCl, 20 mM Tris (pH 7.5), 1 mM EGTA, 1 mM EDTA and 1% Triton X-100]. Lysates were transferred on to an anti-FLAG-coated ELISA plate (Sigma) and incubated for 2 hr with gentle shaking, followed by an automated wash step (Tecan plate washer) with wash buffer [50 mM Tris (pH 7.5), 0.15 M NaCl and 0.02% Tween 20]. The immunocomplexed protein was incubated with anti-pLLGL1/2 (S650/S654) primary antibody overnight at 4°C, followed by an automated wash and then addition of HRP-conjugated secondary antibody. After a further wash, 3,3',5,5'-tetramethylbenzidine (Sigma) was added according to the manufacturer's instructions and attenuance was read at 450 nm using an Ascent plate reader (Thermo Labsystems).

Kinase Selectivity

CRT0103390 was profiled using the KINOMEscan *in vitro* competition binding screening platform at DiscoveRx against a panel of 442 mutant and non-mutant kinases at a test concentration of 1 μM. Selectivity scores were calculated as the number of non-mutant kinases with % activity relative to control < 20/number of non-mutant kinases tested. CRT0103390 demonstrated a high degree of selectivity in this panel, with an S(80) of 0.09.

QUANTIFICATION AND STATISTICAL ANALYSIS

Image Analysis - General

All image analysis was performed in Fiji (ImageJ)(Schindelin et al., 2012) and Matlab (Mathworks).

Image Analysis - Asymmetry Index (ASI)

The asymmetry index (ASI) of a feature is defined by:

$$\frac{A-P}{2(A+P)}$$

where A and P define the anterior and posterior signal, respectively. Raw ASI values are normalized to the mean ASI observed in respective controls, such that a value of 1 indicates wild-type asymmetry and zero indicates complete loss of asymmetry. Anterior and posterior signals are defined depending on the condition examined and include cross-sectional area (AB vs P1 asymmetry), fluorescence intensity on the two cell halves for the membrane (midsection PAR analysis) or cytoplasm (MEX-5, PIE-1), or cluster number (CHIN-1).

Image Analysis – Cluster Index

The Cluster Index is defined as the variance in cortical intensity within the anterior domain. It was calculated in Matlab across user specified ROIs that were subject to background subtraction and normalization to mean intensity before analysis.

Image Analysis – CHIN-1 Foci

CHIN-1 foci were identified using the feature2D.m script, part of the feature detection and particle tracking package from the Kilfoil Lab (Pelletier et al., 2009). Embryos were automatically detected and partitioned into 3 domains (Anterior, Middle, Posterior) and normalized anterior vs posterior particle densities used for ASI calculation.

Image Analysis – MEX-5 Mobility

For MEX-5 mobility, five pre-bleach frames were captured by spinning disk confocal microscopy. A central 20-pixel wide stripe was then bleached along the AP axis using a 473 diode laser (Obis) and recovery was monitored every 2 s. Because MEX-5 is uniform in the quantified conditions, fluorescence was monitored within a central 20 x 100-pixel box.

Image Analysis - Colocalization

Colocalization analysis of PAR-6 and PAR-3 was performed in ROIs at the anterior cortex of establishment phase zygotes (wild-type n=8 and *pkc-3*(*ts*) n=9). Costes' Mask and intensity correlation quotient (Li et al., 2004) were obtained using JaCOP plug-in in Fiji.

Image Analysis – Flow Speeds

Anterior-directed cortical flow speed during establishment phase was measured using midplane, brightfield images acquired every second until late establishment phase at which point we switched to fluorescent imaging to obtain suitable images for measuring PAR asymmetry. Using the Kymograph Plugin in Fiji (Seitz and Surrey, 2006), we generated kymographs for individual embryos by tracing a segmented line along the cortex starting at the origin of flow. A minimum of 10 yolk granule trajectories, each spanning approximately 200s were selected for a minimum of 5 embryos per condition. The cortical flow velocity was defined as the total distance over time calculated from a line connecting the start and end positions of the granule on the kymograph. Measurement of cortical flow in wild-type embryos expressing GFP fusions to PAR-3, PKC-3 or CDC-42 yielded a velocity of 7.1+/-1.4 μm/min (n=22), consistent with previously published values (7.66+/-1.0 μm/min, n=6)(Munro et al., 2004).

Image Analysis – Anterior Cortical Intensity (Immunofluorescence)

Anterior cortical intensity of each PAR protein is the mean greyscale value of a line 2 pixels wide (in each corresponding fluorescent channel) covering the PAR-3 cortical domain of the zygote. The cortical intensity value is then normalized by dividing it by the mean greyscale value of a nearby cytoplasmic region to correct for embryo IF staining variability. In *par-3*(*RNAi*) zygotes, we cannot distinguish anterior from posterior cortex and the entire cortex of zygotes is analyzed. Each experimental condition was analyzed in three independent experiments.

Image Analysis – Anterior PAR Retraction (Immunofluorescence)

The posterior boundary of anterior PARs in fixed, midsection fluorescent images is defined at the intersection between the equatorial zygote line (longest line linking the zygotes' poles) and the line that links the cortical ends (top and bottom) of the PAR protein analyzed. Retraction is the distance between this posterior boundary and the posterior pole of the zygote. Retraction difference is defined as the difference in retraction distance between PAR-3 and PKC-3. Data were collected from three independent experiments.

Image Analysis – Intensity Profile Extraction

In general, to assess PAR signal from midsection images, a 60-pixel wide stripe encompassing the cell membrane was extracted and straightened to generate a profile for each embryo for further analysis.

For spatial analysis (ASI, profile plots, domain size, segregation efficiency), the top 4 central pixels corresponding to the membrane were taken at each x-position and averaged to the given local membrane signal. Background and cytoplasmic signal were calculated locally from inner and outer edges of this stripe, allowing normalization for variation in signal between conditions. Briefly, background was subtracted and then membrane divided by cytoplasmic intensity.

Image Analysis – PAR-3 versus PKC-3 Cortical Profile Comparison (Immunofluorescence)

To quantitatively compare PAR cortical profiles in multi-labelled fixed embryos, two identical profiles along the cortex were extracted in each channel as above. Each set of profiles was split in half to generate two boundary regions. After normalization to maximum and minimum values, profiles were registered using the inflection point, c, based on fitting each PAR-3 profile using the following function:

$$I(x) = a + \frac{b}{2}(erf(mx - c))$$

where erf is the error function, c is the domain boundary position, m the boundary slope and a and b allow for scaling and displacement on the y-axis.

Image Analysis – Domain Size/Segregation Efficiency

To extract domain size data (PAR-2 domain size change, segregation efficiency) from single channel images, cortical fluorescence profiles were normalized to total embryo perimeter length and aligned to the center of posterior PAR domain determined by fitting the profile to the following function:

$$I(x) = a \pm \frac{b}{2}(erf(mx - c_1) - erf(mx - c_2))$$

with the center of the posterior domain specified by:

$$\frac{c_2 - c_1}{2}$$

posterior domain size given by:

$$\frac{c_2 - c_1}{L}$$

and anterior domain size given by:

$$1 - \frac{c_2 - c_1}{L}$$

where L is the length of the profile.

PAR-2 domain size change for each embryo was calculated as the ratio of domain size taken from images before and 5 min after PMA addition. Segregation efficiency into the anterior was scored by relative anterior domain size, with smaller anterior domains defined as more efficient segregation.

Image Analysis – Total Membrane Signal Change

To estimate total membrane signal in PAR-6 and PKC-3 rescue experiments straightened profiles were projected in x to give a cross-section profile spanning background, crossing the membrane and into the cytoplasm for the full circumference. Cross-section profiles were then normalized with background = 0 and cytoplasm = 1. To get the most accurate estimate of isolated membrane signal,

we generated a mean cross-section for embryos with no detectable membrane signal from *par-3* and *par-6* RNAi embryos to define the shape of the outside to inside fluorescence step. The shape of this curve was extremely consistent allowing a mean profile to be generated, which could then be subtracted from individual embryo cross-section profiles with the sum of the difference taken as membrane signal.

For PAR-2 retention in C1B-induced PKC-3 membrane-targeting experiments, a cytoplasm-normalized 4-pixel wide stripe encompassing the cell membrane was taken from images before and 5 min after PMA addition to generate profiles. PAR-2 retention was defined as the ratio of total membrane signal before and after PMA addition.

Statistics

For all assays, significance was assessed using an unpaired, two-tail Student's T test unless otherwise noted with the following criteria: *p<0.05, **p<0.01, ***p<0.001, ****p<0.0001. Data are presented as mean values plus all data points or mean ± 95% confidence interval (CI), in which case (N) is indicated.

Data and Software Availability

Sequence data for *pkc-3(ne4246)* has been submitted to Wormbase (WB Gene: *pkc-3*).

Developmental Cell, Volume *42*

Supplemental Information

aPKC Cycles between Functionally Distinct PAR Protein Assemblies to Drive Cell Polarity

Josana Rodriguez, Florent Peglion, Jack Martin, Lars Hubatsch, Jacob Reich, Nisha Hirani, Alicia G. Gubieda, Jon Roffey, Artur Ribeiro Fernandes, Daniel St Johnston, Julie Ahringer, and Nathan W. Goehring

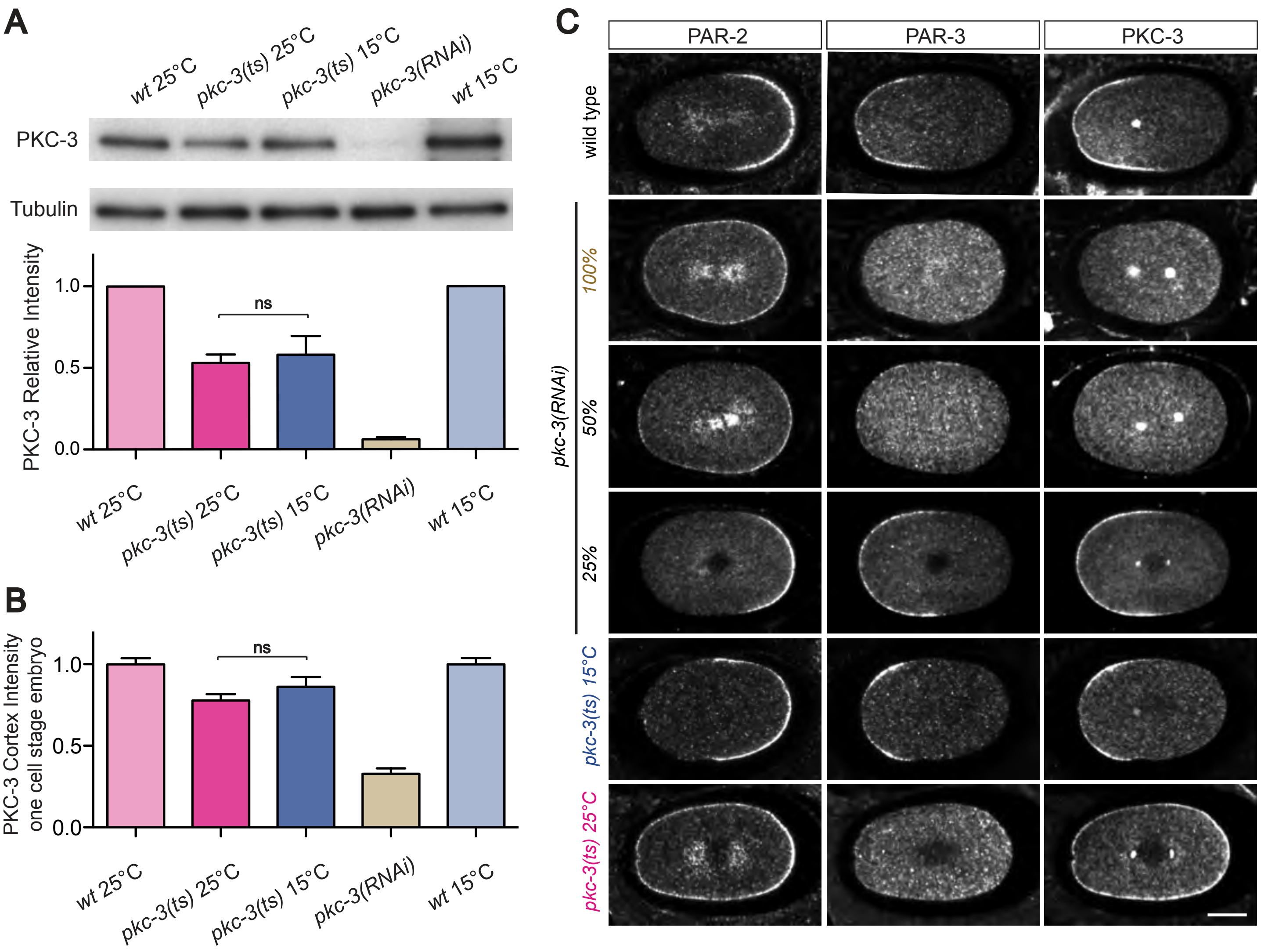

Figure S1. PKC-3 (D386V) causes a temperature sensitive polarity phenotype distinct from reduction in PKC-3 levels, Related to Figure 1

(A) Embryo extract blots at the indicated conditions showing PKC-3 and α-tubulin (loading control). Intensity of PKC-3 (loading-corrected) in *pkc-3(ts)*(D386V) and *pkc-3(RNAi)* embryos quantified relative to their corresponding wild type temperature condition (mean±SEM). *pkc-3(RNAi)* was performed at 25ºC. Note that *pkc-3(ts)* at the restrictive and permissive temperatures show similar reduction in PKC-3 protein amounts compared to controls. *pkc-3(ts)* 25ºC (n=5), *pkc-3(ts)* 15ºC (n=3), *pkc-3(RNAi)* (n= 4).

(B) Quantification of zygote PKC-3 cortical intensity determined by confocal immunofluorescence. PKC-3 cortex intensity of *pkc-3(ts)* embryos are plotted relative to PKC-3 cortical levels of control zygotes (mean±SEM). Control (TY3558) and *pkc-3(ts)* (WM150) zygotes were processed on the same slide to facilitate direct comparisons, with control zygotes identifiable by expression of GFP fusions to histone and β-tubulin. Embryos subjected to *pkc-3(RNAi)* were mounted on a separate slide but processed in parallel and imaged under identical conditions. Note that the results are broadly similar to what is seen in western blots. wt 25ºC (n=23), *pkc-3(ts)* 25ºC (n=31), *pkc-3(ts)* 15ºC (n=25), *pkc-3(RNAi)* (n= 10) and wt 15ºC (n=27).

(C) Representative midsection confocal images of wild type, *pkc-3(RNAi)* and *pkc-3(ts)* metaphase zygotes, immunostained for PAR-2, PAR-3 and PKC-3. Note that although PKC-3 levels are similar in *pkc-3(ts)* at 15ºC and 25ºC (A and B), only *pkc-3(ts)* at 25ºC shows a clear polarity defect. *pkc-3(ts)* at 15ºC appears very similar to the phenotype observed under a weak knock down of PKC-3 (*pkc-3(RNAi)* 25%), including the appearance of a small, transient anterior PAR-2 domain during the establishment phase (data not shown). Scale bar: 10μm.

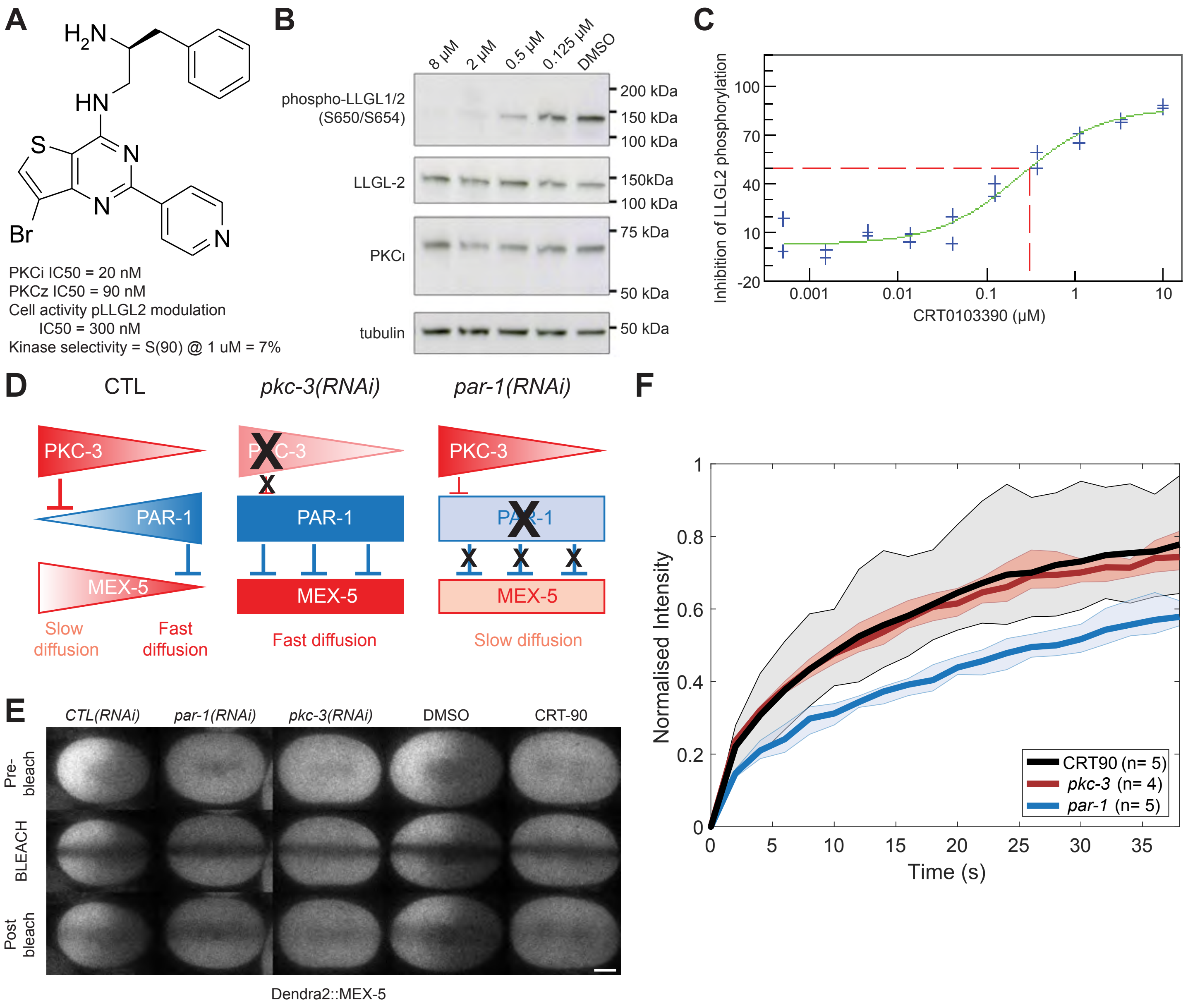

Figure S2. CRT0103390 inhibits atypical PKC without affecting PAR-1, Related to Figure 1

(A) Structure of CRT0103390 and key enzymatic data. Kinase selectivity represents the percent of kinases out of a panel of 442 diverse kinases inhibited by >90%.

(B) CRT0103390 inhibits LLGL2 phosphorylation in HEK-293 cells, co-transfected with LLGL2-FLAG and PKCι, after 1 hr of treatment as assayed by immunoblot analysis.

(C) IC50 curve of LLGL2 phosphorylation by CRT0103390 measured by ELISA.

(D) The diffusive state of MEX-5 along the antero-posterior zygote axis is regulated by PAR-1, which is epistatic to PKC-3. In *pkc-3(RNAi)* embryos, PAR-1 is uniformly active, resulting in uniform, fast diffusion of MEX-5. Conversely, in *par-1(RNAi)* or *par-1/pkc-3(RNAi)* embryos, MEX-5 is uniformly slow (Griffin et al., 2011). If CRT90 inhibited both PAR-1 and PKC-3, we would expect uniform slow MEX-5, which we do not observe in (E) and (F).

(E) Selected midsection confocal images of MEX-5 in control, *par-1(RNAi)*, *pkc-3(RNAi)*, DMSO and CRT90 treated zygotes, before (Pre-bleach), immediately after (BLEACH), or 20 seconds after fluorescence photobleaching of a central stripe along the AP axis (Post-bleach). Scale bar: 10μm.

(F) Pre-bleach-normalized fluorescence intensity recovery of a central region of the embryo, showing that MEX-5 mobility in CRT90-treated embryos closely matches the faster recovery of *pkc-3(RNAi)* embryos compared to *par-1*(*RNAi*). For each condition, mean values are shown (thick line) along with a shaded region indicating the full data range across samples.

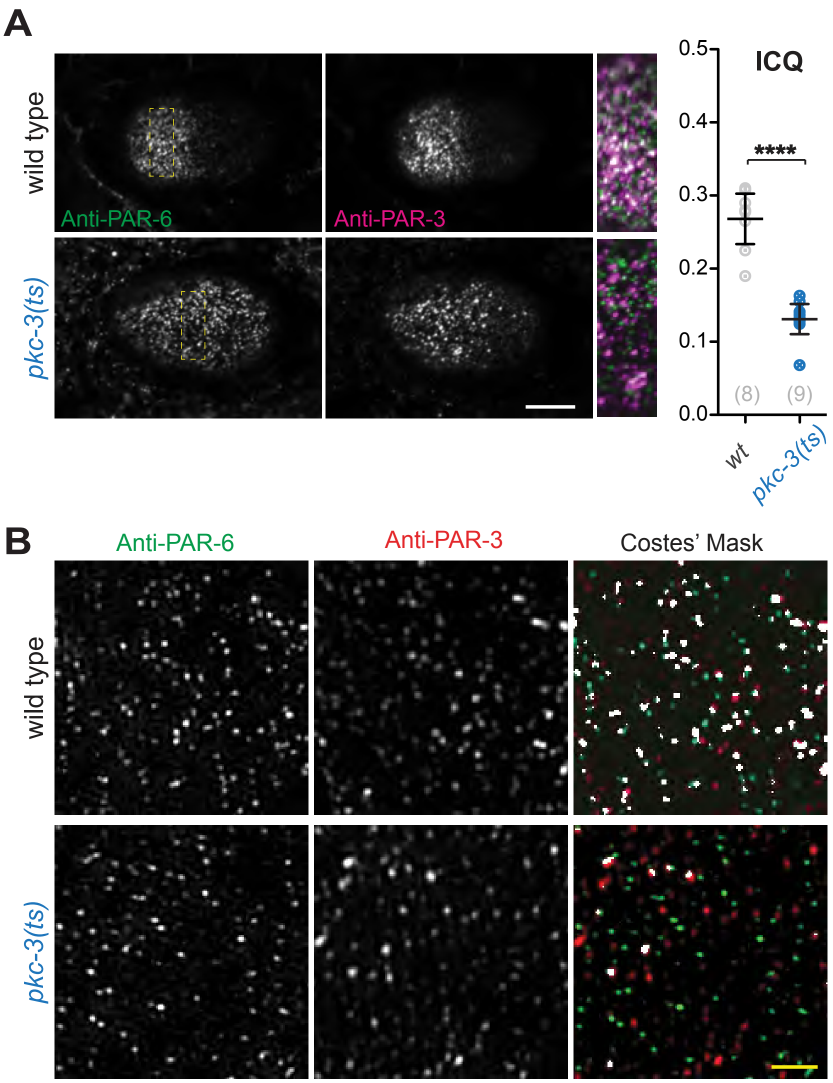

Figure S3. PAR-6 and PAR-3 colocalization is reduced in PKC-3-inhibited embryos, Related to Figure 2

(A) Cortical confocal images of wild type and *pkc-3(ts)* zygotes at late establishment phase stained for PAR-6 and PAR-3. Insets are magnifications of the ROIs delimited by dashed-line rectangles in PAR-6 image and show the overlap (white) of PAR-6 and PAR-3 in the anterior cortex. Scale bar: 10µm. Graphs shows the intensity correlation quotient (Li et al., 2004, Mean±CI 95%) between PAR-6 and PAR-3 in wild type (n=8) and *pkc-3(ts)* (n=9) zygotes.****p<0.0001.

(B) Super resolution images of wild type and *pkc-3(ts)* zygotes of an anterior cortical region stained for PAR-6 and PAR-3. In Costes' Mask images (JaCOP, Fiji) white regions indicate highly probable regions of colocalization (p-value of 100%). Scale bar: 2 µm.

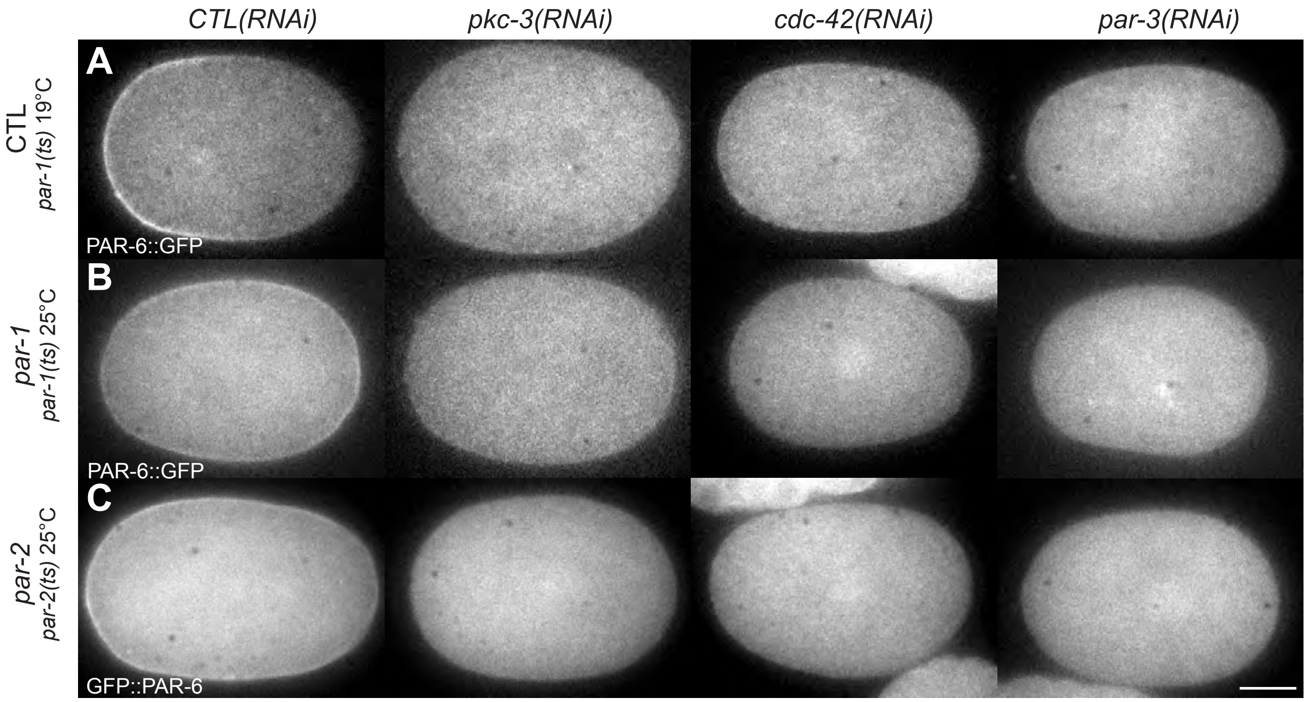

Figure S4. PAR-6 membrane localization depends directly on PAR-3, PKC-3 and CDC-42, Related to Figure 2

(A-C) Representative midsection confocal fluorescent images of PAR-6::GFP (A-B) and GFP::PAR-6 (C) expressing zygotes captured at nuclear envelope breakdown in the respective mutant backgrounds (*par-1(ts)*, A-B; *par-2(ts)*, C) at the permissive (19ºC, A) or restrictive (25ºC, B-C) temperature in combination with RNAi targeting *pkc-3*, *cdc-42* and *par-3* as indicated. Note that at the permissive temperature (CTL, first row), PAR-6 fails to localize to the membrane in *pkc-3(RNAi)*, *cdc-42(RNAi)* or *par-3(RNAi)* embryos. Membrane localization is not rescued when shifted to the restrictive temperature to inactivate PAR-1(B) or PAR-2(C) suggesting the failure of PAR-6 to bind membrane is not due to invasion of PAR-1 / PAR-2 into the anterior. Scale bar:10µm.

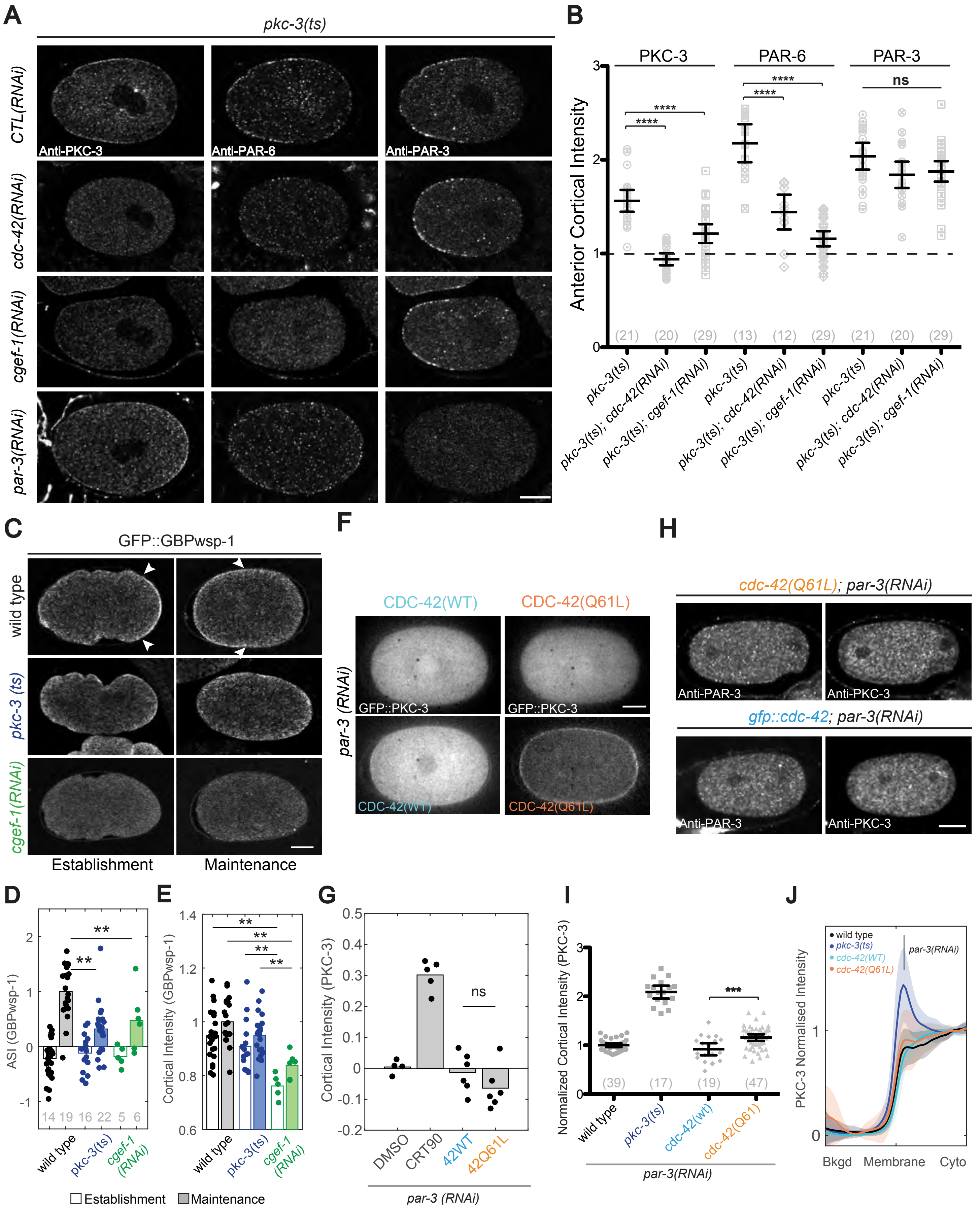

A
pkc-3(ts)
CTL(RNAi)
cdc-42(RNAi)
cgef-1(RNAi)
par-3(RNAi)
Anti-PKC-3
Anti-PAR-6
Anti-PAR-3
B
PKC-3
PAR-6
PAR-3
Anterior Cortical Intensity
****
****
****
****
ns
(21) (20) (29) (13) (12) (29) (21) (20) (29)
pkc-3(ts)
pkc-3(ts); cdc-42(RNAi)
pkc-3(ts); cgef-1(RNAi)
C
GFP::GBPwsp-1
wild type
pkc-3 (ts)
cgef-1(RNAi)
Establishment
Maintenance
F
CDC-42(WT)
CDC-42(Q61L)
par-3 (RNAi)
GFP::PKC-3
H
cdc-42(Q61L); par-3(RNAi)
Anti-PAR-3
Anti-PKC-3
gfp::cdc-42; par-3(RNAi)
D
ASI (GBPwsp-1)
**
**
14 19 16 22 5 6
wild type
pkc-3(ts)
cgef-1 (RNAi)
Establishment
Maintenance
E
Cortical Intensity (GBPwsp-1)
**
**
**
**
G
Cortical Intensity (PKC-3)
ns
DMSO
CRT90
42WT
42Q61L
par-3 (RNAi)
I
Normalized Cortical Intensity (PKC-3)
***
(39) (17) (19) (47)
wild type
pkc-3(ts)
cdc-42(wt)
cdc-42(Q61)
par-3(RNAi)
J
wild type
pkc-3(ts)
cdc-42(WT)
cdc-42(Q61L)
par-3(RNAi)
PKC-3 Normalised Intensity
Bkgd
Membrane
Cyto

Figure S5. Impact of CDC-42/GTP on membrane loading of anterior PARs, Related to Figure 3

(A) Midsection confocal images of zygotes showing that in *pkc-3(ts)* zygotes membrane localisation of PAR-6 and PKC-3, but not PAR-3, is dependent on CDC-42/GTP. *pkc-3(ts)* embryos are stained for PKC-3, PAR-6 and PAR-3. Representative images of control RNAi, top row, and defective phenotypes observed after RNAi of *cdc-42*, *cgef-1* or *par-3* are shown as indicated.

(B) Scatter plot of PKC-3, PAR-6 and PAR-3 anterior cortical intensities for datasets represented in (A). For each embryo (dot), the cortical intensity is divided by the corresponding PAR cytoplasmic intensity (mean±CI 95%). Values greater than 1 indicate presence at the cortex. See STAR Methods for details.

(C-E) Representative midsection confocal images (C), ASI quantifications (D), and normalized cortical intensity (E) for live wild-type, *pkc-3(ts)*, or *cgef-1(RNAi)* zygotes expressing the CDC-42/GTP-binding domain of WSP-1 (GBPwsp1), which monitors CDC-42 activity. White arrowheads highlight boundaries of spatial CDC-42 activity enrichment in wild type. Note that active CDC-42/GTP is localized uniformly to the membrane in PKC-3-inhibited zygotes (D) and shows similar levels to those present in wild-type (E). Therefore, CDC-42/GTP is in position to support PAR-6/PKC-3 anchoring at the membrane. Dataset for RNAi targeting *cgef-1* (CDC-42-GEF) is included for comparison. N (grey numbers) indicated in (D) and is the same for corresponding data in (E).

(F) Representative midsection confocal images of live *par-3(RNAi)* embryos expressing CDC-42(WT, mCherry) or CDC-42(Q61L, mCherry).

(G) Quantification of datasets represented in (F), which show no detectable rescue of GFP::PKC-3, despite observing significant enrichment of CDC-42(Q61L) at the membrane compared to wild-type CDC-42, suggesting we are stabilizing CDC-42 at the membrane. Note this analysis used heterozygous worms due to difficulties obtaining non-silenced homozygotes after crossing to a GFP::PKC-3 strain, which may account for the difference compared to (H-J). Intensity is normalized as in Figure 3D to *control(RNAi)* + CRT90 to facilitate comparison between the two figures.

(H) Representative midsection confocal images of *cdc-42(Q61L)* and *cdc-42(WT)* embryos co-stained for PAR-3 and PKC-3 after *par-3(RNAi)*.

(I) PKC-3 cortical intensity is normalized to cytoplasm and to wild type samples processed on the same day to account for variation between days (mean±CI 95%). Wild type (n=39), *pkc-3(ts)* (n=17), *cdc-42(WT)* (n=19) and *cdc-42(Q61L)* (n=47). Note that upon *par-3(RNAi)* in *cdc-42(Q61L)* embryos, we observe only a minor rescue of PKC-3 membrane localization compared to *pkc-3(ts)*. Thus, while increasing CDC-42/GTP levels can bias the system towards CDC-42-dependent PKC-3 assemblies (see Figure 5F-K), the bulk of PKC-3 membrane loading remains dependent on PAR-3, unlike in PKC-3-inhibited embryos.

(J) Comparison of membrane profiles of PKC-3 for the embryos in (I), highlighting membrane signal, showing mean ± SD. Briefly, normalized intensity profiles were extracted as 60 pixel stripes encompassing the embryo membrane, which was straightened using ImageJ. A 60x60px area in the region of peak PKC-3 membrane signal was projected in x to give a cross-section profile spanning background (Bkgd), crossing the membrane/cortex and into the cytoplasm (Cyto), which was then normalized such that background is set to 0 and cytoplasm to 1. Although *cdc-42(Q61L)* embryos show a minor peak of PKC-3 at the membrane, the magnitude of this peak in *cdc-42(Q61L)* embryos is significantly reduced compared to *pkc-3(ts)*. All analysis in (I-J) was limited to embryos with no detectable PAR-3 at the membrane.

**p<0.01, ***p<0.001, ****p<0.0001. Scale bars: 10μm.

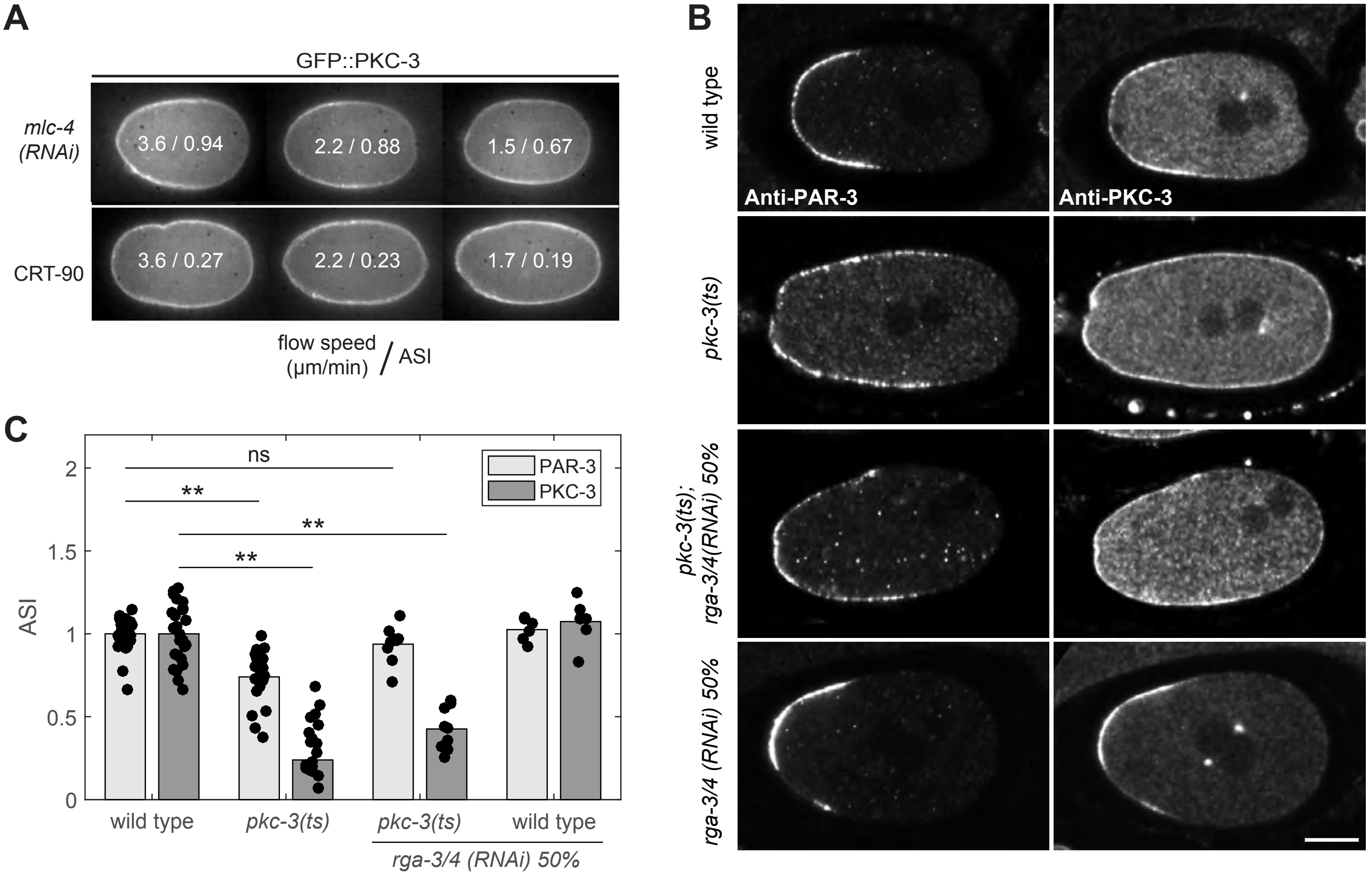

Figure S6. Analysis of the role of actomyosin flow and PAR segregation in wild type and PKC-3-inhibited embryos, Related to Figure 5

(A) Midsection confocal images of *mlc-4(RNAi)* and CRT90 treated embryos, matched by measured flow speed. Note all CRT90-treated embryos exhibit similar loss of asymmetry across all three flow rates, whereas *mlc-4(RNAi)* embryos retain asymmetry, though it decreases somewhat as flow rates are reduced. Flow rate (µm/min) / ASI are shown within each embryo.

(B) Representative midsection confocal images of maintenance phase wild-type or *pkc-3(ts)* embryos with or without partial RNAi-mediated depletion of the RhoGAPs RGA-3/4. Embryos were fixed and stained for both PAR-3 and PKC-3. Note that segregation of PAR-3 is fully rescued in *pkc-3(ts)* by *rga-3/4(RNAi)*, whereas PKC-3 remains present at the posterior. Scale bar: 10µm.

(C) ASI quantification of the full dataset represented in (B) reveals failure of *rga-3/4(RNAi)* to rescue PKC-3 asymmetry despite rescue of PAR-3 ASI to wild-type levels. ASI is shown normalized for each protein individually compared to N2. (N: wild type = 25, *pkc-3(ts)* = 22, *pkc-3(ts); rga-3/4(RNAi) 50% = 9,* wild type; *rga-3/4(RNAi)50% = 6)*

**p<0.01. Scale bars: 10µm.

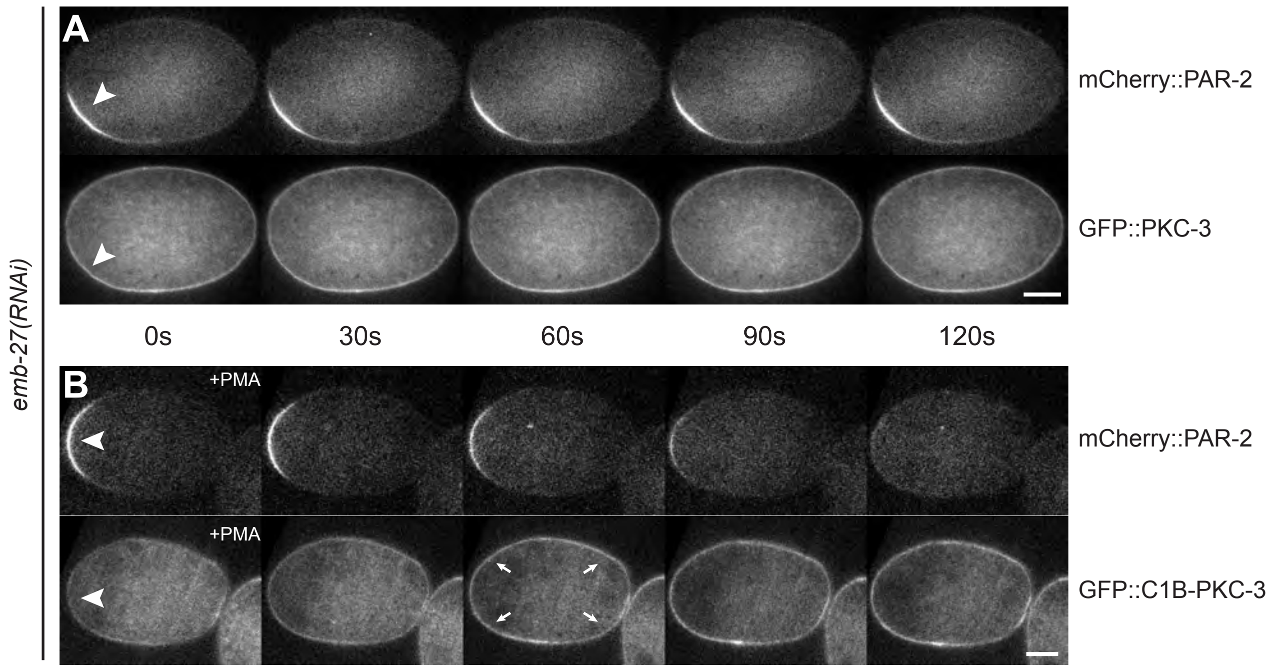

Figure S7. Anterior meiotic PAR-2 domains are rapidly cleared by targeting PKC-3 to the membrane, Related to Figure 6

(A) Midsection fluorescent images of *emb-27(RNAi)* zygotes expressing GFP::PKC-3 with mCherry::PAR-2. *emb-27(RNAi)* induces defects in meiotic progression, leading to inverted polarity with an anterior domain of PAR-2 near the meiotic spindle remnant, similar to *mei-1(RNAi)* (Wallenfang and Seydoux, 2000). This anterior meiotic domain remains on the membrane for more than 30 min (n=3).

(B) Same as (A), but zygotes express GFP::C1B-PKC-3, which is targeted to the membrane by the addition of PMA. (B). The increase in PKC-3 at the membrane rapidly clears the anterior PAR-2 domain upon PMA addition (3.2±1.7min, n=3). Arrows indicate enhanced PKC-3 membrane localisation 60 s after PMA addition.

Note that neither GFP::PKC-3 or C1B::GFP::PKC-3 are fully excluded by the PAR-2 domain (arrowheads). Scale bar: 10µm.
